# Enhancing the Anticancer Potential of Targeting Tumor-Associated Metalloenzymes via VEGFR Inhibition by New Triazolo[4,3-*a*]pyrimidinone Acyclo C-Nucleosides Multitarget Agents

**DOI:** 10.3390/molecules27082422

**Published:** 2022-04-08

**Authors:** Mohamed Nabil Abd Al Moaty, El Sayed Helmy El Ashry, Laila Fathy Awad, Nihal Ahmed Ibrahim, Marwa Muhammad Abu-Serie, Assem Barakat, Mezna Saleh Altowyan, Mohamed Teleb

**Affiliations:** 1 Chemistry Department, Faculty of Science, Alexandria University, Alexandria 21321, Egypt; mohamednabil_sc_chem@yahoo.com (M.N.A.A.M.); eelashry60@hotmail.com (E.S.H.E.A.); nihalawadallah@outlook.com (N.A.I.); 2 Medical Biotechnology Department, Genetic Engineering and Biotechnology Research Institute, City of Scientific Research and Technological Applications (SRTA-City), Alexandria 21934, Egypt; marwaelhedaia@gmail.com; 3 Department of Chemistry, College of Science, King Saud University, P.O. Box 2455, Riyadh 11451, Saudi Arabia; 4 Department of Chemistry, College of Science, Princess Nourah bint Abdulrahman University, P.O. Box 84428, Riyadh 11671, Saudi Arabia; msaltowyan@pnu.edu.sa; 5 Department of Pharmaceutical Chemistry, Faculty of Pharmacy, Alexandria University, Alexandria 21521, Egypt; mohamed.t.ismail@alexu.edu.eg

**Keywords:** MMP-2, CAII, VEGFR-2, multitarget anticancer agents, 1,2,4-triazolo[4,3-*a*]pyrimidinone acyclo C-nucleosides

## Abstract

The role of metalloenzymes in tumor progression had broadened their application in cancer therapy. Of these, MMPs and CAs are validated druggable targets that share some pivotal signaling pathways. The majority of MMPs or CAs inhibitors are designed as single-target agents. Despite their transient efficacy, these agents are often susceptible to resistance. This set the stage to introduce dual inhibitors of correlated MMPs and CAs. The next step is expected to target the common vital signaling nodes as well. In this regard, VEGFR-2 is central to various tumorigenesis events involving both families, especially MMP-2 and CA II. Herein, we report simultaneous inhibition of MMP-2, CA II, and VEGFR-2 via rationally designed hybrid 1,2,4-triazolo[4,3-*a*]pyrimidinone acyclo C-nucleosides. The promising derivatives were nanomolar inhibitors of VEGFR-2 (**8**; IC_50_ = 5.89 nM, **9**; IC_50_ = 10.52 nM) and MMP-2 (**8**; IC_50_ = 17.44 nM, **9**; IC_50_ = 30.93 nM) and submicromolar inhibitors of CA II (**8**; IC_50_ = 0.21 µM, **9**; IC_50_ = 0.36 µM). Docking studies predicted their binding modes into the enzyme active sites and the structural determinants of activity regarding substitution and regioselectivity. MTT assay demonstrated that both compounds were 12 folds safer than doxorubicin with superior anticancer activities against three human cancers recording single-digit nanomolar IC_50_, thus echoing their enzymatic activities. Up to our knowledge, this study introduces the first in class triazolopyrimidinone acyclo C-nucleosides VEGFR-2/MMP-2/CA II inhibitors that deserve further investigation.

## 1. Introduction

The tumor microenvironment has sparked considerable interest in cancer research. It provides the conducive medium for tumor growth and innately fosters different tumor progression events via a plethora of mediators [1] of which many enzymes were validated as druggable anticancer targets as exemplified by the tumor-associated metalloenzymes: matrix metalloproteinases (MMPs) and carbonic anhydrases (CAs). MMPs are a family of zinc-dependent endopeptidases comprising twenty-six isoforms [2] classified as collagenases, gelatinases, stromelysins, matrilysins, membrane-type MMPs, and others [3]. MMPs are dysregulated in almost all human tumors [4,5], promoting extracellular matrix turnover, angiogenesis, and tumor growth and metastasis [6,7]. No surprise then, numerous MMPs inhibitors have been developed over the last decades [8,9,10,11,12]. The collagenase MMP-2 is a prime example of how an individual member of the MMPs family contributes to cancer progression via a vast repertoire of extracellular matrix and non-extracellular matrix substrates, raising questions about its implication for every step of the tumor metastatic cascade [13,14,15,16]. Most importantly, MMP-2 can promote angiogenesis by inducing the expression of the vascular endothelial growth factor (VEGF) or processing the factors regulating its bioavailability [17,18]. MMP-2 can further induce angiogenesis in the tumor microenvironment by cleaving the extracellular matrix type IV collagen and coordinating with αvβ3 integrins [19,20].

Another tumor-associated metalloenzymes family that has been extensively implicated in modulating the tumor microenvironment is the CAs family [21], comprising 15 zinc-dependent isoforms that catalyze the reversible hydration of carbon dioxide to bicarbonate anion. The identified mammalian CAs include cytoplasmic, mitochondrial, membrane-associated and CA-related proteins [22]. The cytoplasmic CA II has recently become one of the hot research topics, being a novel biomarker and potential anticancer target [23,24]. It was overexpressed in various tumors [25,26,27,28,29,30,31], and its overexpression had been correlated with tumor aggressiveness. Mechanistic studies demonstrated that CA II, in particular, supports the survival of the tumor blood endothelial cells under lactic acidosis in the tumor microenvironment, rendering the tumor endothelial cells well equipped for the microenvironment’s harsh conditions. This unique function of CA II addresses the significance of CA II inhibition for halting angiogenesis and suggests CA II to be a promising antiangiogenic target. Further investigations confirmed that the CA II expression in the tumor endothelium is signaled via the tumor-derived VEGFR-2 [23].

**Figure 1:**
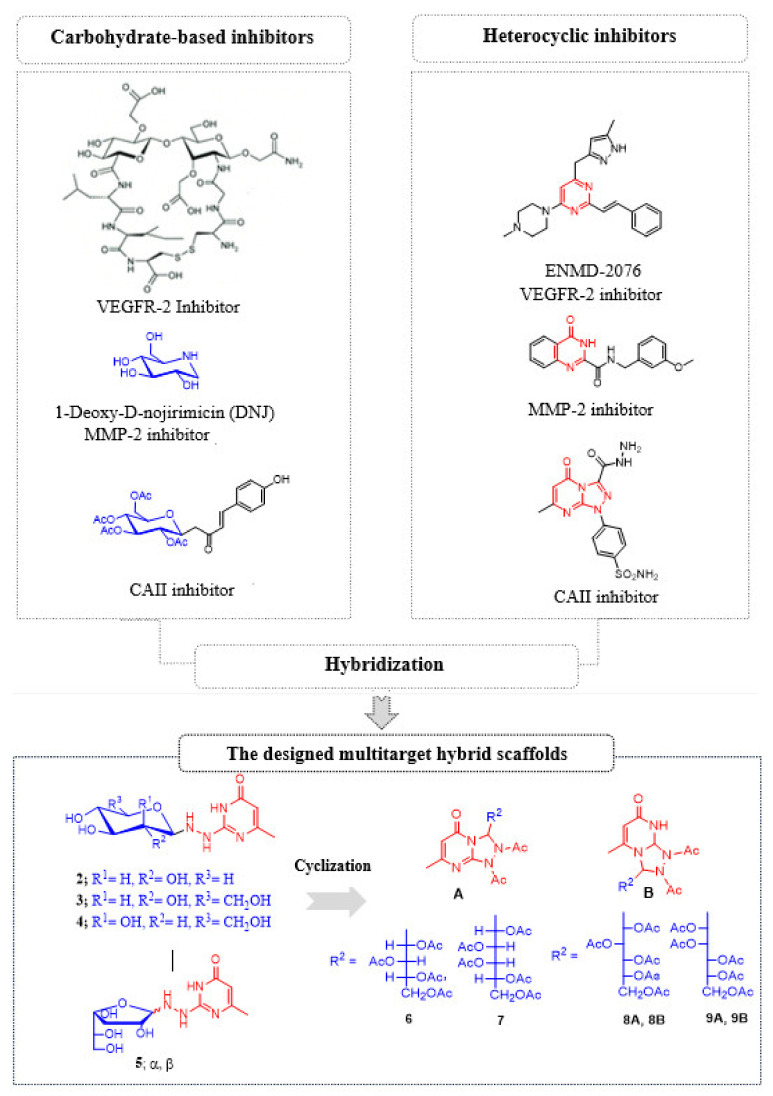
Lead inhibitors [44,47,49,52,53,62,63] and design of the target compounds.

Taken together, it is obvious that VEGF/VEGFR-2 is a central signaling node that significantly contributes to the tumor-promoting effects of MMPs and CAs, especially MMP-2 and CA II. In addition, VEGF/VEGFR drives cancer-initiating stem cells [32] and increases the intratumor vessels permeability forming complex, disorganized, and leaky vasculature that hinder efficient delivery of anticancer agents to the tumor microenvironment [33,34]. Therefore, it seems that the simultaneous intervention of the interplay between VEGFR-2, MMP-2, and CA II by multitarget inhibitors can be an optimal strategy to maximize the therapeutic potential by avoiding the resistance to single-target inhibitors [23,35,36,37,38] or combination therapies drawbacks. A literature survey revealed some reports about the dual inhibitors of CA II and MMPs [24,39,40,41,42,43]. However, to the best of our knowledge, there is no single molecule targeting VEGFR-2 as well. The need to tailor such multitarget molecules sparked our interest. Inspired by the principle of mimicking VEGFR-2 peptide inhibitors by active carbohydrate scaffolds [44], “the sugar approach” design of various potent CA inhibitors [45], and the widespread development of carbohydrate-based MMPs [46], we proposed tethering various carbohydrate appendages to pharmacophoric motifs and/or isosteres of various individual inhibitors for tailoring hybrid multitarget scaffolds (Figure 1). In this regard, pyrimidine and triazolopyrimidine cores were selected being well-represented motifs in several lead inhibitors [47,48,49,50,51,52,53]. Structure diversification of the carbohydrate substituents, as well as the hydrazone linker flexibility, will generate two series of derivatives for gaining more information about the structure–activity relationship. All derivatives were preliminarily screened via 3-(4,5-dimethylthiazol-2-yl)-2,5-diphenyl-2*H*-tetrazolium bromide (MTT) assay for their cytotoxic activities on normal fibroblasts (Wi-38) and three common human cancers, Caco-2, MDA-MB 231, and HepG-2, as indicated by the 2020 global cancer statistics [54]. The most promising derivatives regarding anticancer activity and selectivity were in vitro evaluated for their inhibitory activities against MMP-2, CA II, and VEGFR-2 which are crucial in various carcinogenesis events of the selected cancer types [25,28,55,56,57,58,59,60,61].

Docking was conducted to predict their pharmacophoric features addressing the key interactions with the enzymatic catalytic domains and correlating their binding modes to those of reference inhibitors.

## 2. Results and Discussion

### 2.1. Chemistry

**Scheme 1:**
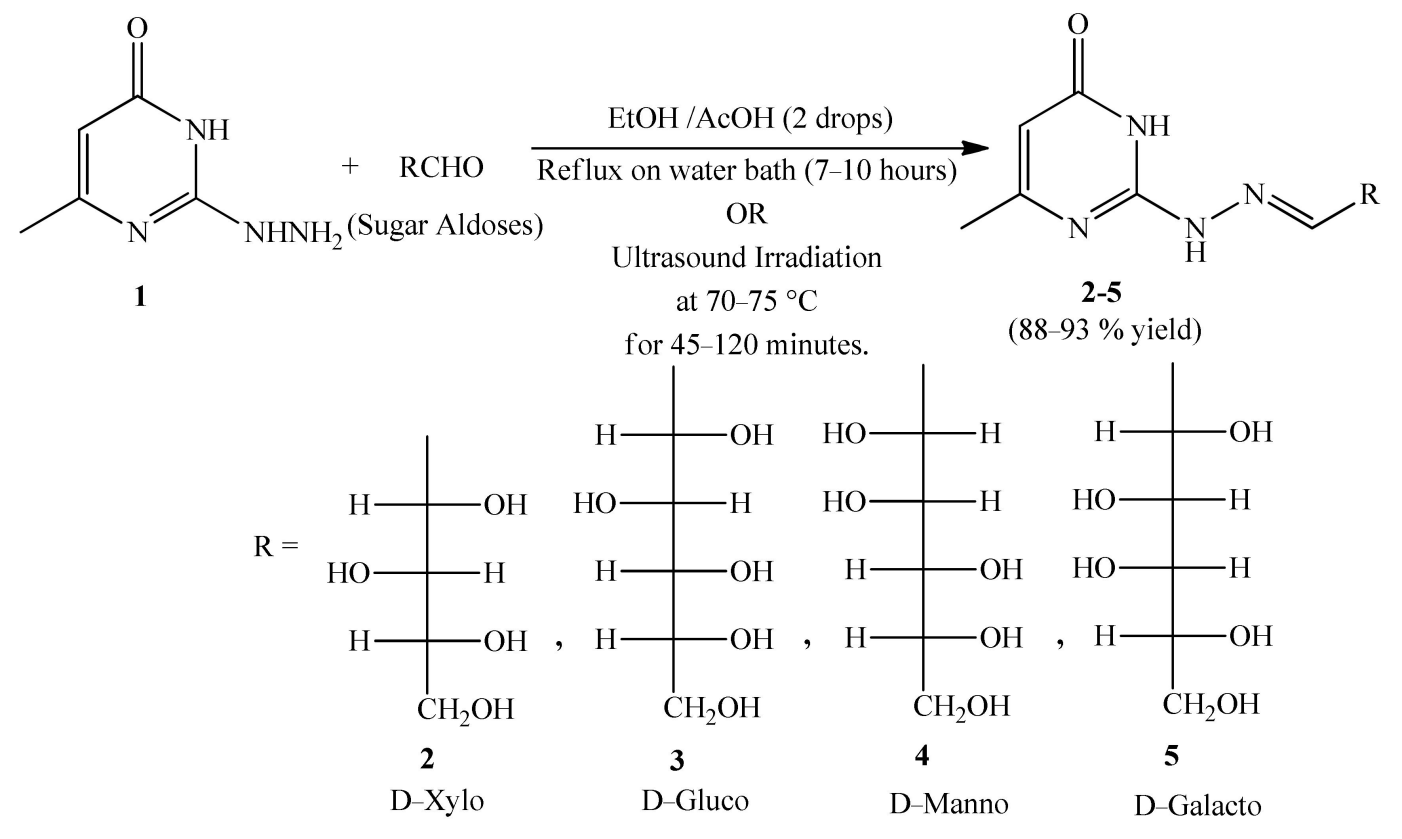
Reaction of the hydrazine derivative **1** with sugar aldoses.

Condensation of aldoses with amino compounds can be attempted under mild conditions with deprotected hydroxyl groups or through activation of the anomeric carbon. Thus, condensation of 2-hydrazinyl-6-methylpyrimidin-4-one **1** with different aldoses [64] (D-xylose, D-glucose, D-mannose, and D-galactose) in ethanol under reflux for 7–10 h afforded the corresponding hydrazones **2**–**5**, respectively. However, when the reaction was established using ultrasound irradiation, better yields within a shorter time were observed (Scheme 1). The ultrasound irradiation at 75 °C reduces the reaction time to 45–120 min compared to the conventional methods (Table 1).

**Scheme 2:**
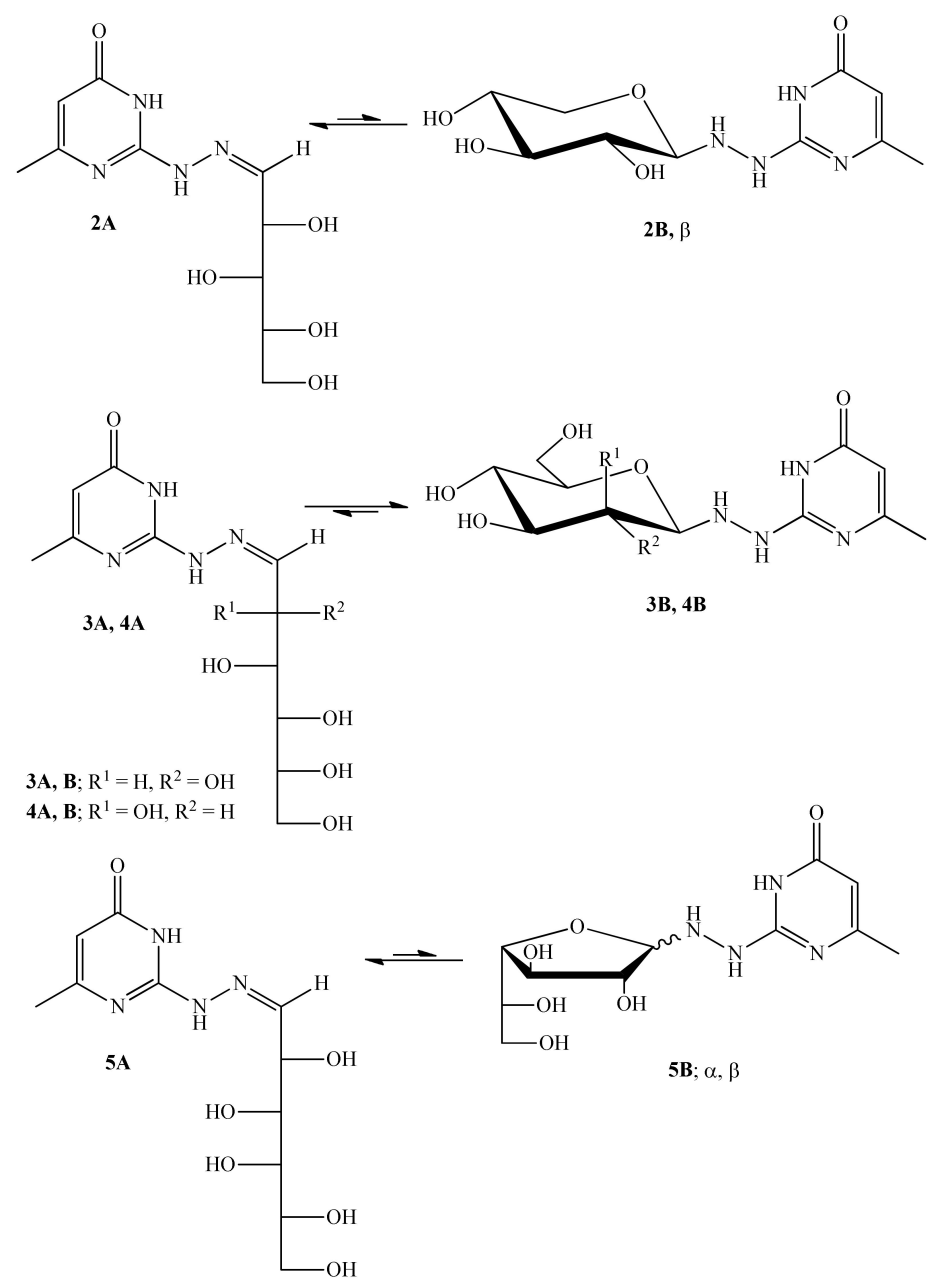
Equilibration of the acyclic and cyclic nature of sugar hydrazones **2**–**5**.

Sugar hydrazones can exist in an equilibrium between the acyclic Schiff’s bases and the cyclic *N*-glycosyl hydrazine derivatives, which can adapt either a pyranose or furanose ring with different anomeric configurations (*α* or *β*). The nature of the acyclic-cyclic equilibrium mixture that exists in solution is highly influenced by the effect of the solvent, the nature of the sugar, and the basicity of the reacted hydrazine [65,66,67,68]. Structure elucidation of the synthesized products (**2**–**5**) was deduced from their spectral analyses: ^1^H-, ^13^C-NMR, ^1^H-^1^H DQF COSY and ^1^H-^13^C HMQC experiments. These products showed different behavior in solution of DMSO-*d_6_* depending on the nature of the sugar. Thus, the spectral characterization of **2** in a solution of DMSO-*d_6_* showed its existence in an equilibrium mixture of acyclic hydrazones **2A** and the cyclic pyranosyl hydrazine analog **2B** in a 3:1 ratio (Scheme 2). This investigation was based on the presence of a doublet signal resonated at δ_H_ 7.49 ppm with 0.75 proton intensity corresponding to the azomethine proton (CH=N); this signal was correlated with its carbon resonated at δ_C_ 148.6 ppm. On the other hand, the cyclic *β*-D-xylopyranosyl hydrazinyl derivative **2B** was deduced from the doublet signal corresponding to the anomeric H-1′ which resonated at δ_H_ 3.76 ppm (*J*_1′,2′_ = 8.8 Hz) (after addition of D_2_O to overcome the overlap of the signals of NH and OH). This large *J*_1′,2′_ value confirmed the antiparallel relationship between H-1′ and H-2′. The later signal was correlated to C-1′ signal assigned at δ_C_ 92.2 ppm in ^13^C-NMR spectrum. Moreover, the pyranose structure was deduced from the signal corresponding to C-4′ observed at δ_C_ 70.0 ppm and agreed with that reported in the literature [69,70]. Moreover, other carbons and protons were fully assigned (Experimental Section).

On the other hand, spectral analysis of **3** in DMSO-*d_6_* confirmed the presence of an equilibrium between the cyclic *β*-D-glucopyranosyl hydrazine derivative **3B** and the acyclic hydrazone **3A** in 6:1 ratio. By time, this equilibrium was shifted to the formation of only the cyclic structure **3B**. This was clearly observed from the ^1^H-NMR spectrum (DMSO-*d_6_* + D_2_O) which showed the presence of signals characteristic for the cyclic glucopyranosyl derivative **3B** only. A remarkable *β*-configuration of the pyranosyl hydrazine **3B** was deduced from the large coupling constant *J*_1′,2′_ = 8.8 Hz of the doublet signal corresponding to the H-1′ which was resonated at δ_H_ 3.79 ppm and correlated with carbon signal at δ_C_ 91.2 ppm. Moreover, the signal of C-5 observed at δ_C_ 78.1 ppm confirmed the pyranose ring structure of **3B**.

The D-mannose hydrazone derivative **4** exists in an equilibrium mixture, acyclic structure **4A** and cyclic D-mannopyranosyl hydrazine derivative **4B**, in a 6:1 ratio. The pyranosyl ring structure was deduced from the assignment of C-5′ resonated at δ_C_ 78.5 ppm and in correlation with the triplet at δ_H_ 3.06 ppm corresponding to H-5′. The anomeric carbon was observed at δ_C_ 88.1 ppm and correlated with the doublet signal corresponding to H-1′ at δ_H_ 4.01 ppm with coupling constant *J*_1′, NH_ = 10.8 Hz. However, the presence of H-2′ as a broad singlet confirmed the existence of the cyclic product **4B** in the *β*-configuration with 1,2-cis disposition of both H-1′ and H-2′ with small coupling constant [71,72]. The acyclic nature of **4A** was confirmed from the doublet signal corresponding to the azomethine proton at δ_H_ 7.66 ppm which correlated with its carbon signal at δ_C_ 149.9 ppm. Moreover, the assignment of C-5′ at δ_C_ 71.4 ppm which was correlated with the multiplet signal of H-5′ at δ_H_ 3.48 ppm also confirmed the acyclic nature of **4A**.

The D-galactose hydrazone derivative **5** exists in an equilibrium mixture, acyclic Schiff’s base **5A** and the cyclic isomer **5B** in 3:2 ratio. However, *α*-D-galactofuranosyl ring structure of **5B** was deduced based on the signal corresponding to H-4′ resonated as a doublet signal at δ_H_ 3.36 ppm with coupling constant *J*_4′,3′_ = 2.8 Hz. This was correlated with the carbon signal assigned at higher frequency region δ_C_ 76.9 ppm. The upfield shift of both C-3′ (δ_C_ 71.2 ppm) and C-5′ (δ_C_ 70.3 ppm) also agreed with the furanose ring structure. The large coupling constant *J*_1′,2′_ = 7.6 Hz of the vicinal H-1′ and H-2′ (measured after the addition of D_2_O) confirmed the cis *α*-configuration, whereas the trans 1,2-orientation would require a smaller value [73,74,75]. A major signal of C-1′ in the α-anomer was assigned at δ_C_ 92.0 ppm and correlated with its H-1′ that resonated at δ_H_ 3.66 ppm. However, the presence of a minor signal at δ_C_ 94.6 ppm correlated to a doublet signal at δ_H_ 4.34 ppm could be a result of the existence of the *β* anomer. The smaller coupling constant *J*_1′,2′_ = 4.0 Hz confirmed the trans antiparallel configuration between H-1′ and H-2′. On the other hand, the acyclic structure **5A** was deduced from the doublet signal of the azomethine proton which was assigned at δ_H_ 7.45 ppm and was correlated with carbon signal at δ_C_ 149.6 ppm.

Annulation of sugar hydrazones **2**–**5** to 1,2,4-triazolo[4,3-*a*]pyrimidinone acyclo C-nucleosides **6**–**9** was carried out using boiling acetic anhydride for 1–3 h. Their structural elucidation was confirmed by their NMR spectral analysis. Thus, NMR spectra of these compounds showed disappearance of signals corresponding to both the azomethine and the anomeric protons in their precursors as well as their corresponding carbon signals. Consequently, signals corresponding to the acetoxy groups of the alditolyl moieties were assigned at the upfield region in addition to the two N-acetyl protons signals (*N*-1 and *N*-2 of the triazole moiety). Moreover, a new characteristic signal corresponding to the 1,2,4-triazole’s H-3 was assigned as a singlet signal at δ_H_ 6.40–6.74 ppm which correlated with its carbon at δ_C_ 67.6–72.1 ppm. From our previous studies performed on the aromatic aldehydes [50], the kinetically controlled regioisomer 5-methyl-7-oxo-1,2,4-triazolo[4,3-*a*]pyrimidine derivative was formed first, and it underwent Dimroth rearrangement under the effect of either light or acidic medium to give the more stable thermodynamically regioisomer 7-methyl-5-oxo-1,2,4-triazolo[4,3-*a*]pyrimidine derivative.

**Scheme 3:**
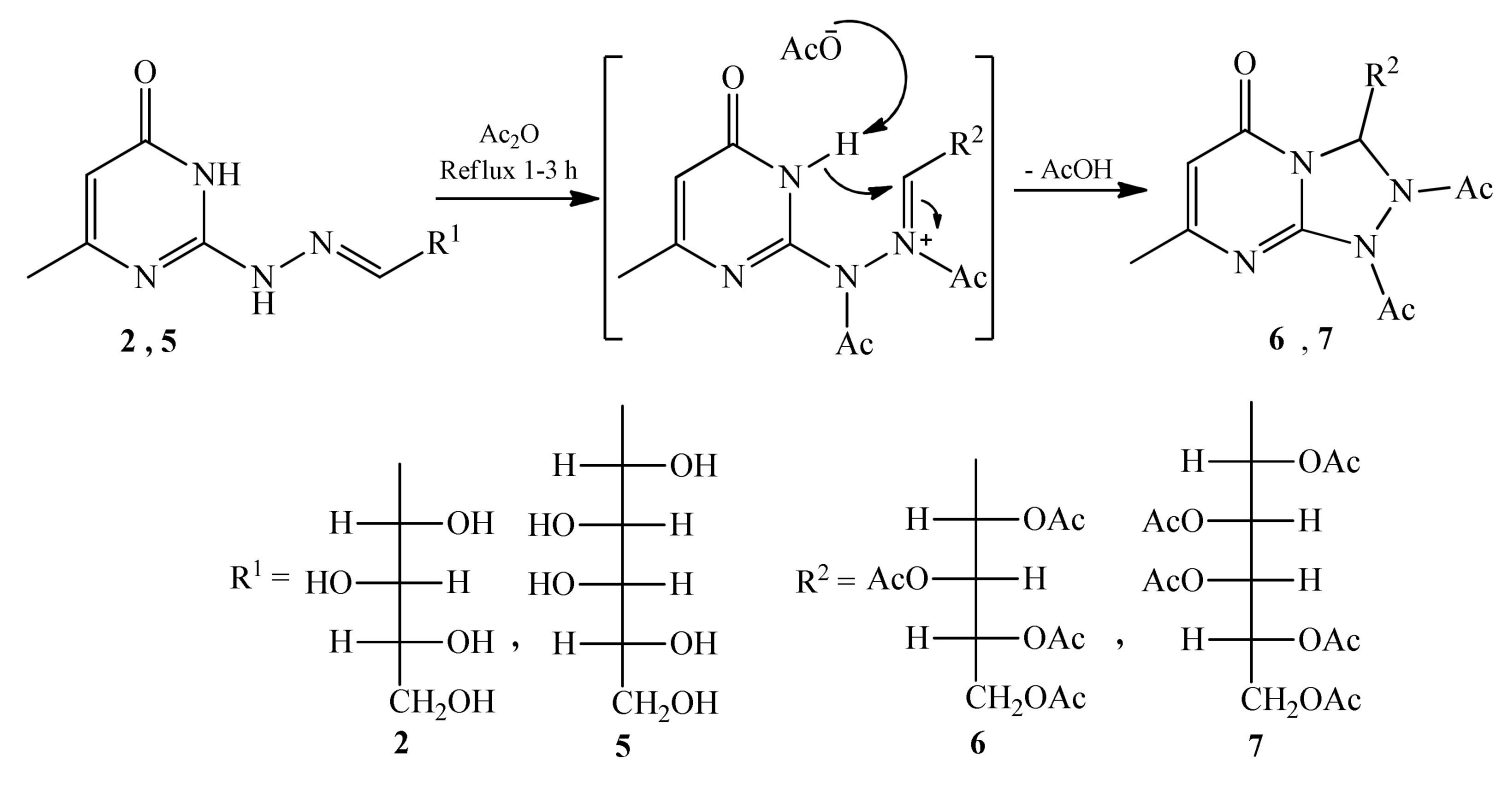
Annulation of sugar hydrazones **2** and **5** to 1,2,4-triazolo[4,3-*a*]pyrimidinone acyclo C-nucleosides **6** and **7**.

Accordingly, spectral analysis of the products **6** and **7** having the D-xylo and D-galacto configurations showed only one characteristic signal of pyrimidine H-6 that resonated at δ_H_ 5.30 and 5.23 ppm, respectively, and correlated with their carbons assigned at δ_C_ 98.2 and 97.4 ppm, respectively. In addition, the assignment of the 1,2,4- triazole’s H-3 to resonate at δ_H_ 6.44 and 6.59 ppm, respectively, correlated with the carbons resonated at δ_C_ 71.4 and 70.0 ppm, respectively. This agreed with the assigned structures of the 7-methyl-5-oxo-1,2,4-triazolo[4,3-*a*]pyrimidine regioisomers **6** and **7**, respectively (Scheme 3). The structure elucidation was in agreement with that reported in the literature for the isomeric 5-oxo- and 7-oxo-1,2,4 triazolopyrimidines [50,76] based on H-6, C-6, and carbonyl carbon chemical shifts in their NMR spectra [77,78,79]. Moreover, the heteronuclear multiple bond correlation (HBMC) analysis that was described in our previous work [50], for compounds that have the same 1,2,4-triazolopyrimidinone moiety, clarified and verified the structure.

**Scheme 4:**
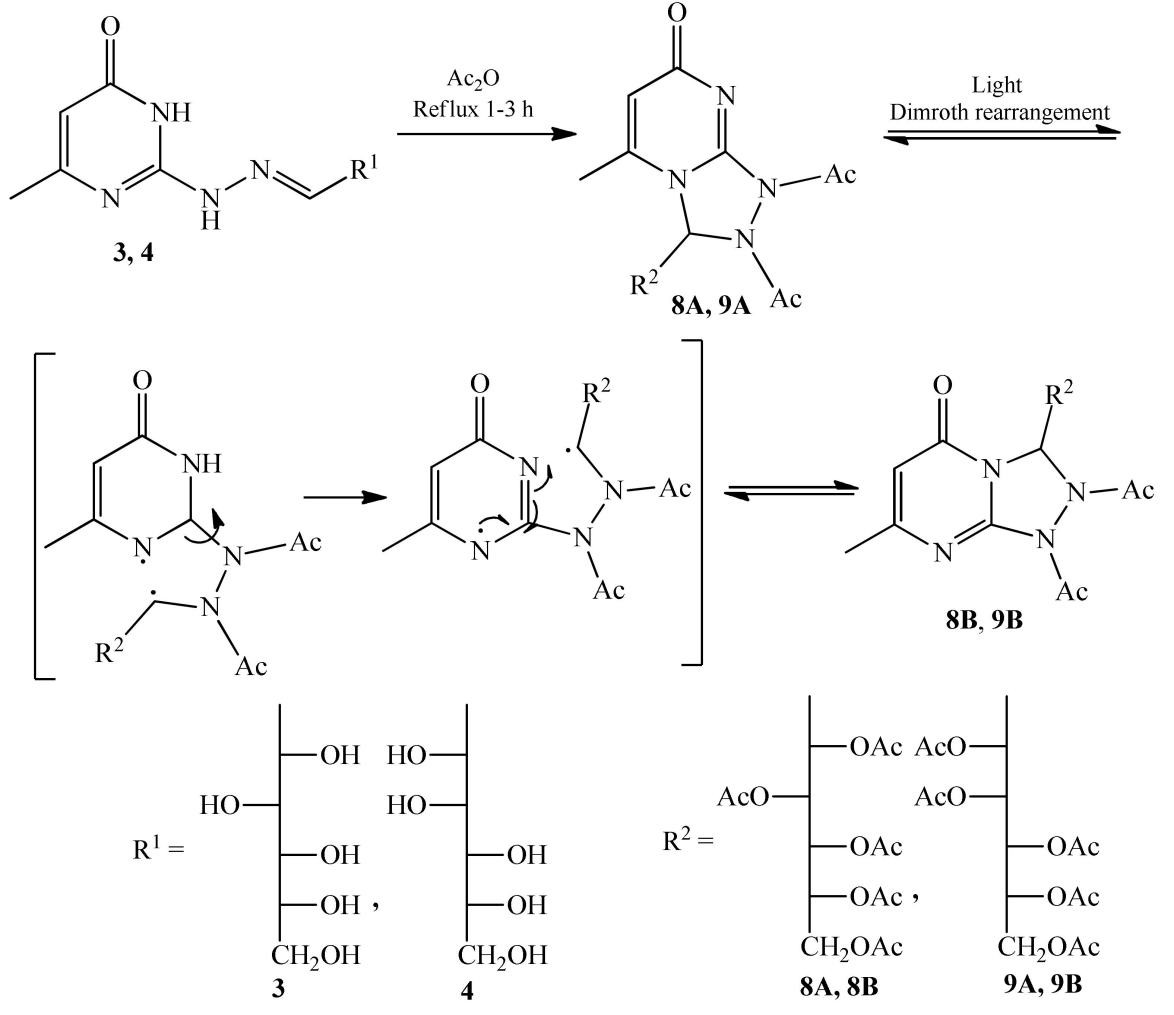
Annulation of sugar hydrazones **3** and **4** to 1,2,4-triazolo[4,3-*a*]pyrimidinone acyclo C-nucleosides **8**–**9**.

On the other hand, the ^13^C-NMR spectrum of the D-gluco acyclo C-nucleoside **8** (Scheme 4) showed signals at the downfield region at δ_C_ 98.07 and 108.62 ppm characteristic for C-6 of both regioisomers 5-methyl-7-oxo-1,2,4 triazolopyrimidine **8A** and 7-methyl-5-oxo-1,2,4 triazolopyrimidine **8B** in approximately equal ratio. Although only one pure product was isolated after column chromatography as that monitored by TLC for the collected eluted samples, the existence of a mixture of both regioisomers **8A** and **8B** was observed as a result of Dimroth rearrangement during NMR experiments of **8.** It is worth mentioning that despite the precautions taken to guard against light-promoted rearrangement of **8A** during sample handling, both isomers were detected as such rearrangement seems to be rapid and unavoidable.

^1^H-^13^C NMR (HMQC) spectrum (Supplementary Material S19) showed that both carbon signals of C-6 were correlated with their corresponding protons that resonated at δ_H_ 5.15 (overlapped with H-4′ of the sugar moiety) and 5.99 ppm, respectively. Moreover, signals corresponding to C-3 in both **8A** and **8B** are assigned at δ_C_ 67.6 and 71.6 ppm, respectively, and correlated with their respective H-3 protons which resonated at δ_H_ 6.40 and 6.23 ppm, respectively. Such assignment confirmed that the thermodynamically more stable 7-methyl-5-oxo-1,2,4 triazolopyrimidine isomer **8B** coexisted with the kinetically 5-methyl-7-oxo-1,2,3-triazolopyrimidine **8A**.

Under the same reaction conditions, the D-mannose hydrazone derivative **4** afforded the 5-methyl-7-oxo-1,2,4 triazolopyrimidine regioisomer **9A** rather than the 7-methyl-5-oxo-1,2,4 triazolopyrimidine thermodynamic isomer **9B** (Supplementary Material S20) (Scheme 4), and this was deduced from the NMR spectra of the isolated pure regioisomer **9A** eluted from column chromatography. Herein, the downfield shift of signals corresponding to H-6 as well as H-3 resonated at δ_H_ 6.08 and 6.74 ppm, respectively, and correlated to their carbon signals assigned at higher frequency at δ_C_ 109.1 and 68.1 ppm, respectively, confirming the formation of the kinetically 5-methyl-7-oxo-1,2,4-triazolopyrimidine regioisomer **9A** as a single isomer. However, exposure of **9A** to light for 63 h (the maximum time reported to obtain the more stable regioisomer) [50] afforded an equimolar ratio of the regioisomers 5-methyl-7-oxo-1,2,4 triazolopyrimidine **9A** and 7-methyl-5-oxo-1,2,4 triazolopyrimidine **9B**, where the HMQC spectrum (Supplementary Material S22) showed another extra signal corresponding to the 7-methyl-5-oxo-1,2,4 triazolopyrimidine regioisomer **9B**. Signals corresponding to H-6 and H-3 of isomer **9B** were observed at δ_H_ 5.31 and 6.49 ppm, respectively, and their carbons were assigned at δ_C_ 98.0 and 72.1 ppm, respectively. In addition, new signals for the alditolyl chain of **9B** were recorded. Therefore, possible Dimroth rearrangement has taken place under the effect of light, affording the thermodynamically 7-methyl-5-oxo-1,2,4 triazolopyrimidine isomer **9B**.

**Figure 2:**
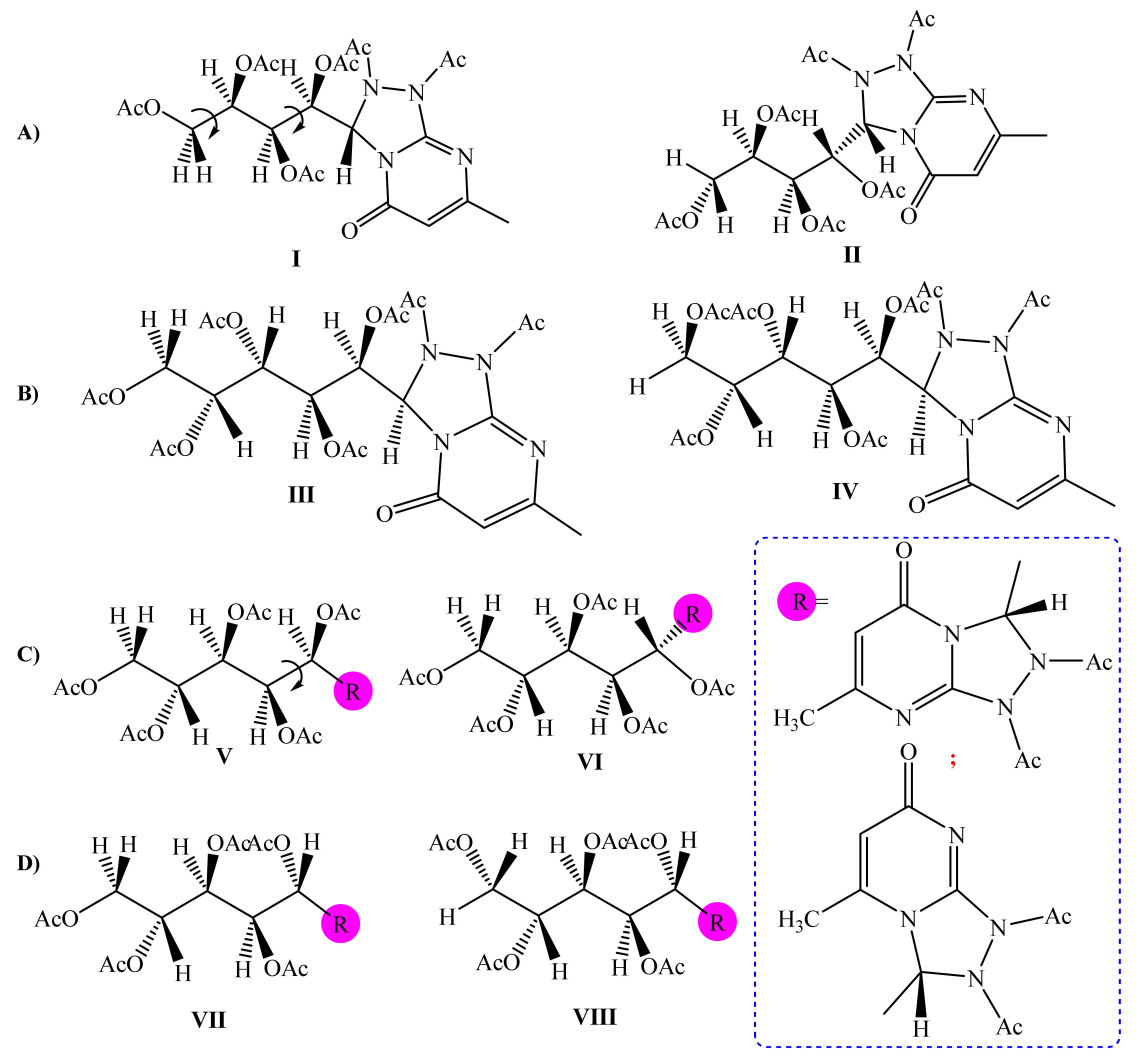
(**A**) Zigzag conformation of **6**, (**B**) zigzag conformation of **7**, (**C**) zigzag conformation of **8,** and (**D**) zigzag conformation of **9**.

The conformation of acylated and non-acylated carbohydrate derivatives [71,80] could be deduced from the vicinal proton-proton coupling constant. Therefore, the conformation of the acylated acyclo C-nucleoside **6** was deduced from the vicinal proton-proton coupling constants in their ^1^H-NMR spectra and represented in Figure 2. Thus, the extended zigzag conformation **II** represents the most favorable conformational structure of the D-xylo derivative **6** (Figure 2). Such conformation results from the rotation between C-1′ and C-2′ bond resulting an anti-parallel disposition of H-1′ and H-2′ and this was agreed with the observed coupling constant *J*_1′,2′_ = 8.8 Hz and *J*_1′,3′_ < 1 Hz. Moreover, the intermediate magnitude of *J*_4′’,3′_ = 4.8 Hz required rotation around C-3′-C-4′ bond to perform such value.

The Gauche conformations of H-1′-H-2′, as well as H-3′-H-4′ in the D-galacto derivative 7, were confirmed from their respective coupling constants, whereas the high coupling constant of *J*_2′,3′_ (8.8 Hz) was because of the anti-parallel disposition of H-2′ and H-3′. Therefore, the galacto derivative could exist in the favorable extended planar zigzag conformation **III** or **IV** [80].

Consequently, the high coupling constant *J*_1′,2′_ = 9.5 Hz observed for the D-gluco derivative **8A** indicates the antiparallel dispositions of H-1′and H-2′; this was verified by rotation around the C-1′ and C-2′ bond with the result of the sickle conformation [80]. The antiparallel conformation of H-3′, H-4′, as well as H-4′ and H-5′ and the Gauche orientations between H-2′-H-3′ and H-4′-H-5′ were confirmed from their respective coupling constants (experimental). Accordingly, the D-gluco derivative **8A** exists in the more stable zigzag conformational structure **VI**.

Similarly, the D-manno acyclo C-nucleoside **9A** existed in the more stable extended zigzag conformational structure **VIII** which resulted from the rotation around the C-4′-C-5′ bond to verify the intermediate magnitude of *J*_5′’,4′_ = 4.4 Hz. The coupling constants (experimental) of the other protons verify their dispositions.

### 2.2. Biological Evaluation

#### 2.2.1. Cytotoxicity Screening

All derivatives were subjected to preliminary cytotoxicity screening on normal human lung fibroblasts (Wi-38) for selectivity assessment, followed by anticancer evaluation against MDA-MB231, Caco-2, and HepG-2 cells compared to the reference multitarget chemotherapeutic agent doxorubicin (Dox) by MTT assay [81,82]. It is worth mentioning that previous studies reported that Dox inhibits the VEGF pathway which is the main anticancer target of the current study via various mechanisms [83,84,85].

**Figure 3:**
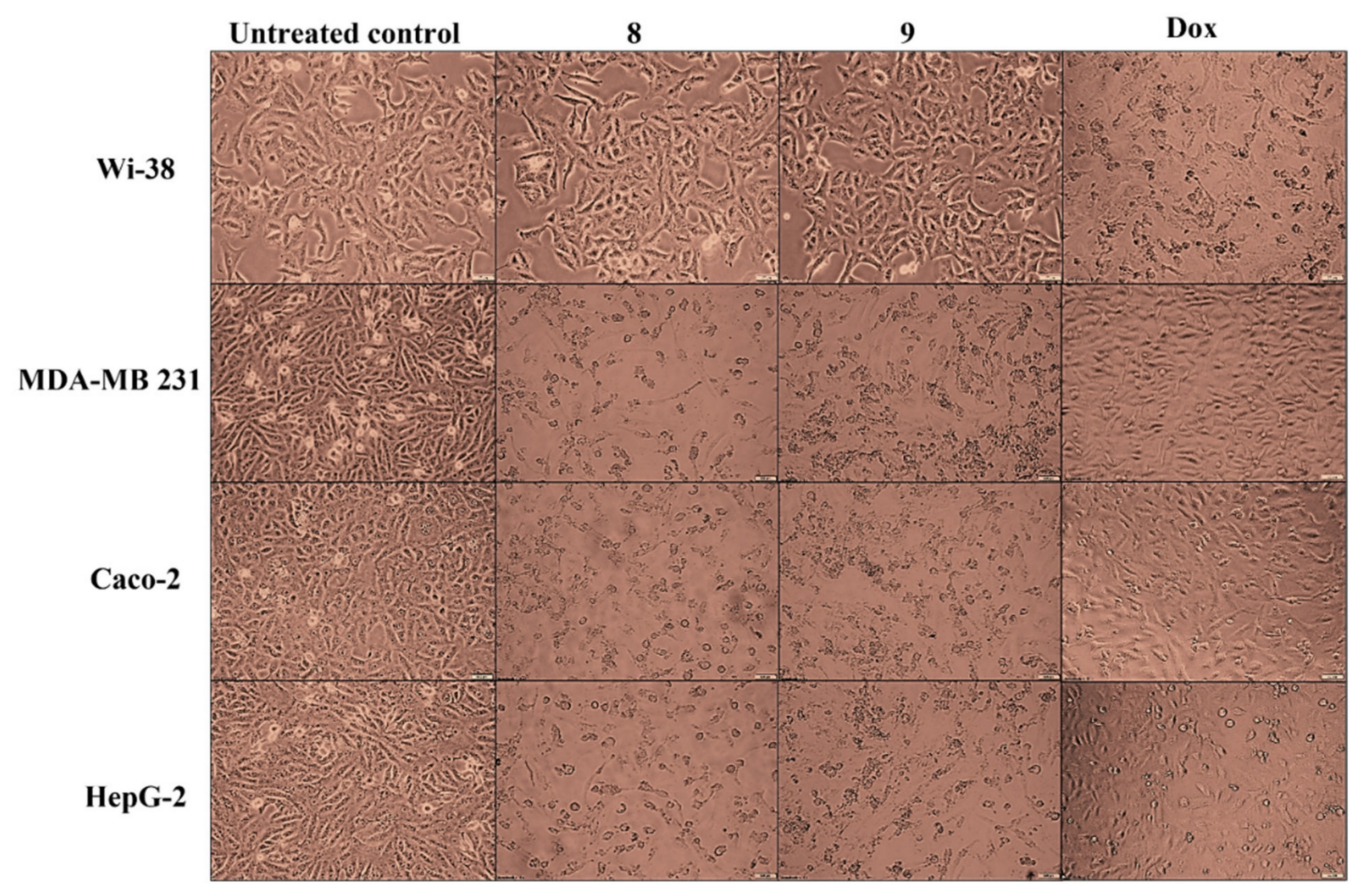
Morphological alteration of **8-** and **9**-treated **Wi-38** cells (at 30 nM), **MDA-MB231**, **Caco-2**, and **HepG-2** (at 1 nM) compared to **Dox-**treated and control cells.

The results (Table 2) revealed that all compounds were safer than Dox (EC_100_ and IC_50_; 11.5 and 30.0 nM) with **7** (EC_100_ and IC_50_; 664.3 and 872.5 nM) at the top of the list, followed by **9, 6,** and **8,** respectively. Among the safest compounds, **8** and **9** displayed outstanding anticancer potencies against the three investigated cell lines, where they recorded single-digit µM IC_50_ values within their safe doses (Table 2). In comparison to Dox, compound **8** was nearly 10 folds superior to Dox against MDA-MB231, 19 folds against Caco-2, and 4 folds against HepG-2 cells. Compound **7** was comparable to Dox against Caco-2 cells but less active against MDA-MB231 and HepG-2 cells. Similarly, **6** was moderately active against the screened cancer cells. The remaining compounds were relatively less active. The activity pattern against MDA-MB231 was more promising than Caco-2 and HepG-2 cells. In other words, it was obvious that MDA-MB231 has higher sensitivity to the evaluated compounds than Caco-2 and HepG-2 cells. The cytotoxicity effect of these active compounds (**8** and **9**), as well as Dox on all studied cell lines, was dose-dependent (Supplementary Figure S40). Figure 3 shows that 1 nM of **8** and **9** caused morphological changes in the treated human cancer cell lines displayed as severe shrinkage [86,87] without causing alterations in the treated normal cells at 30 nM. In contrast to these two compounds, Dox caused morphological damage to the treated Wi-38 cells at concentration (30 nM) lower than its IC_50_ on MDA-MB 231 cells, indicating its low selectivity against cancer cells. Regarding selectivity of the most active derivatives, **8** and **9** exhibited high selectivity index (SI) against MDA-MB 231, Caco-2, and HepG-2 (293.41 and 198.71, 127.83 and 170.24, and 69.22 and 75.81, respectively), whereas Dox had the lowest SI (2.83, 0.62, and 1.33, respectively). Accordingly, these two compounds were raised for further mechanistic studies.

#### 2.2.2. VEGFR-2 Kinase Inhibitory Activity

The in vitro VEGFR-2 kinase inhibitory activity was evaluated for the most active derivatives **8** and **9**. Results revealed remarkable VEGFR-2 inhibitory activities (Table 3, Supplementary Figure S41). Interestingly, **8** was nearly equipotent to sorafenib recording single-digit nanomolar IC_50_ value being ≈2 folds more potent than **9**. Moreover, ligand efficiency metrics were computed [88,89,90] for assessing and prioritizing the drug-likeness of the studied derivatives. Both showed satisfactory LE values (0.26 and 0.25, respectively) compared to the acceptable limit (0.3) [88,89], drug-like LLE values [88,90], and the LELP values [88].

#### 2.2.3. MMP-2 Inhibitory Activity

In vitro MMP-2 inhibitory activities of **8** and **9** were explored in comparison to the reference MMP inhibitor *N*-Isobutyl-*N*-(4-methoxyphenylsulfonyl)glycyl hydroxamic acid (NNGH) [91] (Table 4, Supplementary Figure S42). Both compounds recorded nanomolar IC_50_ and were superior to the reference inhibitor. Compound **8** was 1.77 folds more potent than **9**. MMP-2 inhibitory activity of **8** and **9** echoed their respective anticancer pattern. In addition, the ligand efficiency metrics for both derivatives were drug-like.

#### 2.2.4. CA II Inhibitory Activity

As illustrated (Table 5, Supplementary Figure S43), the examined **8** and **9** displayed submicromolar IC_50_. Again, Compound **8** was superior to **9**. However, the recorded activity pattern may be considered weak when compared to the reference inhibitor [92,93]. The ligand efficiency metrics were also calculated where LE values were approaching acceptable values, while LLE and LELP were optimal and drug-like.

## 3. Structure-Activity Relationship (SAR) and Molecular Modeling Studies

### 3.1. SAR

The general cytotoxicity pattern (Table 2) reflects the effect of cyclizing the D-aldose-(6-methyl-4-oxo-2-pyrimidinyl)hydrazones **2**–**5** on their anticancer potential regarding activity and selectivity. Obviously, all the triazolopyrimidinone acyclo C-nucleosides were superior to their precursor hydrazones. The anticancer profile was found to be a function of the heterocyclic core arrangement, yet the installed sugar part allowed fine-tuning of activity. Within the hydrazones series **2**–**5**, the D-glucose derivative **3** imparted the highest potency against MDA-MB231 and Caco-2 cells, followed by the galactose **5**, mannose **4**, and xylose **2** derivatives. HepG-2 cells were not generally sensitive to the tested hydrazones. Among the acyclo C-nucleoside series, D-glucose conferred the highest potency to the acyclo C-nucleoside **8** against the screened cancer cell lines within the safe dose (EC_100_; Table 2) on normal fibroblasts cells. Replacing glucose **8** by its epimer mannose **9** enhanced the compound’s selectivity and conserved its anticancer potency despite the detected supplemental decrease in IC_50_ values. On the other hand, galactose **7** or xylose **6** substitutions caused observable decrease in activity against MDA-MB231 and Caco-2 and relatively abolished activity against HepG-2 cells.

The SAR was based on the enzymatic assay results of the most promising anticancer triazolopyrimidinone acyclo C-nucleosides **8** and **9** and is better discussed aided by molecular docking simulations, especially considering the fact that the regioisomers **8A** and **8B** separation trials were unsuccessful as that evidenced by the NMR experiments, even under precautions, due to the rapid transformation. Although the other epimeric acyclo C-nucleoside **9** was purified as the single 5-methyl-7-oxo-1,2,4-triazolopyrimidine regioisomer **9A** rather than the 7-methyl-5-oxo-triazolopyrimidine isomer **9B**, docking studies were extended to predict the effect of the trace isomer **9B** (if any) as µ.

### 3.2. Docking Simulations

MOE 2015.10 [94] was employed to perform docking simulations of the hit derivatives into the active sites of the studied enzymes. The study aimed to enrich the SAR, predict the binding modes and key interactions of the active compounds with the active sites, and possibly justify the adopted design rationale. Within this approach, special interest was focused on predicting the structural determinants of activity in the case of the inseparable regioisomers (e.g., **8A** and **8B**), thus identifying which regioisomer might contribute to the inhibitory potential against the studied enzymes.

The studied enzymes’ coordinates were retrieved from the protein databank. Unnecessary residues, ligands, and solvent molecules were eliminated. The protein structures were prepared according to the “QuickPrep” module with MOE default settings. Structures of the studied derivatives **8A**, **8B**, **9A**, and **9B** were built in silico, energy minimized following default geometry optimization settings. Docking was conducted employing various protocols in order to record the best scores and interactions. The docking protocol was validated by re-docking the co-crystallized ligand into the active site. This step reproduced most of the experimental key interactions at acceptable RMSD.

#### 3.2.1. Docking into VEGFR-2 Active Site

**Figure 4:**
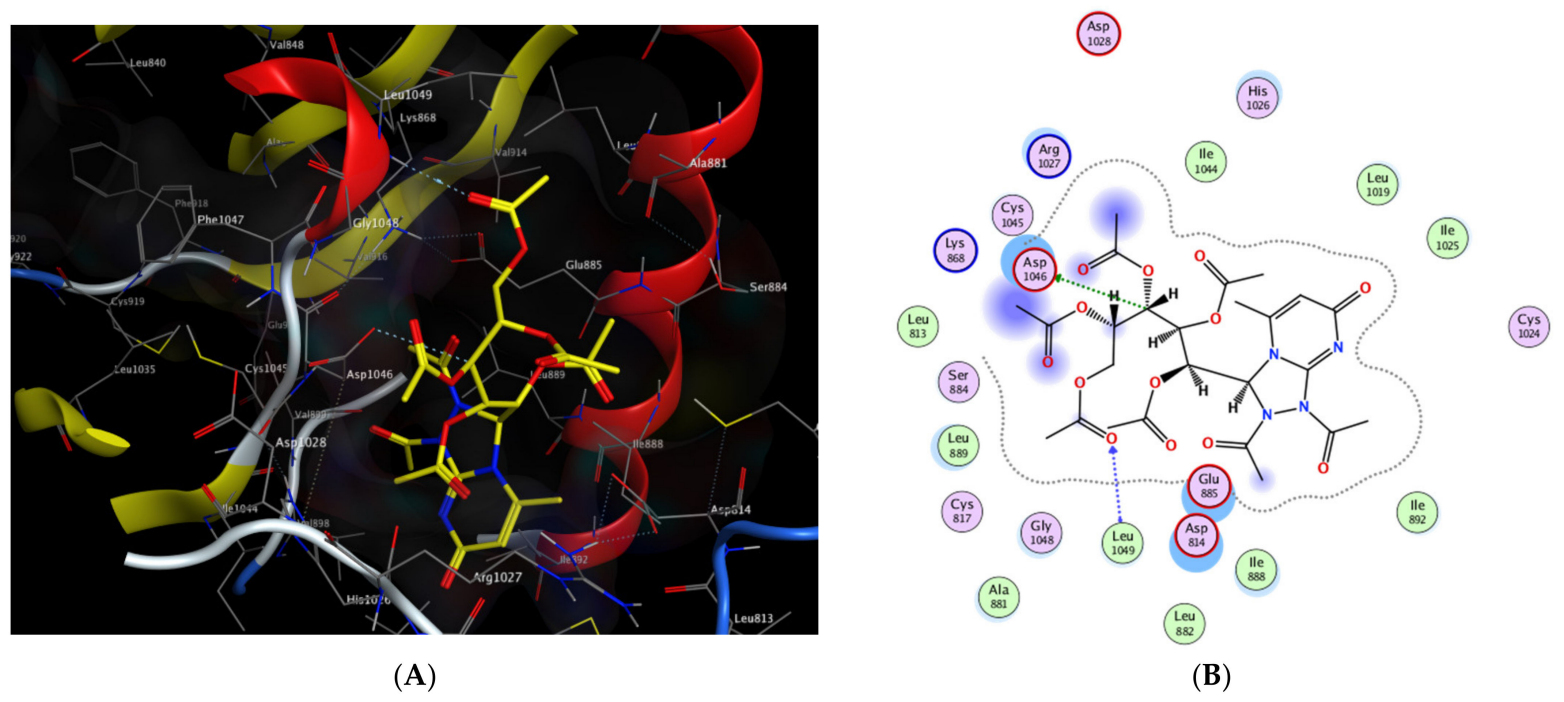

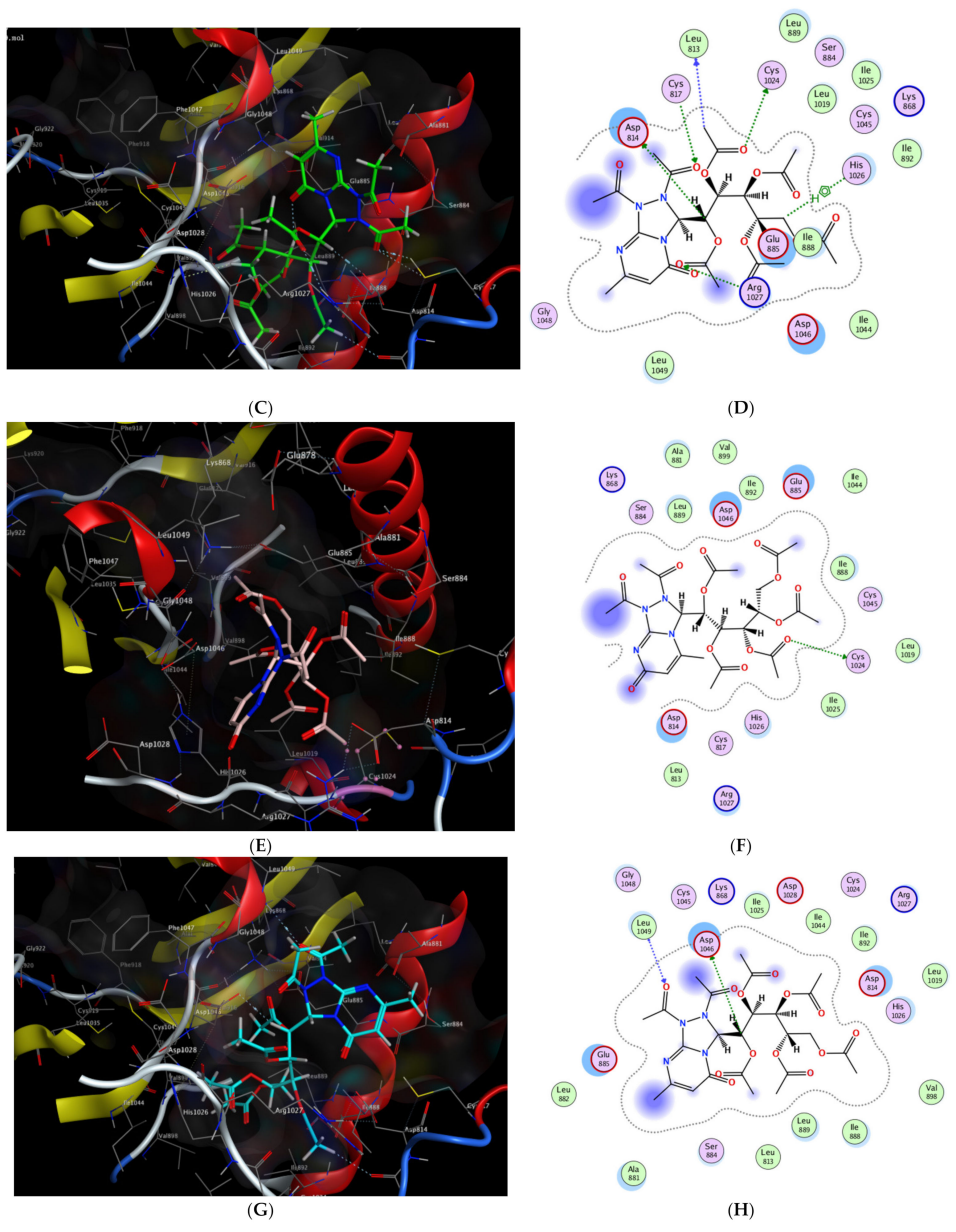

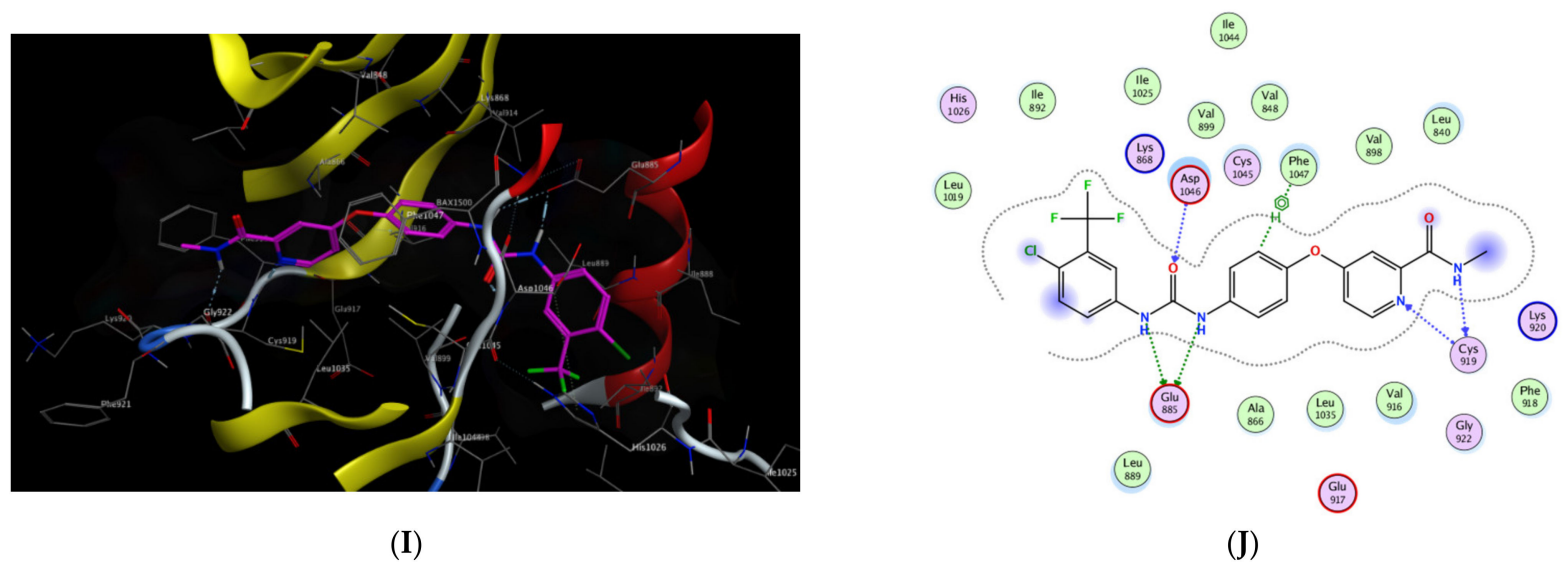
(**A**) The 3D binding mode of **8A** (yellow sticks), (**B**) 2D binding mode of **8A**, (**C**) 3D binding mode of **8B** (green sticks), (**D**) 2D binding mode of **8B**, (**E**) 3D binding mode of **9A** (pink sticks), (**F**) 2D binding mode of **9A**, (**G**) 3D binding mode of **9B** (cyan sticks), (**H**) 2D binding mode of **9B**, (**I**) 3D binding mode of sorafenib (magenta sticks), and (**J**) 2D binding mode of sorafenib in the active site of VEGFR-2 (PDB ID: 4ASD [95]).

VEGFR-2 exists in two different conformations: the active DFG-in and the inactive DFG-out conformations [95]. These conformations are dependent on the DFG motif (activation loop) movement. The DFG-in (active) conformation allows ATP-binding to the active site being accessible, whereas the shift to the DFG-out (inactive) conformation by the movement of the DFG motif discloses an extra hydrophobic pocket adjacent to the binding site. Accordingly, VEGFR-2 inhibitors can be classified into type-I inhibitors competing with ATP for binding to the active conformation [96] and type-II inhibitors stabilizing the inactive conformation. Type-II inhibitors provide superior kinetic advantages over type-I inhibitors by avoiding competition with ATP. In addition, they stabilize VEGFR-2 in the DFG-out inactive conformation [97]. Although type-II inhibitors are highly diverse in structure, some common pharmacophoric features were deduced. These include a flat aromatic system of the main scaffold adopting the active site and an additional moiety that extends into the nearby allosteric site disclosed after DFG motif movement in the inactive conformation [98], typically forming hydrogen bonds with the C-helix Glu885 and the DFG motif Asp1046 [99]. Herein, the designed triazolopyrimidinone acyclo C-nucleosides may be viewed as potential type-II inhibitors based on nearly mimicking their general thematic features regarding the flat aromatic ring system (trizaolopyrimidinone core) and the extended moiety (acetylated sugar part). Therefore, the VEGFR-2 crystal structure (PDB ID: 4ASD) [95] in the “DFG-out” conformation was retrieved from the protein data bank co-crystallized with the type-II inhibitor sorafenib (Figure 4) and utilized for docking simulations of the studied compounds. The docking protocol was conducted as reported [100], employing the default MOE settings, Triangle Matcher placement method, and London dG scoring function. The adopted protocol was validated by docking sorafenib in the VEGFR-2 active site, where the experimental interactions of the co-crystallized ligand were reproduced at acceptable RMSD. Sorafenib displays three hydrogen bonds with Asp1046, involved in the DFG loop move, and Glu885 in the side pocket through its urea motif. The flat heterocyclic ring with a hydrogen bond acceptor forms an essential hydrogen bond with Cys919, while the central phenyl ring is located in the proper position to pose π-π interactions with Phe1047 (Figure 4).

The lowest energy conformer of **8A** posed hydrogen bonding interactions with the active site DFG motif Asp1046 (as sorafenib) as well as Leu1049 via the acylated sugar part carbon skeleton and the terminal acetyl group, respectively. For its regioisomer **8B,** the docking simulations of its low energy conformer displayed hydrogen bonding interactions with Cys817, Arg1027, and Cys1024 near the DFG motif through the carbonyl oxygens of the *N^2^*-acetyl group, the heterocyclic core, and the sugar part acetyl group, respectively. These interactions assisted in positioning the compound properly in the protein active site. It is worth mentioning that the active conformation integrity of VEGFR-2 is dependent on free Cys1024. The hydrogen bonding interactions donated by the acetylated sugar part anomeric CH and acetyl group to the residues Leu813 and Asp814 also firmly clamped the ligand. A further contribution of the acetylated sugar part was observed where **8B** was rendered by H-π with His1046. Therefore, it could be postulated that the 7-methyl-5-oxo-1,2,4 triazolopyrimidine **8B** possessed higher inhibitory potential that its regioisomer **8A**

On the other hand, **9A** and its minor regioisomer **9B** missed most of the previously mentioned interactions. Only one hydrogen bond was displayed by the acetylated sugar part of **9A** with the receptor’s Cys1024, whereas two hydrogen bonds were predicted involving the acetylated sugar part anomeric CH and the key amino acid Asp1046 as well as Leu1049 and *N^1^*-acetyl group in the case of **9B**. These observations postulated that both **8** and **9** may be type-II inhibitors. In addition, the docking simulations results are generally consistent with the in vitro enzymatic activities where **8** was 2 folds more active than **9**.

#### 3.2.2. Docking into MMP-2 Active Site

**Figure 5:**
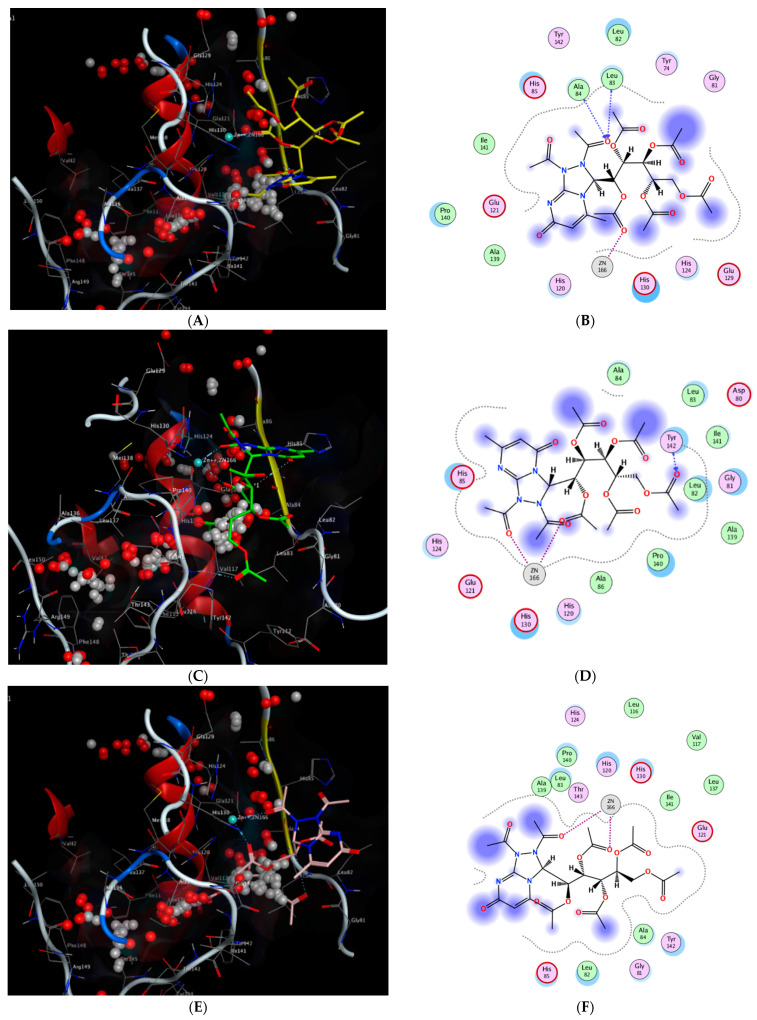

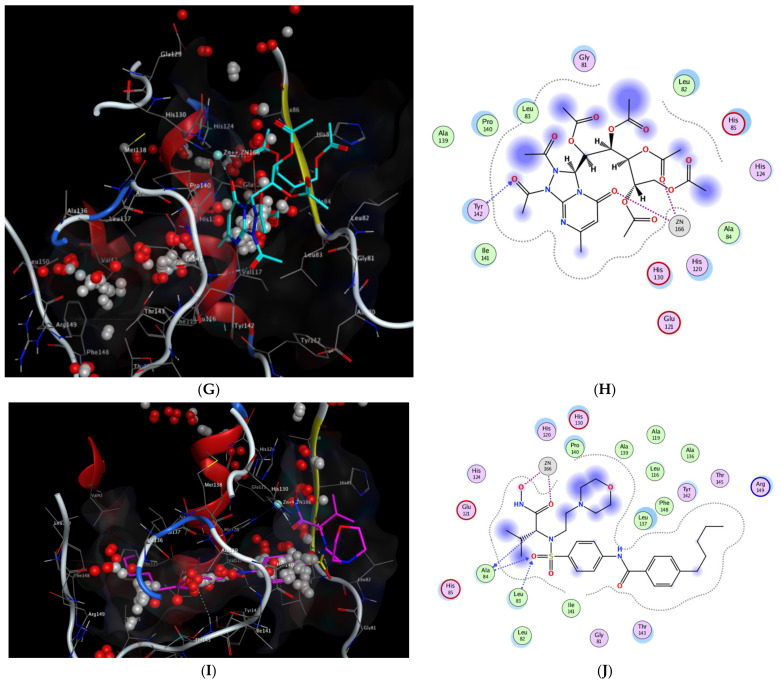
(**A**) The 3D binding mode of **8A** (yellow sticks), (**B**) 2D binding mode of **8A**, (**C**) 3D binding mode of **8B** (green sticks), (**D**) 2D binding mode of **8B**, (**E**) 3D binding mode of **9A** (pink sticks), (**F**) 2D binding mode of **9A**, (**G**) 3D binding mode of **9B** (cyan sticks), (**H**) 2D binding mode of **9B,** (**I**) 3D binding mode of the co-crystallized the hydroxamic acid inhibitor SC-74020 (magenta sticks), and (**J**) 2D binding mode of SC-74020 in the active site of MMP-2 (PDB ID: 1HOV [101]).

After employing the default protein preparation procedure, the MMP-2 (PDB ID: 1HOV) active site [101] was located via the “Site Finder” feature of MOE 2015.10 to avoid any bias to the co-crystallized hydroxamic acid ligand SC-74020. As illustrated (Figure 5), SC-74020 chelates the active site zinc ion through its hydroxamate moiety and displays hydrogen bond interactions with Leu83 and Ala84. Herein, the best binding modes of the studied derivatives (Figure 5) were computed employing rigid docking utilizing the Triangle Matcher placement method and London dG scoring function. The low-energy conformer of **8A** displayed hydrogen bonding interactions with the ligand essential key amino acid residues Leu83 and Ala84 via the core *N^2^*-acetyl group, whereas the sugar acetyl group was oriented to bind the active site zinc. Interestingly, its regioisomer **8B** chelated the active site zinc ion resembling the binding mode of classical potent zinc-chelating MMP inhibitors. Such interactions were posed by the *N^1^*-acetyl and nearby sugar acetyl groups. It is also obvious that the orientation of **8B** allowed the proximity of the acetylated sugar part to the selectivity S1′ pocket and formation of the hydrogen bond interaction with Tyr142. On the other hand, the regioisomer **9A** was predicted to chelate only with the active site zinc via the core *N^2^*-acetyl and the sugar part acetyl groups without further interactions with the active site key amino acid residues. Its isomer **9B** acquired a slightly bent conformation to coordinate the zinc ion via the sugar part acetyl moiety and the distant triazolopyrimidinone carbonyl group. Hence, its triazolopyrimidinone core was positioned toward the S1′ pocket posing the *N^1^*-acetyl group for hydrogen bonding with the active site Tyr142. Taken together, the 7-methyl-5-oxo-1,2,4 triazolopyrimidine regioisomers (**8B** and **9B**) might have contributed to potency more than their respective isomers **9A** and **9B**. From another point of view, these results may provide a reasonable explanation for the better accommodation of **8B** in the active site compared to **9B** as evinced by their recorded binding scores (**8B**; ΔG = 8.81 Kcal/mol) and (**9B**; ΔG = 8.03 Kcal/mol). Results nearly resembled the in vitro inhibition profiles.

#### 3.2.3. Docking into CA II Active Site

The X-ray crystal structure of CA II (PDB ID:1BN1) [102] complexed with 4-Aminobenzenesulfonamide was downloaded from the protein data bank. The enzyme–ligand complex was unmerged for achieving the free enzyme, then the possible binding sites were located via the MOE “Site Finder” feature considering the key residues and ions (i.e., zinc). This feature is generally employed for avoiding the bias to the metal-binding mode of the co-crystallized ligand [103] or in the case of its absence [104]. Docking validation was performed by redocking the co-crystallized inhibitor in the selected binding site and restoring most of the experimental key interactions (zinc-binding and hydrogen bonding interactions with the key residues Gln92 and Thr199).

**Figure 6:**
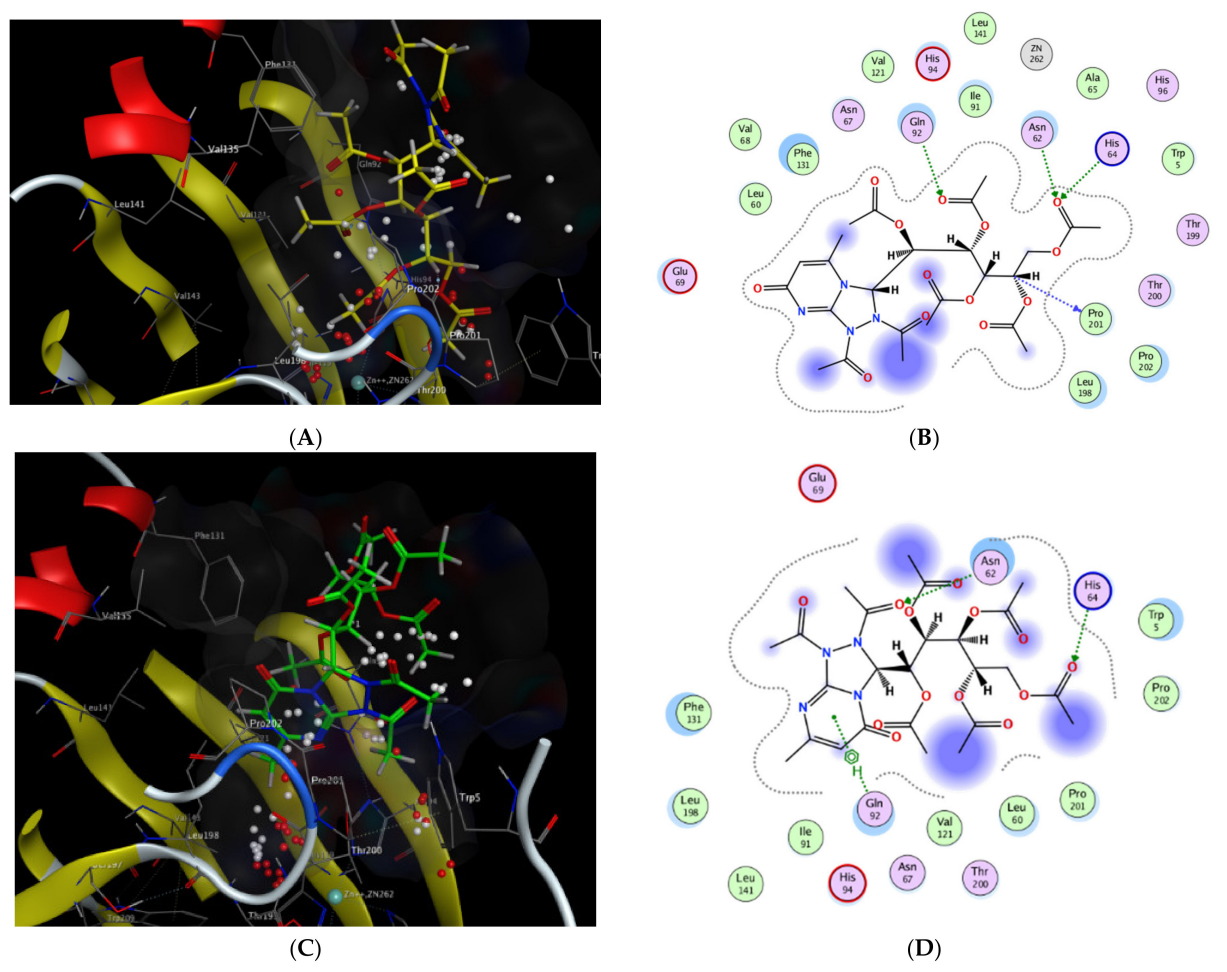

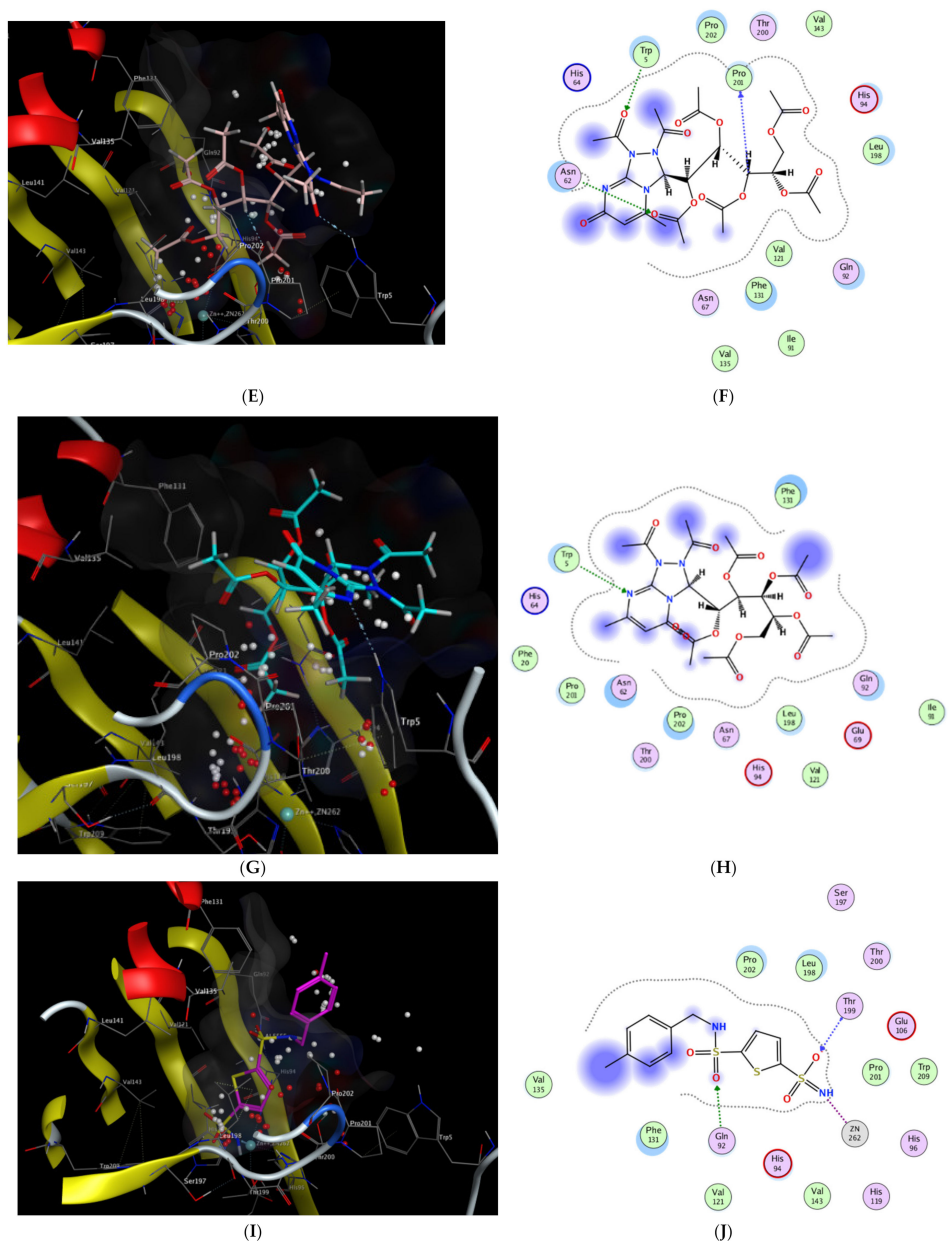
(**A**) The 3D binding mode of **8A** (yellow sticks), (**B**) 2D binding mode of **8A**, (**C**) 3D binding mode of **8B** (green sticks), (**D**) 2D binding mode of **8B**, (**E**) 3D binding mode of **9A** (pink sticks), (**F**) 2D binding mode of **9A**, (**G**) 3D binding mode of **9B** (cyan sticks), (**H**) 2D binding mode of **9B**, (**I**) 3D binding mode of the co-crystallized CA II inhibitor **AL5** (magenta sticks), and (**J**) 2D binding mode of **AL5** in the active site of CA II (PDB ID:1BN1 [102]).

The best binding modes of the studied derivatives (Figure 6) were computed employing the validated protocol, namely rigid docking utilizing the Triangle Matcher placement method and London dG scoring function. Docking simulations predicted interactions of the regioisomers **8A** and **8B** with the active site Gln92, ASn62, and His64. As seen (Figure 6), the acylated sugar part of **8A** accepted a key hydrogen bond from Gln92 resembling the co-crystallized inhibitor binding mode as well as additional hydrogen bonds with ASn62, His64, and Pro201. In the case of **8B**, H-π interactions were recorded between the heterocyclic core of **8B** and the ligand essential amino acid Gln92. In addition, hydrogen bonding interactions posed by *N*^2^-acetyl and the acetylated sugar part to ASn62 and His64, respectively, allowed more fitting of the compound at the active site. The isomer **9A** interacted with the active site through hydrogen bonding with ASn62, Pro201, and Trp5. For **9B**, most of the key interactions were missed where only hydrogen bonding between the pyrimidine nitrogen and Trp5 was predicted. These results were nearly related to the relatively weak in vitro inhibition profiles of **8** and **9.**

## 4. Experimental Section

### 4.1. Chemistry

#### 4.1.1. General Methods and Instruments

All melting points were determined using Mel-Temp apparatus and are uncorrected. Reactions were monitored by thin-layer chromatography (TLC) carried out on precoated Aluminum plates of silica gel (Kiesel gel G, Merk). Visualization was accomplished with ultraviolet light (UV 254 nm). Sonication was performed using Ultrasonic Cleaner model UD50SH-2LQ. IR spectra were recorded using Bruker Tensor 37 FTIR spectrophotometer. NMR spectra were recorded with Bruker DRX 400 at the Faculty of Pharmacy, Cairo University, Egypt, and JEOL ECA 500, NMR unit, Mansoura University, Egypt. Chemical shifts are reported in parts per million (ppm) and coupling constants *J* are given in Hz (Hertz). Chemical shifts are reported relative to TMS (d = 0.0) as an internal standard. (Abbreviations used in spectra: s = singlet, d = doublet, t = triplet, q = quartet, m = multiple, bs = broad singlet, dd = double of doublets). Microanalysis was performed at the Faculty of Science, El Azhar University, Egypt, and Konstanz University, Germany.

#### 4.1.2. Synthesis of *D*-Aldose-(6-methyl-4-oxo-2-pyrimidinyl)hydrazones **2**–**5**

A mixture of 2-hydrazinyl-6-methylpyrimidin-4-one (1 mmol) **1** and aldose (1 mmol) in ethanol (25mL) was refluxed in a water bath for 7-10 h or under ultrasound irradiation at 70–75 °C for 45–120 min. The reaction mixture was then concentrated then left to cool, filtered, washed with water, and dried, affording the respective sugar hydrazones **2**, **3**, **4,** or **5**.

##### *D*-Xylose-(6-methyl-4-oxo-2-pyrimidinyl)hydrazone **2**

Recrystallized from absolute ethanol, yield (92%), m. p. 215–216 °C (Lit. [64] m. p. 220 °C). IR (KBr) ν (cm^−1^): 3424 (NH), 1659 (CONH), 1611 (CH=N). ^1^H-NMR (DMSO-*d_6_*, 400 MHz) δ_H_ ppm; 2.03, 2.05 (2 s, 3 H, CH_3_ **A** and **B**), 2.98–3.06 (m, 0.5 H, H-2′, H-5′ **B**), 3.11-3.16 (m, 0.25 H, H-3′ **B**), 3.22–3.27 (m, 0.25 H, H-4′ **B**), 3.38 (d, 0.75 H, *J*_5′,4′_ = 5.6 Hz, H-5′ **A**), 3.45-3.48 (m, 0.75 H, H-5′’ **A**), 3.52–3.54 (m, 1.5 H, H-3′ **A**, H-4′ **A**), 3.67, 3.70 (t, d, 0.5 H, H-5′’, H-1′ **B**, *J*_5′’,4′_ = 5.6 Hz, *J*_1′,2′_ = 8.8 Hz), 4.25 (q, 0.75 H, H-2′ **A**), 4.40 (d, 0.75 H, D_2_O exchangeable, OH **A**), 4.45 (t, 0.75 H, D_2_O exchangeable, OH **A**), 4.56 (d, 0.75 H, D_2_O exchangeable, OH **A**), 4.96 (t, 0.5 H, D_2_O exchangeable, 2 X OH **B**), 5.26 (d, 0.75 H, D_2_O exchangeable, OH-2′ **A**), 5.37 (bs, 0.25 H, D_2_O exchangeable, OH **B**), 5.42 (s, 0.25 H, H-5 pyrimidine **B**), 5.50 (s, 0.75 H, H-5 pyrimidine **A**), 5.76 (d, 0.25 H, D_2_O exchangeable, NH **B**), 7.49 (d, 0.75 H, *J* = 3.2 Hz, CH=N), 8.70, 10.36 (2 bs, 0.5 H, D_2_O exchangeable, 2 X NH **B**), 11.06, 11.31 (2 bs, 1.5 H, D_2_O exchangeable, 2 X NH **A**). ^13^C-NMR (DMSO-*d_6_*, 100 MHz) δ_C_ ppm; 23.42, 23.44 (CH_3_ **A** and **B**), 63.0 (C-5′ **A**), 67.4 (C-5′ **B**), 70.0 (C-4′ **B**), 71.4 (C-2′ **B**), 71.6 (C-4′ **A**), 72.1 (C-2′ **A**), 72.5 (C-3′ **A**), 77.5 (C-3′ **B**), 92.2 (C-1′ **B**), 101.6, 101.7 (C-5 pyrimidine **A** and **B**), 148.5 (CH=N), 153.0 (C-6), 156.9 (C-2), 163.0 (C-4). Anal. Cal. For C_10_H_16_N_4_O_5_: C, 44.12, H, 5.92, N, 20.58. Found: C, 44.35, H, 6.14, N, 20.19.

##### *D*-Glucose-(6-methyl-4-oxo-2-pyrimidinyl)hydrazone **3**

Recrystallized from ethanol/ water, yield (89%), m. p. 240–242 °C (Lit. [64] m. p. 260 °C). IR (KBr) ν (cm^−1^): 3211 (NH), 1667 (CONH), 1616 (CH=N). ^1^H-NMR (DMSO-*d_6_*, 400 MHz) δ_H_ ppm; 2.02 (s, 2.58 H, CH_3_ **B**), 2.05 (s, 0.42 H, CH_3_ **A**), 2.90-2.96 (td, 0.86 H, *J*_4′,3′_ = 8.8 Hz, H-4′**B**), 3.02 (t, 0.86 H, *J*_2′,3′_ = 8.8 Hz, H-2′**B**), 3.11-3.15 (m, 0.86 H, H-5′ **B**), 3.16-3.21 (td, 0.86 H, H-3′ **B**), 3.36-3.38 (m, 0.86 H, H-6′’ **B**), 3.40-3.43 (m, 0.28 H, H-4′ **A** and H-6′’ **A**), 3.46-3.50 (m, 0.14 H, H-5′ **A**), 3.56-3.58 (m, 0.14 H, H-6′ **A**), 3.66 (t, 0.86 H, *J*_6′,6′’_ = 11.6 Hz, H-6′ **B**), 3.72 (t, 0.86 H, *J*_1′,2′_ = 9.2 Hz, H-1′ **B**), 3.78 (bd, 0.14 H, H-3′ **A**), 4.25 (q, 0.14 H, H-2′ **A**), 4.30 (t, 0.14 H, D_2_O exchangeable, OH **A**), 4.48 (d, 0.14 H, D_2_O exchangeable, OH **A**), 4.51-4.54 (dd, 0.86 H, D_2_O exchangeable, OH-6′ **B**), 4.57 (d, 0.14 H, D_2_O exchangeable, OH **A**), 4.94, 4.98 (2 d, 1.72 H, D_2_O exchangeable OH-3′ **B** and OH-4′ **B**), 5.31 (bs, 0.86 H, D_2_O exchangeable, OH-2′ **B**), 5.42 (s, 0.86 H, H-5 pyrimidine **B**), 5.49 (s, 0.14 H, H-5 pyrimidine **A**), 5.75 (d, 0.86 H, D_2_O exchangeable, *J* = 10.0 Hz, NH **B**), 7.49 (d, 0.14 H, *J* = 3.6 Hz, CH=N), 8.92 (bs, 0.86 H, D_2_O exchangeable, NH **B**), 10.66 (s, 0.86, D_2_O exchangeable, NH **B**), 11.05, 11.39 (2 bs, 0.28 H, D_2_O exchangeable, 2 X NH **A**). ^13^C-NMR (DMSO-*d_6_*, 100 MHz) δ_C_ ppm; 23.9 (CH_3_ **A**), 62.8 (C-6′ **B**), 63.8 (C-6′ **A**), 71.1 (C-4′ **B**), 71.6 (C-3′ **A**), 71.7 (C-5′ **A**), 71.8 (C-2′ **B**), 72.8 (C-2′ **A**), 77.4 (C-3′ **B**), 78.1 (C-5′ **B**), 91.3 (C-1′ **B**), 100.8 (C-5 pyrimidine), 156.6 (C-6), 163.0 (C-2), 166.5 (C-4). Anal. Cal. For C_11_H_18_N_4_O_6_: C, 43.71, H, 6.00, N, 18.53. Found: C, 43.52, H, 6.13, N, 18.27.

##### *D*-Mannose-(6-methyl-4-oxo-2-pyrimidinyl)hydrazone **4**

Recrystallized from absolute ethanol, yield (88%), m. p. 220–222 °C (Lit. [64] m. p. 180 °C). IR (KBr) ν (cm^−1^): 3313 (NH), 1674 (CONH), 1639 (CH=N). ^1^H-NMR (DMSO-*d_6_*, 400 MHz) δ_H_ ppm; 2.01 (s, 0.42 H, CH_3_ **B**), 2.03, 2.05 (2s, 2.58 H, CH_3_ **A**), 3.06 (t, 0.14 H, H-5′ **B**, *J*_5′,4′_ = 8.4 Hz), 3.18-3.23 (pentet, 0.14 H, H-4′ **B**), 3.26-3.28 (m, 0.14 H, H-3′ **B**), 3.40-3.44 (m, 1.14 H, H-6′ **A,** H-6′ **B** and H-6′’ **B**), 3.47–3.48 (m, 0.86 H, H-5′ **A**), 3.54–3.62 (m, 1.72 H, H-3′ **A** and H-4′ **A**), 3.65-3.70 (m, 0.86 H, H-6′’ **A**), 3.87 (bs, 0.14 H, H-2′ **B**), 4.01 (d, 0.14 H, H-1′, *J*_1′,NH_ = 10.8 Hz), 4.13–4.16 (m, 0.86 H, H-2′ **A**), 4.35–4.39 (m, 1.72 H, D_2_O exchangeable, OH-5′ and OH-6′ **A**), 4.44–4.46 (m, 1.72 H, D_2_O exchangeable, OH-3′ and OH-4′ **A**), 4.75 (m, 0.42 H, D_2_O exchangeable, OH **B**), 5.20 (d, 1 H, D_2_O exchangeable, OH-2′ **A**, NH **B**), 5.42 (s, 0.14 H, H-5 pyrimidine **B**), 5.50 (s, 0.86 H, H-5 pyrimidine **A**), 7.66 (d, 0.86 H, *J* = 2.4 Hz, CH=N), 8.78, 10.07 (2 bs, 0.28 H, D_2_O exchangeable, 2 X NH **B**), 11.12 (bs, 1.72 H, D_2_O exchangeable, 2 X NH **A**). ^13^C-NMR (DMSO-*d_6_*, 100 MHz) δ_C_ ppm; 23.45 (CH_3_ **A**), 63.0 (C-6′ **B**), 64.2 (C-6′ **A**), 68.2 (C-4′ **B**), 69.90 (C-2′ **B**), 69.98 (C-4′ **A**) 70.2 (C-2′ **A**), 71.4 (C-5′ **A**), 72.5 (C-3′ **A**), 74.4 (C-3′ **B**), 78.5 (C-5′ **B**), 88.1 (C-1′ **B**), 101.7 (C-5 pyrimidine **A**), 149.9 (CH=N), 153.1 (C-6 **A**), 163.1 (C-4 **A**), 165.0 (C-4 **B**). Anal. Cal. For C_11_H_18_N_4_O_6_: C, 43.71, H, 6.00, N, 18.53. Found: C, 43.98, H, 6.23, N, 18.70.

##### *D*-Galactose-(6-methyl-4-oxo-2-pyrimidinyl)hydrazone **5**

Recrystallized from ethanol/water, yield (93%), m. p. 213–214°C (Lit. [64] m. p. 250 °C). IR (KBr) ν (cm^−1^): 3379 (NH), 1660 (CONH), 1611 (CH=N). ^1^H-NMR (DMSO-*d_6_*, DMSO-*d_6_* + D_2_O, 400 MHz) δ_H_ ppm; 2.01, 2.03, 2.05 (3 s, 3 H, CH_3_ **A** and **B**), 3.31 (t, 0.65 H, H-2′ **B**, *J*_2′,1′_ = 8.4 Hz, *J*_2′,3′_ = 9.2 Hz), 3.36 (d, 0.65 H, *J*_4′,5′_ = 2.8 Hz, H-4′ **B**), 3.39 (bs, 0.35 H, H-4′ **A**), 3.40-3.44 (m, 1.6 H, H-6′ **A**, 6′’ **A** and H-6′ **B**), 3.53-3.57 (m, 1 H, H-3′ **A** and H-3 ’ **B**), 3.59 (bd, 0.40 H, H-6′’ **B**), 3.66 (t, 0.3 H, H-1′ α **B**, *J*_1′,2′_ = 8.4 Hz), 3.71-3.76 (m, 1 H, H-5′ **A** and H-5′ **B**), 4.14-4.18 (m, 1.4 H, D_2_O exchangeable, 3 X OH), 4.34 (d, 0.10 H, H-1′ β **B**, *J*_1′,2′_ = 4.0 Hz), 4.42-4.47 (m, 2.1 H, H-2′ **A** and D_2_O exchangeable, OH’s), 4.78 (bt, 0.27 H, D_2_O exchangeable, OH), 5.01 (d, 0.6 H, D_2_O exchangeable, OH-2′ **A**), 5.21–5.25 (m, 0.4 H, D_2_O exchangeable, OH), 5.41, 5.43 (2 s, 0.40 H, H-5 pyrimidine **B**), 5.49 (s, 0.6 H, H-5 pyrimidine **A**), 5.71 (d, 0.3 H, *J*_NH, 1′α_ = 6.0 Hz, D_2_O exchangeable, NH **B**), 5.79 (d, 0.1 H, *J*_NH, 1′β_ = 9.6 Hz, D_2_O exchangeable, NH **B**), 7.45 (d, 0.6 H, *J* = 3.2 Hz, CH=N), 8.82, 10.60 (2 bs, 0.5 H, D_2_O exchangeable, 2 X NH **B**), 11.06 (bs, 1.2 H, D_2_O exchangeable, 2 X NH **A**). ^13^C-NMR (DMSO-*d_6_*, 100 MHz) δ_C_ ppm; 23.4 (CH_3_ **A**) 23.9 (CH_3_ **B**), 61.7 (C-6′ **B**), 63.5 (C-6′ **A**), 68.9 (C-4′ **A**), 69.5 (C-3′ **A**), 70.3 (C-5′ **A** and **B**), 70.4 (C-2′ **A**), 71.2 (C-3′ **B**), 74.1 (C-2′ **B**), 76.9 (C-4′ **B**), 92.0 (C-1′ α **B**), 94.5 (C-1′ β **B**), 101.6, 101.7 (C-5 pyrimidine **A** and **B**), 149.6 (CH=N), 153.0 (C-6 **A** and **B**), 156.7, 156.9 (C-2 **A** and **B**), 163.0, 163.1 (C-4 **A** and **B**). Anal. Cal. For C_11_H_18_N_4_O_6_: C, 43.71, H, 6.00, N, 18.53. Found: C, 43.98, H, 5.96, N, 18.72.

#### 4.1.3. Synthesis of *N*^1^, *N*^2^-Diacetyl-3-(per-*O*-acetylated sugar-alditol-1-yl)-5(7)-methyl-7(5)-oxo-1,2,4-triazolo[4,3-*a*]pyrimidine **6**–**9**

Compound **2**–**5** (1 mmol) was heated with acetic anhydride (5 mL) under water bath for 1–3 h. Then, reaction mixture was poured onto crushed ice, extracted with dichloromethane (3 × 10 mL), washed with saturated NaHCO_3_, watered and dried (Na_2_SO_4_). The extract was evaporated under diminished pressure and purified by column chromatography on silica gel using ethyl acetate to give 1,2,4-triazolo[4,3-*a*]pyrimidines **6**–**9**.

##### *N*^1^,*N*^2^-Diacetyl-3-(1,2,3,4-tetra-*O*-acetyl-*D*-xylo-tetritol-1-yl)-7-methyl-5-oxo-1,2,4-triazolo[4,3-*a*]pyrimidine **6**

Compound **6** was obtained as colorless crystals in 32% yield; m. p. 112–114 °C, R_F_ = 0.64 (Ethyl acetate). IR (KBr) υ (cm^−1^): 1748, 1702 (COCH_3_), 1660(CON). ^1^H-NMR (CDCl_3_, 400 MHz) δ_H_ ppm: 2.02, 2.06, 2.11, 2.17, 2.27 (5s, 21 H, CH_3_ (7) and 6 X CH_3_CO), 4.07 (dd, 1 H, H-4′, *J*_4′,4′’_ = 11.6 Hz, *J*_4′,3′_ = 7.2 Hz), 4.24, 4.26 (2d, 1 H, H-4′’, *J*_4′’,3′_ = 4.8 Hz), 5.30 (s, 1 H, H-6), 5.50 (dd, 1 H, H-2′, *J*_2′,1′_ = 8.8 Hz, *J*_2′,3′_ = 2.0 Hz), 5.77 (d, 1 H, H-1′), 6.04-6.07 (m, 1 H, H-3′), 6.44 (s, 1 H, H-3). ^13^C-NMR (CDCl_3_, 100 MHz) δ_C_ ppm: 19.2 (CH_3_ (7)), 20.4, 20.5, 20.60, 20.66, 20.7, 21.0 (6 X CH_3_CO), 62.2 (C-4′), 68.3 (C-3′), 69.7 (C-2′), 70.9 (C-1′), 71.4 (C-3), 98.2 (C-6), 147.4 (C-7), 151.2 (C-8a), 158.8 (C-5), 169.4, 169.9, 170.61, 170.63, 170.8, 175.5 (6 X CH_3_CO). Anal. Calc. for C_22_H_28_N_4_O_11_: C, 50.38, H, 5.38, N, 10.68. Found: C, 50.70, H, 5.49, N, 10.87.

##### *N*^1^,*N*^2^-Diacetyl-3-(1,2,3,4,5-penta-*O*-acetyl-D-galacto-pentitol-1-yl)-7-methyl-5-oxo-1,2,4-triazolo[4,3-*a*]pyrimidine **7**

Compound **7** was obtained as colorless crystals in 50% yield; m. p. 97–100 °C, R_F_ = 0.57 (Ethyl acetate). IR (KBr) υ (cm^−1^): 1750, 1706 (COCH_3_), 1667(CON). ^1^H-NMR (CDCl_3_, 400 MHz) δ_H_ ppm: 2.03, 2.07, 2.11, 2.13 (5bs, 24 H, CH_3_ (7) and 7 X CH_3_CO), 3.89 (bs, 1 H, H-5′), 4.22 (bs, 1 H, H-5′’), 5.17 (bs, 1 H, H-4′), 5.23 (s, 1 H, H-6), 5.39 (d, 1 H, H-2′, *J*_2′,3′_ = 8.4 Hz), 5.42 (bs, 1 H, H-1′), 5.58 (d, 1 H, H-3′, *J*_3′,2′_ = 8.8 Hz), 6.59 (s, 1 H, H-3). ^13^C-NMR (CDCl_3_, 100 MHz) δ_C_ ppm: 19.2 (CH_3_ (7)), 20.5, 20.6, 20.71, 20.79, 20.8, 20.9, 21.0 (7 X CH_3_CO), 61.8 (C-5′), 66.3 (C-3′), 67.6 (C-4′), 68.1 (C-2′), 69.5 (C-1′), 70.0 (C-3), 97.4 (C-6), 158.8 (C-5), 169.8, 170.40, 170.44, 170.5 (7 X CH_3_CO). Anal. Calc. for C_25_H_32_N_4_O_13_: C, 50.33, H, 5.41, N, 9.39. Found: C, 50.46, H, 5.58, N, 9.45.

##### *N*^1^,*N*^2^-Diacetyl-3-(1,2,3,4,5-penta-*O*-acetyl-*D*-gluco-pentitol-1-yl)-5-methyl-7-oxo-1,2,4-triazolo[4,3-*a*]pyrimidine **8A** and *N*^1^, *N*^2^-diacetyl-3-(1,2,3,4,5-penta-*O*-acetyl-*D*-gluco-pentitol-1-yl)-7-methyl-5-oxo-1,2,4-triazolo[4,3-*a*]pyrimidine **8B**

Compound **8A/B** was obtained as colorless crystals in 33% yield; m. p. 92–95 °C, R_F_ = 0.6 (Ethyl acetate/*n*-hexane 4:1). IR (KBr) υ (cm^−1^): 1746, 1696 (COCH_3_), 1668(CON). ^1^H-NMR (CDCl_3_, 400 MHz) δ_H_ ppm: 1.94, 1.99, 2.04, 2.05, 2.09, 2.19, 2.27, 2.32 (8s, 24 H, CH_3_ (5) and 7 X CH_3_CO), 4.08-4.13 (m, 1 H, H-5′, *J*_5′,4′_ = 7.5 Hz), 4.22 (dd, 1 H, H-5′’, *J*_5′’,4′_ = 2.5 Hz, *J*_5′’,5′_ = 12.5 Hz), 5.15 (bs, 2 H, H-4′, H-6 **8B**), 5.44-5.51 (m, 1 H, H-2′, *J*_2′,3′_ = 1.5 Hz, *J*_2′,1′_ = 9.5 Hz), 5.62-5.69 (m, 1 H, H-1′, *J*_1′,2′_ = 9.5 Hz), 5.99 (bs, 1 H, H-6 **8A**), 6.16, 6.23 (2d, 1 H, H-3′, *J*_3′,4′_ = 9.0 Hz), 6.40 (s, 1 H, H-3 **8A**). ^13^C-NMR (CDCl_3_, 100 MHz) δ_C_ ppm: 19.3 (CH_3_ (7)), 20.3, 20.5, 20.6, 20.7, 20.91, 20.93, 21.0 (7 X CH_3_CO), 62.2 (C-5′), 67.6 (C-3′, C-3 **8A**), 68.1 (C-4′), 69.2 (C-2′), 71.3 (C-1′), 71.6 (C-3 **8B**), 98.0 (C-6 **8B**), 108.6 (C-6 **8A**), 146.8 (C-5), 150.7 (C-8a), 157.9 (C-7), 169.2, 169.7, 169.8, 170.2, 170.6, 170.8, 176.3 (7 X CH_3_CO). Anal. Calc. for C_25_H_32_N_4_O_13_: C, 50.33, H, 5.41, N, 9.39. Found: C, 50.47, H, 5.57, N, 9.18.

##### *N*^1^,*N*^2^-Diacetyl-3-(1,2,3,4,5-penta-*O*-acetyl-*D*-manno-pentitol-1-yl)-5-methyl-7-oxo-1,2,4-triazolo[4,3-*a*]pyrimidine **9A** and *N*^1^,*N*^2^-diacetyl-3-(1,2,3,4,5-penta-*O*-acetyl-*D*-manno-pentitol-1-yl)-7-methyl-5-oxo-1,2,4-triazolo[4,3-*a*]pyrimidine **9B**

Compound **9A** was obtained as colorless crystals in 62% yield; m. p. 100–102 °C, R_F_ = 0.70 (Ethyl acetate). IR (KBr) υ (cm^−1^): 1750 (COCH_3_), 1672 (CON). ^1^H-NMR (CDCl_3_, 400 MHz) δ_H_ ppm: 1.90, 2.04, 2.08, 2.12, 2.17, 2.21, 2.31, 2.63 (8s, 24 H, CH_3_ (5) and 7 X CH_3_CO), 4.12 (dd, 1 H, H-5′, *J*_5′,4′_ = 4.8, *J*_5′,5′’_ = 12.8 Hz), 4.26 (dd, 1 H, H-5′’, *J*_5′’,4′_ = 2.5 Hz), 4.96-5.00 (m, 1 H, H-4′), 5.38 (d, 1 H, H-3′, *J*_3′,4′_ = 8.8 Hz), 5.56 (bs, 2 H, H-1′ and H-2′), 6.07 (s, 1 H, H-6), 6.74 (s, 1 H, H-3), ^13^C-NMR (CDCl_3_, 100 MHz) δ_C_ ppm: 20.2, 20.5. 20.61, 20.69, 20.7, 20.9, 22.5, 24.0 (CH_3_ (7) and 7 X CH_3_CO), 61.4 (C-5′), 67.1 (C-2′), 67.5 (C-3′), 68.11 (C-3), 68.17 (C-1′), 68.3 (C-4′), 109.1 (C-6), 158.0 (C-7), 168.5, 169.5, 169.8, 170.0, 170.1, 170.5, 175.1 (7 X CH_3_CO). Exposure of **9A** to light for 63 h afforded an equimolar mixture of **9A**: **9B** (1:1) as that observed from its NMR spectra; ^1^H-NMR (CDCl_3_, 400 MHz) δ_H_ ppm: 1.89, 2.05, 2.08, 2.11, 2.12, 2.13, 2.18, 2.21, 2.31, 2.63 (10 s, 48 H, 2 X CH_3_ and 14 X CH_3_CO, **9A** and **9B**), 4.10, 4.13 (dd, 1 H, H-5′, **9A** and **9B**), 4.23–4.26 (m, 1 H, H-5′’ **9A** and **9B**), 4.96–5.00 (m, 0.5 H, H-4′, **9A**), 5.05-5.08 (m, 0.5 H, H-4′ **9B**), 5.26 (d, 0.5 H, H-2′, *J*_2′,1′_ = 9.6 Hz **9B**), 5.31 (s, 0.5 H, H-6, **9B**), 5.38 (d, 0.5 H, H-3′, *J*_3′,4′_ = 8.4 Hz **9A**), 5.46 (d, 0.5 H, H-3′, *J*_3′,4′_ = 8.4 Hz **9B**), 5.56 (s, 1 H, H-1′ and H-2′, **9A**), 5.82 (dd, 0.5 H, H-1′, *J*_1′,2′_ = 9.6 Hz **9B**), 6.08 (s, 0.5 H, H-6, **9A**), 6.49 (s, 0.5 H, H-3, **9B**), 6.74 (s, 0.5 H, H-3, **9A**), ^13^C-NMR (CDCl_3_, 100 MHz) δ_C_ ppm: 19.1, 20.44, 20.47, 20.5. 20.61, 20.63, 20.67, 20.72, 20.77, 20.85, 20.89, 22.7, 23.9, 24.6 (CH_3_ and 7 X CH_3_CO, **9A** and **9B**), 61.5 (C-5′ **9A**), 61.8 (C-5′ **9B)**, 67.1 (C-2′ **9A**), 67.2, 67.6 (C-3′ **9A** and **9B**), 67.9, (C-1′ **9A**), 68.11 (C-3 **9A**), 68.4 (C-4′ **9A** and **9B**), 68.7 (C-1′ **9B**), 69.9 (C-2′ **9B**), 72.1 (C-3 **9B**), 98.0 (C-6 **9B**), 108.2 (C-6 **9A**), 147.35, 147.38 (C-5 **9A** and C-7 **9B**), 151.07, 151.09 (C-8a **9A** and **9B**), 158.1 (C-7 **9A**), 158.3 (C-5 **11B**), 165.4, 169.1, 169.2, 169.5, 169.8, 169.92, 169.93, 169.99, 170.0, 170.2, 170.3, 170.63, 170.69, 175.5 (7 X CH_3_CO **9A** and C-7 **9B**). Anal. Calc. for C_25_H_32_N_4_O_13_: C, 50.33, H, 5.41, N, 9.39. Found: C, 50.39, H, 5.61, N, 9.26.

### 4.2. Biological Evaluation

#### 4.2.1. Cytotoxicity Screening

The compounds’ cytotoxicity on normal and cancer cells was assayed by MTT as reported [81,82,105,106] and detailed in the Supplementary Materials.

#### 4.2.2. Enzymatic Assays

VEGFR-2 inhibition was detected by VEGFR-2 (KDR) Kinase Assay Kit-BPS Bioscience Corporation catalog # 40325 [107]. MMP-2 inhibitory activities were evaluated utilizing MMP-2 inhibitor screening kit (Colorimetric) catalog # ab139446 [108]. CA II inhibition assay was performed as reported [109]. The protocols are detailed in the Supplementary Materials.

### 4.3. Docking

The docking protocol was detailed in the Supplementary Materials.

### 4.4. Statistics

IC_50_ values were calculated by the Graphpad Instat software Version 3 01. Statistical significance was estimated by ANOVA using the SPSS16 program.

## 5. Conclusions

The current study portrays simultaneous inhibition of MMP-2, CA II, and VEGFR-2 via newly synthesized hybrid 1,2,4-triazolo[4,3-*a*]pyrimidinone acyclo C-nucleosides. The hit derivatives **8** and **9** were nanomolar inhibitors of VEGFR-2/MMP-2 and submicromolar inhibitors of CA II. Docking simulations predicted their binding to key residues in the active sites of the studied enzymes and highlighted the regioselectivity as determinant of activity. Both compounds were safer than doxorubicin on normal human cells. Their anticancer activities against three human cancers echoed their enzymatic activities. To the best of our knowledge, the reported triazolopyrimidinone acyclo C-nucleosides are the first in class VEGFR-2/MMP-2/CA II inhibitors that deserve further studies.

## Figures and Tables

**Table 1:** Comparative study toward the synthesis of sugar hydrazones **2**–**5**.

Compound No.	Conventional Method		Ultrasound Irradiation	
	Time (h)	Yield (%)	Time (min)	Yield (%)
**2**	7	61	45	92
**3**	8	71	90	89
**4**	10	65	120	88
**5**	8	76	90	93

**Table 2:** Cytotoxicity and anticancer selectivity profiles of the pyrimidine sugar hydrazones **2**–**5** and 1,2,4-triazolo[4,3-*a*]pyrimidinone acyclo C-nucleosides **6**–**9**.

Compound No.	Wi-38		MDA-MB 231		Caco-2		HepG-2	
	EC_100_ (nM)	IC50 (nM)	IC_50_ (nM)	SI ^a^	IC_50_ (nM)	SI	IC_50_ (nM)	SI
**2**	46.35 ± 1.35	92.86 ± 0.35	100.2 ± 2.99	0.93	127.8 ± 1.48	0.73	3548 ± 35.5	0.02
**3**	84.54 ± 1.00	176.3 ± 4.14	75.77 ± 1.25	2.33	96.49 ± 0.09	1.83	3487 ± 182	0.05
**4**	64.61 ± 0.68	128.3 ± 2.96	93.69 ± 2.69	1.37	108.1 ± 0.62	1.19	4940 ± 101	0.03
**5**	48.16 ± 1.66	102.5 ± 1.25	83.29 ± 3.06	1.23	111.1 ± 2.94	0.92	3816 ± 126	0.03
**6**	353.7 ± 9.54	587.1 ± 4.48	46.28 ± 3.32	12.69	54.06 ± 0.47	10.86	4628 ± 33.0	0.13
**7**	664.3 ± 26.60	872.5 ± 16.3	39.99 ± 0.04	21.82	47.44 ± 0.38	18.39	3729 ± 252	0.23
**8**	**129.11 ± 10.40**	316.0 ± 4.00	**1.077 ± 0.02**	293.41	**2.472 ± 0.10**	127.83	**4.565 ± 0.32**	69.22
**9**	**358.88 ± 20.70**	722.5 ± 12.5	**3.636 ± 0.03**	198.71	**4.244 ± 0.53**	170.24	**9.53 ± 0.07**	75.81
**Doxorubicin**	**11.50 ± 0.50**	30.00 ± 1.00	**10.6 ± 0.10**	2.83	**48.3 ± 30**	0.62	**22.5 ± 10**	1.33

All values are expressed as mean ± SEM. ^a^ SI: selectivity index equals the ratio between the compound’s IC_50_ on normal cells and its IC_50_ on cancer cells.

**Table 3:** In vitro VEGFR-2 inhibitory activities of **8** and **9** and their ligand efficiency metrics.

Compound No.	IC_50_ (nM)	pIC_50_^a^	LE ^b^	LLE ^c^	LELP ^d^
**8**	5.89 ± 0.6	8.229	0.26	8.819	−2.27
**9**	10.52 ± 1.6	7.977	0.25	8.567	−2.36
**Sorafenib**	4.92 ± 0.5	-	-	-	-

^a^ pIC_50_: -logIC_50_, ^b^ LE: ligand efficiency [89], ^c^ LLE: lipophilic ligand efficiency [90], ^d^ LELP: ligand efficiency-dependent lipophilicity index [88].

**Table 4:** In vitro MMP-2 inhibitory activities of **8** and **9** and their ligand efficiency metrics.

Compound No.	IC_50_ (nM)	pIC_50_	LE	LLE	LELP
**8**	17.44 ± 1.4	7.758	0.25	8.348	−2.36
**9**	30.93 ± 2.5	7.509	0.24	8.099	−2.45
**NNGH**	298 ± 20.2	-	-	-	-

**Table 5:** In vitro CAII inhibitory activities of **8** and **9** and their ligand efficiency metrics.

Compound No.	IC_50_ (nM)	pIC_50_	LE	LLE	LELP
**8**	205.7 ± 21.6	6.686	0.22	7.27	−2.68
**9**	360.9 ± 32.7	6.442	0.21	7.03	−2.81
**Quercetin**	27.3 ± 2.1	-	-	-	-

## Data Availability

Not applicable.

## Supplementary Materials

The following supporting information can be downloaded at: https://www.mdpi.com/article/10.3390/molecules27082422/s1, Figures S1–S39: NMR spectrum of compounds **2**–**9**; Biological evaluation assays including cytotoxicity screening on normal human lung fibroblasts (Wi-38); Determination of the anticancer activity (Figure S40); VEGFR-2 kinase inhibitory activity assay (Figure S41); MMP-2 inhibitory activity assay (Figure S42); CA II inhibition assay (Figure S43). References [81,82,82,108,109] are cited in the supplementary materials.

## Sample Availability

Samples of the compounds **2-9** are available from the authors.
