# Supplementary material for: Enhancing the Anticancer Potential of Targeting Tumor-Associated Metalloenzymes via VEGFR Inhibition by New Triazolo[4,3-a]pyrimidinone Acyclo C-Nucleosides Multitarget Agents
Source: Molecules. 2022 Apr 8;27(8):2422. doi: 10.3390/molecules27082422 (PMC9026109; doi:10.3390/molecules27082422)
Supplement: Supplementary file 1 [file molecules-27-02422-s001.zip › molecules-1612410-supplementary.pdf]

Supplementary Information

Enhancing the anticancer potential of targeting tumor-associated metalloenzymes *via* VEGFR inhibition by new triazolo[4,3-*a*]pyrimidinone acyclo C-nucleosides multitarget agents

Mohamed Nabil Abd Al Moaty [1], El Sayed Helmy El Ashry [1], Laila Fathy Awad [1,*], Nihal Ahmed Ibrahim [1], Marwa Muhammad Abu-Serie [2], Assem Barakat [3,*], Mezna Saleh Altowyan [4] and Mohamed Teleb [5]

[1] Chemistry Department, Faculty of Science, Alexandria University, Alexandria 21321, Egypt; mohamednabil_sc_chem@yahoo.com (M.N.A.A.M.); eelashry60@hotmail.com (E.S.H.E.A.); nihalawadallah@outlook.com (N.A.I.)
[2] Medical Biotechnology Department, Genetic Engineering and Biotechnology Research Institute, City of Scientific Research and Technological Applications (SRTA-City), Alexandria 21934, Egypt; marwaelhedaia@gmail.com
[3] Department of Chemistry, College of Science, King Saud University, P.O. Box 2455, Riyadh 11451, Saudi Arabia
[4] Department of Chemistry, College of Science, Princess Nourah bint Abdulrahman University, P.O. Box 84428, Riyadh 11671, Saudi Arabia; msaltowyan@pnu.edu.sa
[5] Department of Pharmaceutical Chemistry, Faculty of Pharmacy, Alexandria University, Alexandria 21521, Egypt; mohamed.t.ismail@alexu.edu.eg
* Correspondence: laila.fathy@yahoo.com (L.F.A.); ambarakat@ksu.edu.sa (A.B.)

Content **Page**

1. NMR spectra of compounds 2-9 S2-S21

2. Biological evaluation S21
2.1. Cytotoxicity screening on normal human lung fibroblasts (Wi-38) S21
2.2. Determination of the anticancer activity S21
2.3. Procedure and data analysis of VEGFR-2 inhibition assay S23
2.4. Procedure and data analysis of MMP-2 inhibition assay S24
2.5. Procedure and data analysis of CAII inhibition assay S26

3. Docking simulations S27
References S28

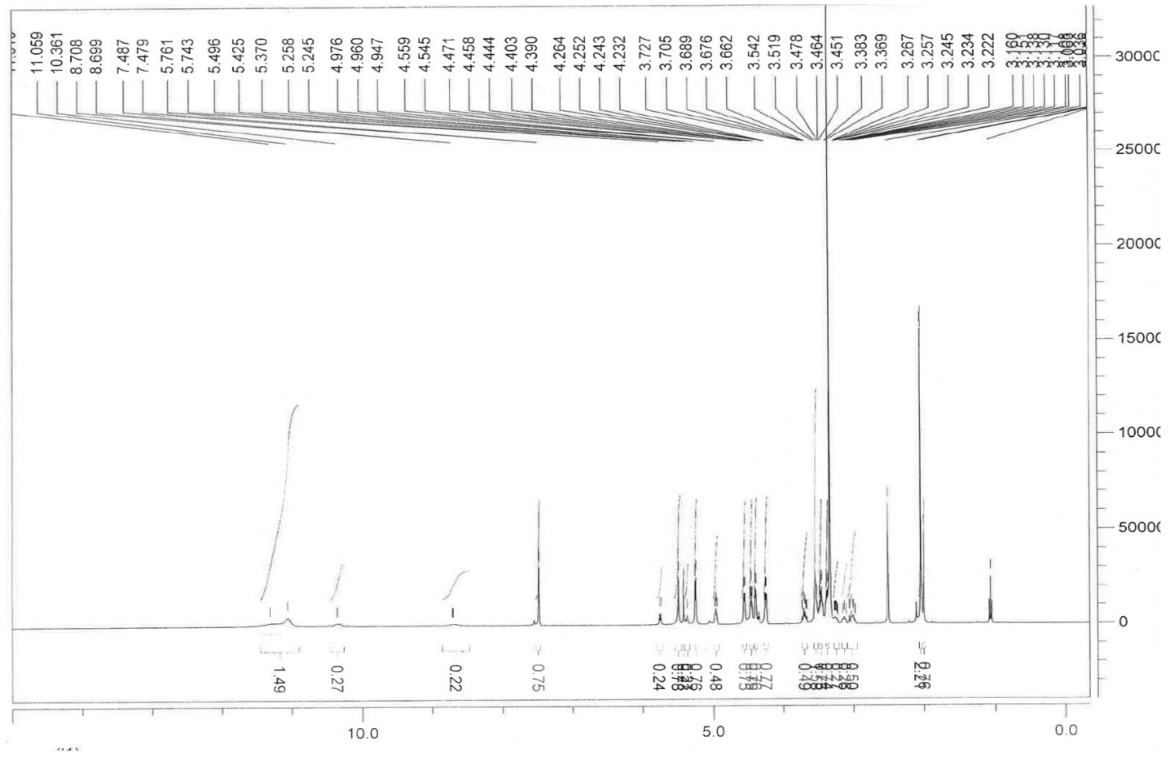

Figure S1. ^{1}H-NMR Spectrum of compound **2** (DMSO-d_6, 400 MHz).

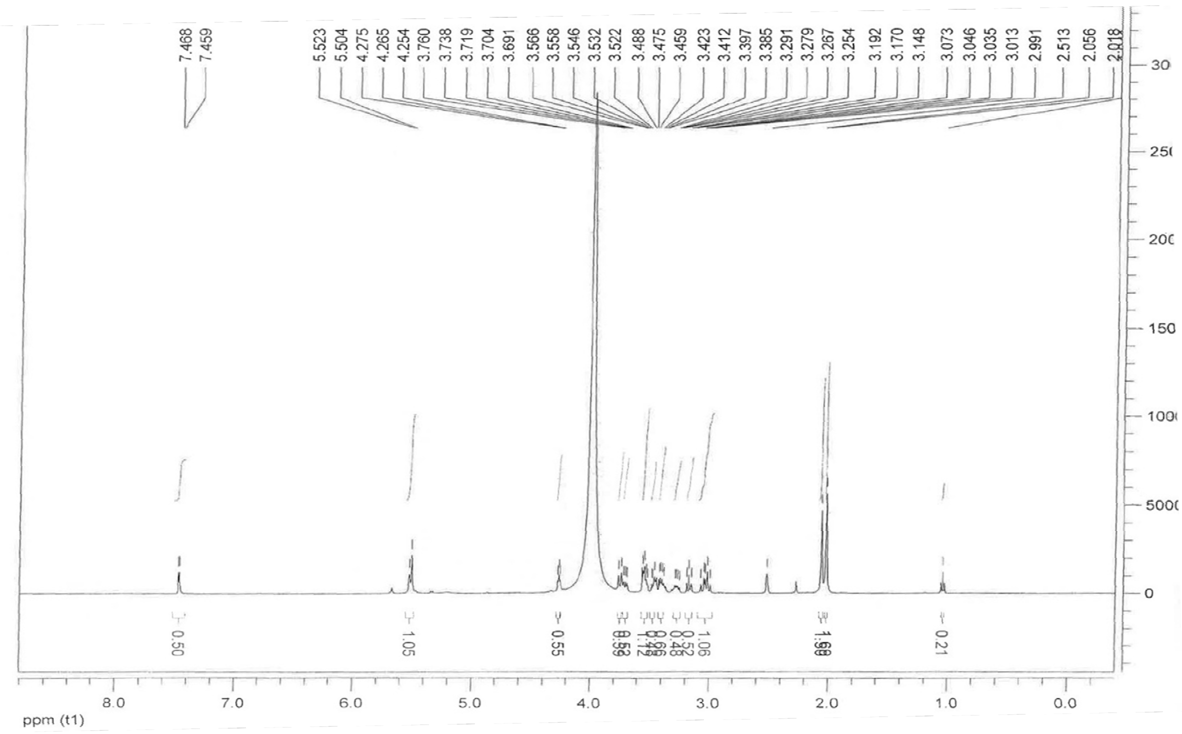

Figure S2. ^{1}H-NMR Spectrum of compound **2** (DMSO-d_6 + D_2O, 400 MHz).

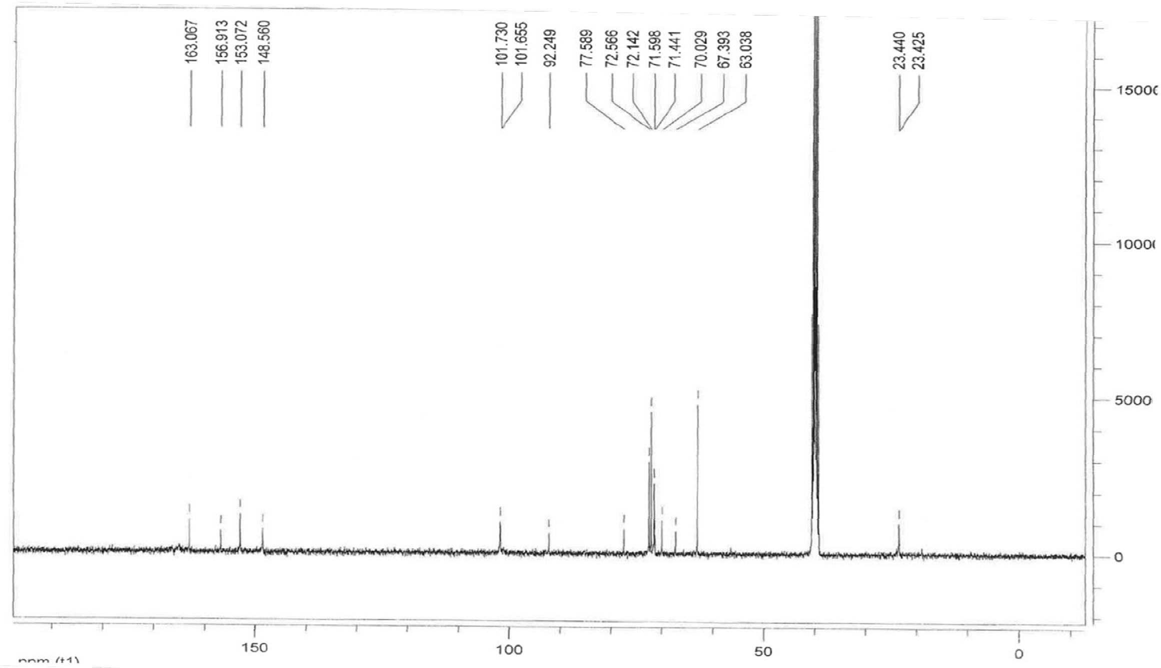

Figure S3. ^{13}C-NMR Spectrum of compound **2** (DMSO-d_6, 100 MHz).

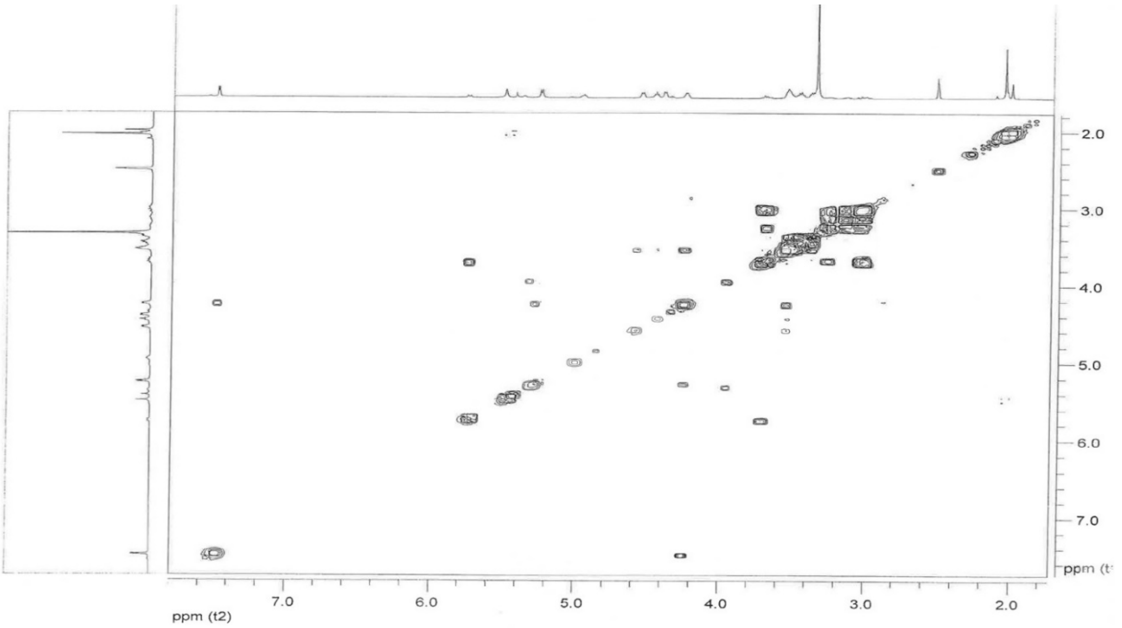

Figure S4. ^{1}H-^{1}H NMR (DQF COSY) Spectrum of compound **2** (DMSO-d_6, 400 MHz).

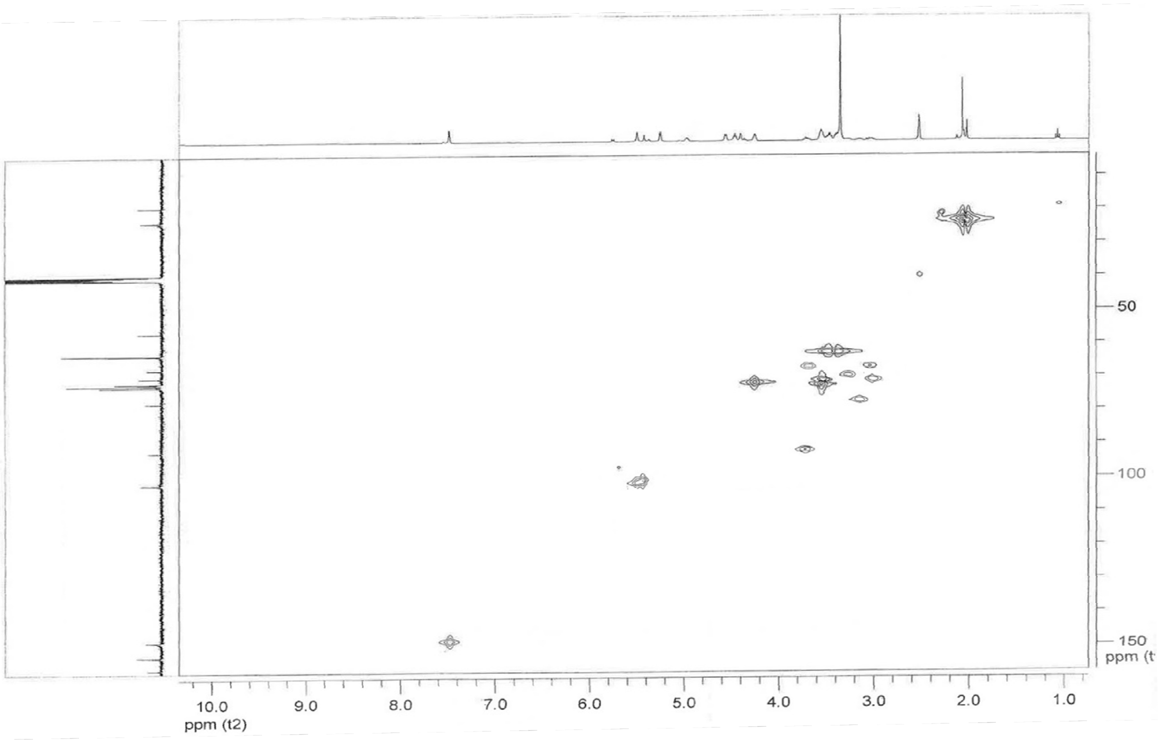

Figure S5. ^{1}H-^{13}C NMR (HMQC) Spectrum of compound **2** (DMSO-d_6, 400 MHz).

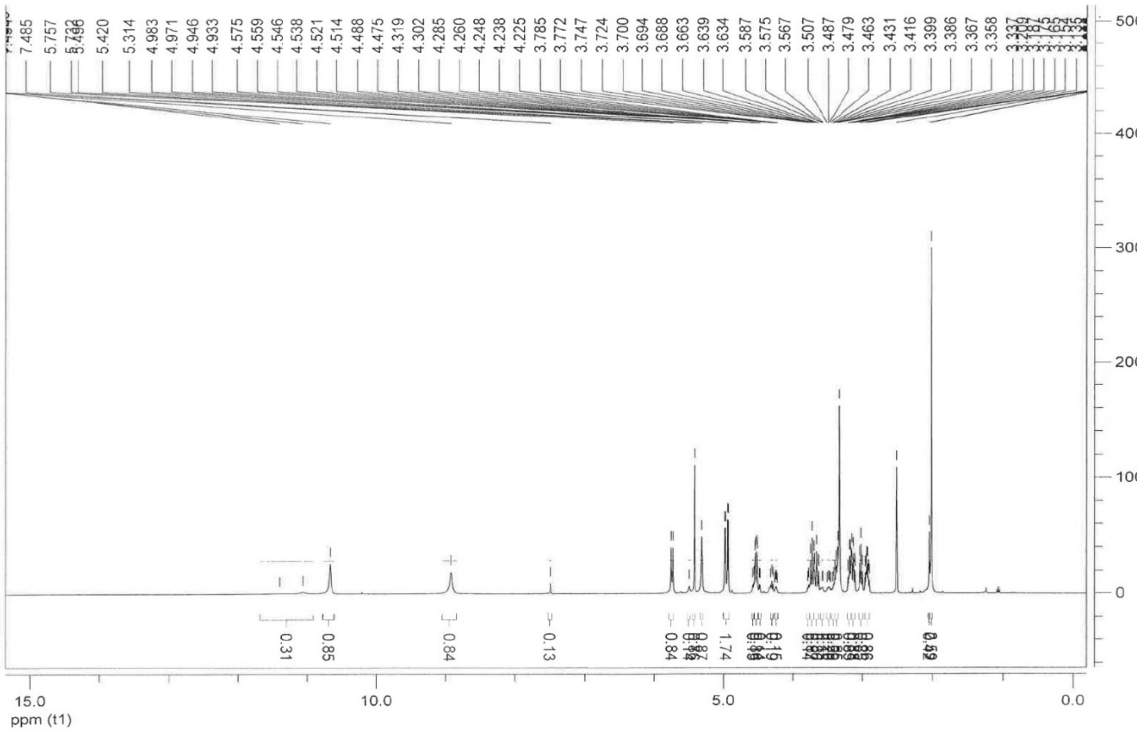

Figure S6. ^{1}H-NMR Spectrum of compound **3** (DMSO-d_6, 400 MHz).

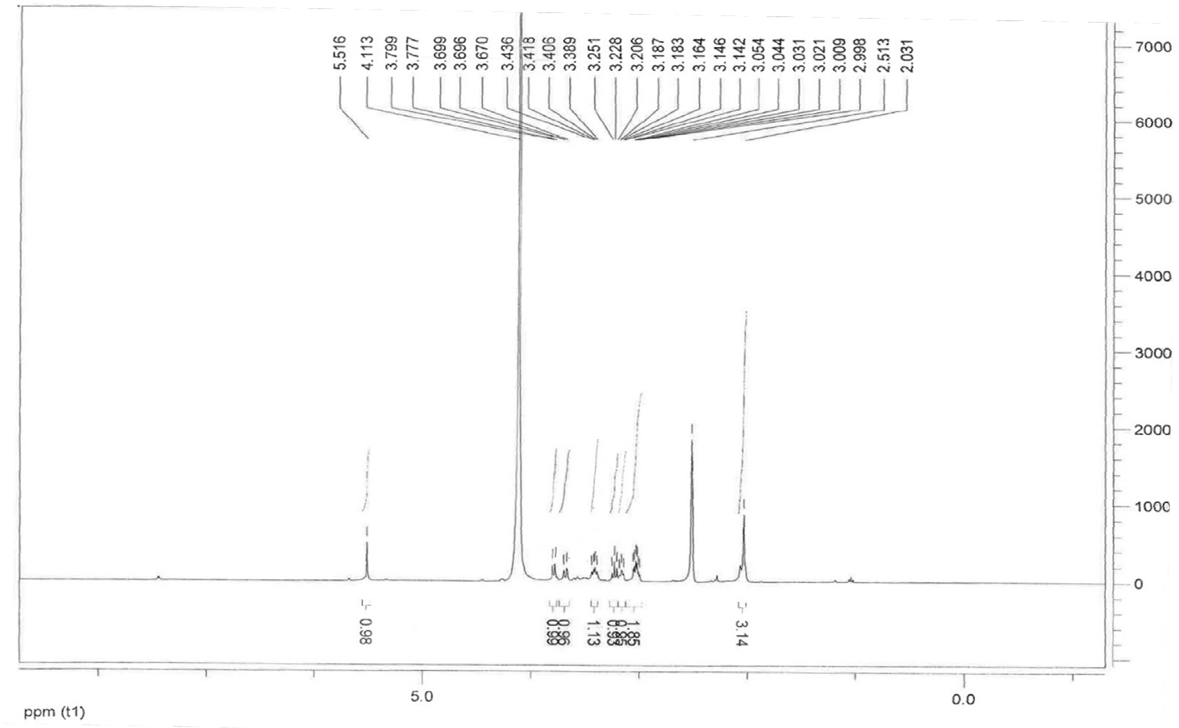

Figure S7. ^{1}H-NMR Spectrum of compound **3** (DMSO-d_6 + D_2O, 400 MHz).

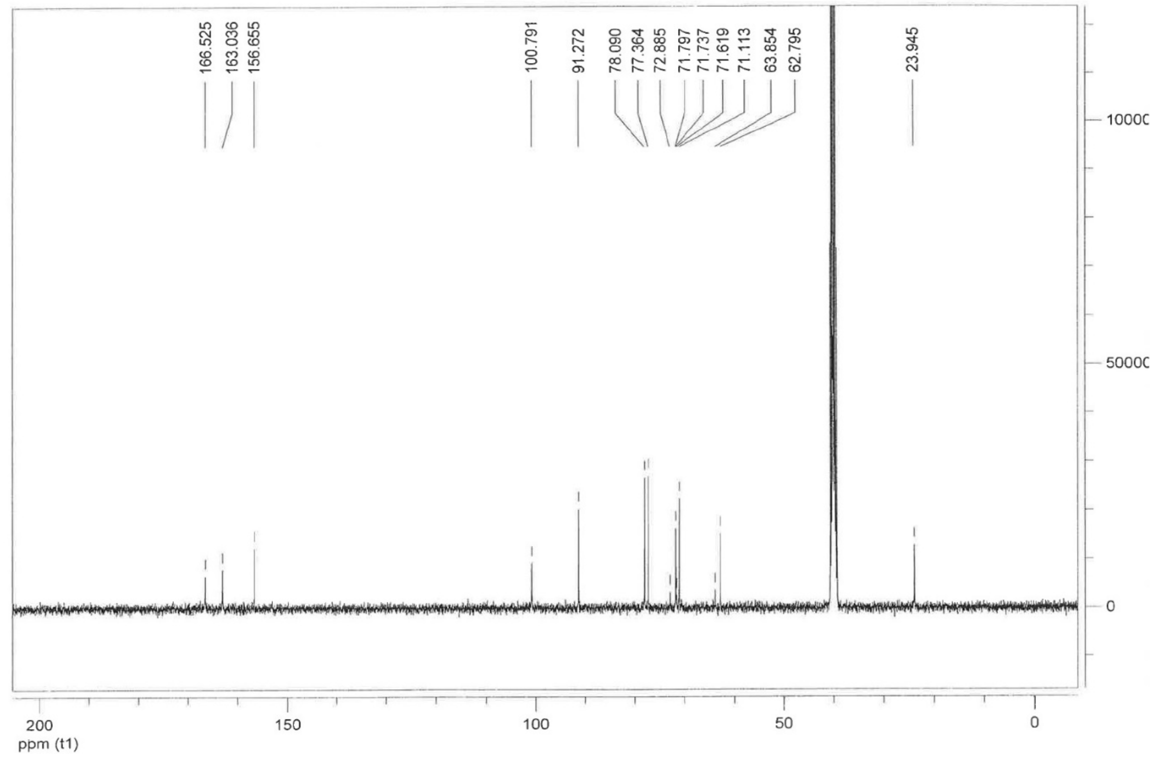

Figure S8. ^{13}C-NMR Spectrum of compound **3** (DMSO-d_6, 100 MHz).

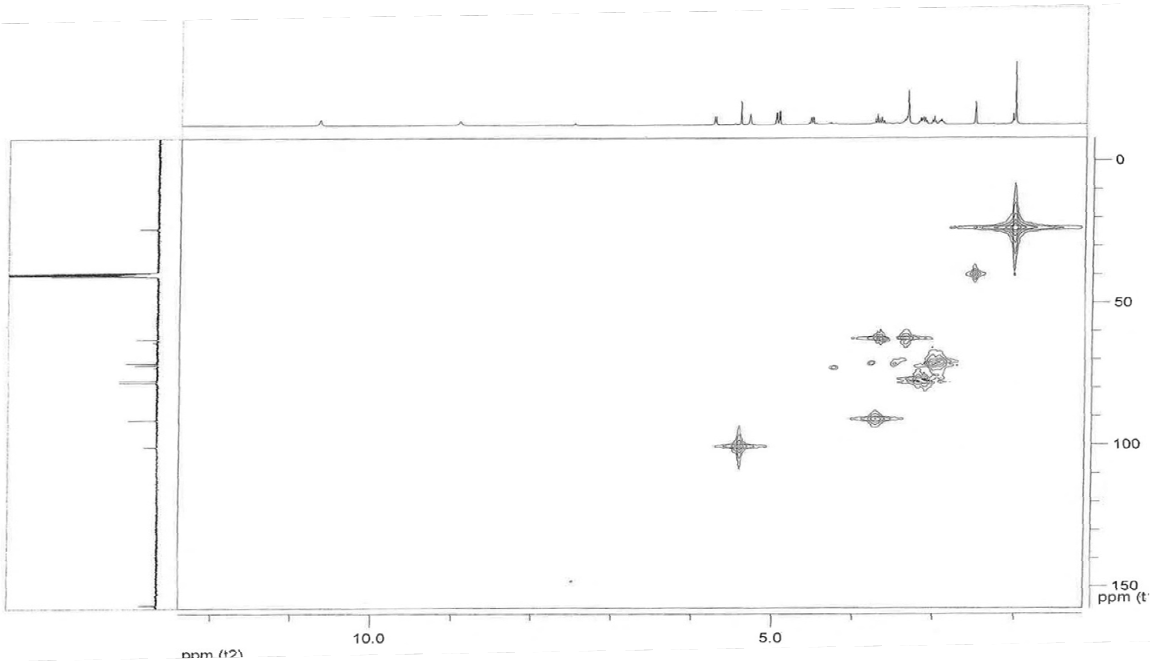

Figure S9. ^{1}H-^{13}C NMR (HMQC) Spectrum of compound **3** (DMSO-d_6, 400 MHz).

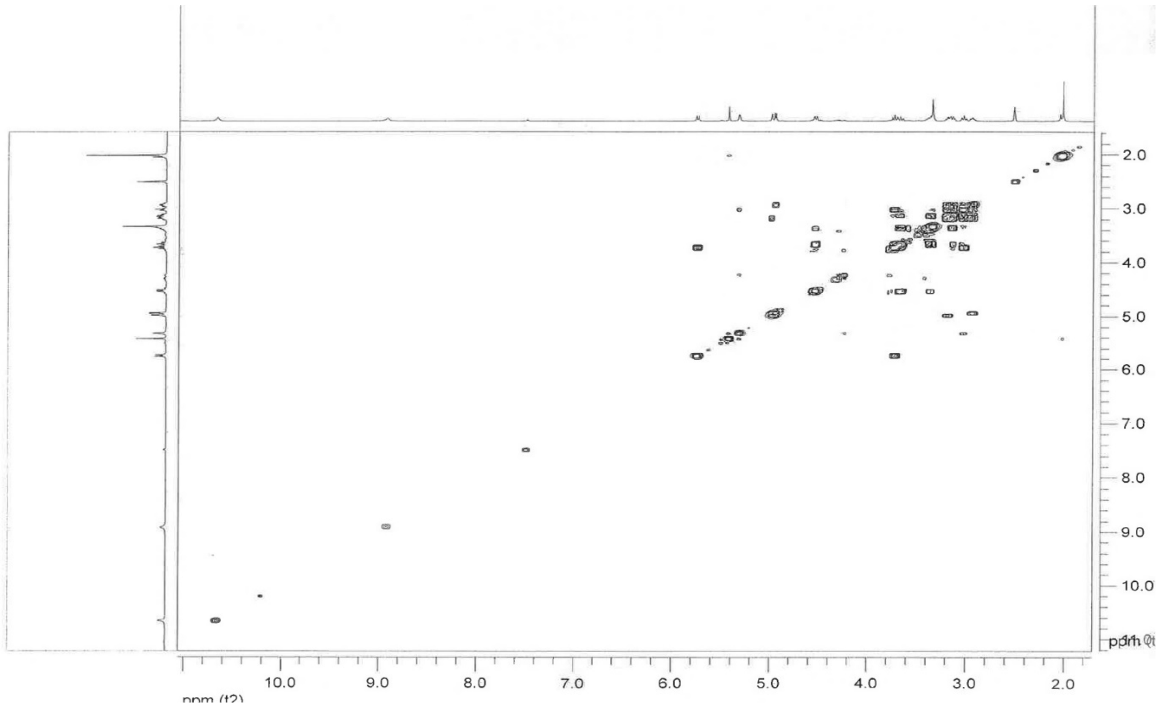

Figure S10. ^{1}H-^{1}H NMR (DQF COSY) Spectrum of compound **3** (DMSO-d_6, 400 MHz).

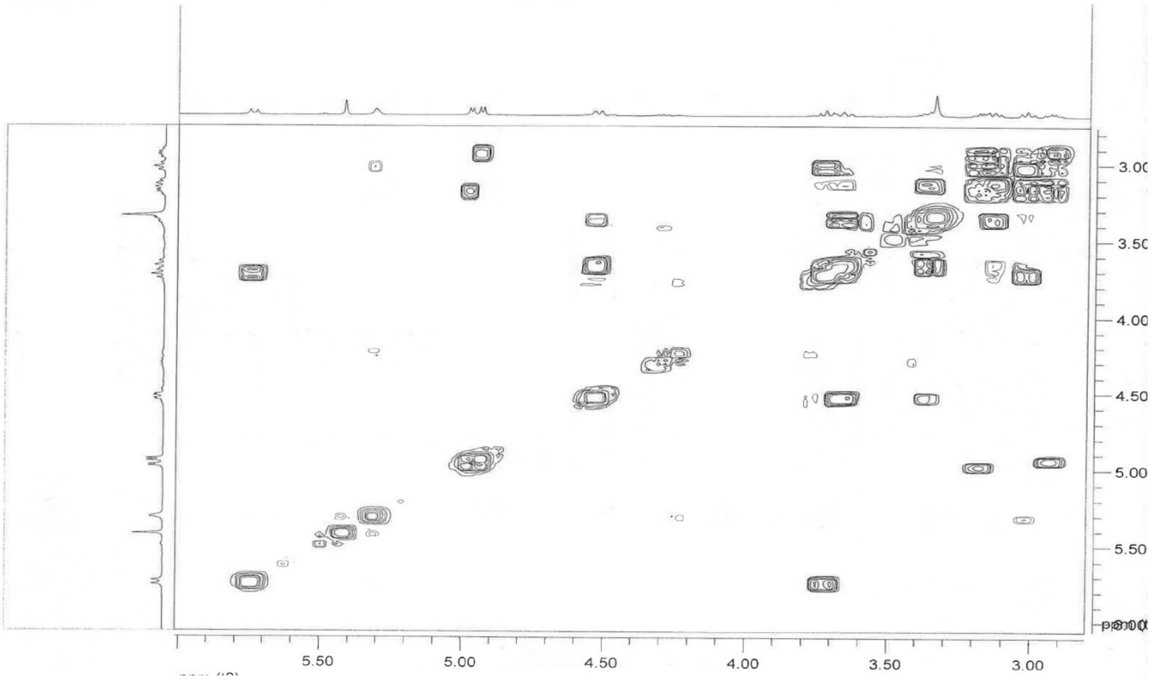

Figure S11. ^{1}H-^{1}H NMR (DQF COSY) Spectrum of compound **3**, an expansion to the sugar moiety (DMSO-d_6, 400 MHz).

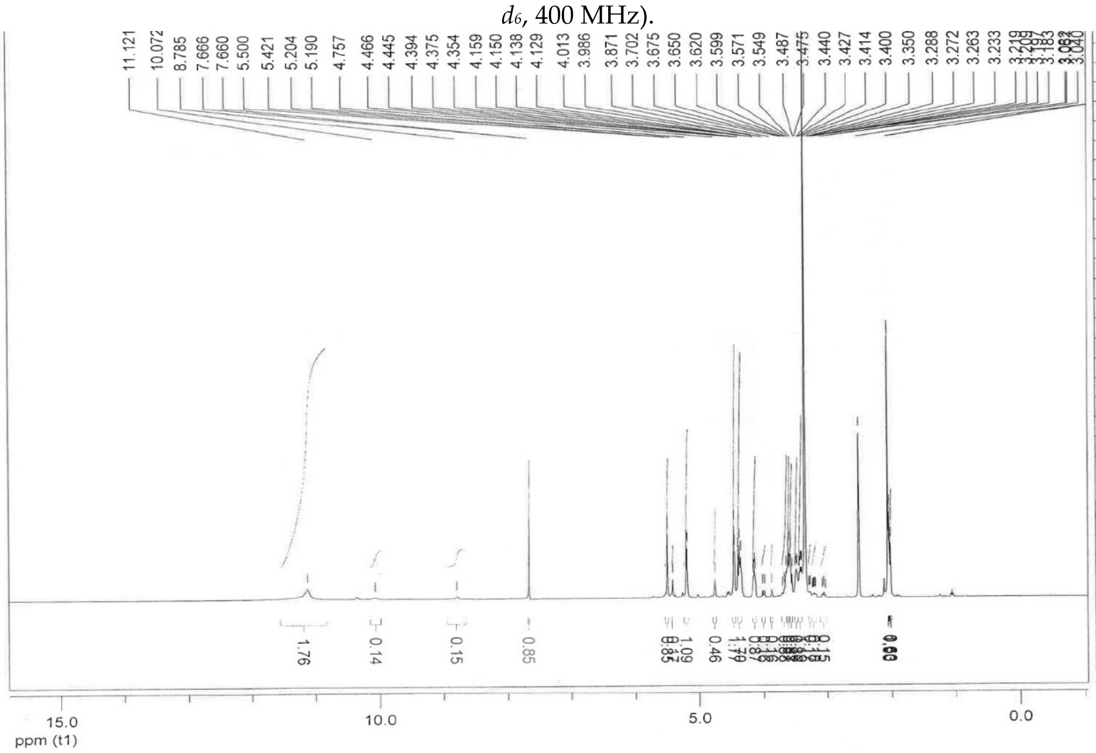

Figure S12. ^{1}H-NMR Spectrum of compound **4** (DMSO-d_6, 400 MHz).

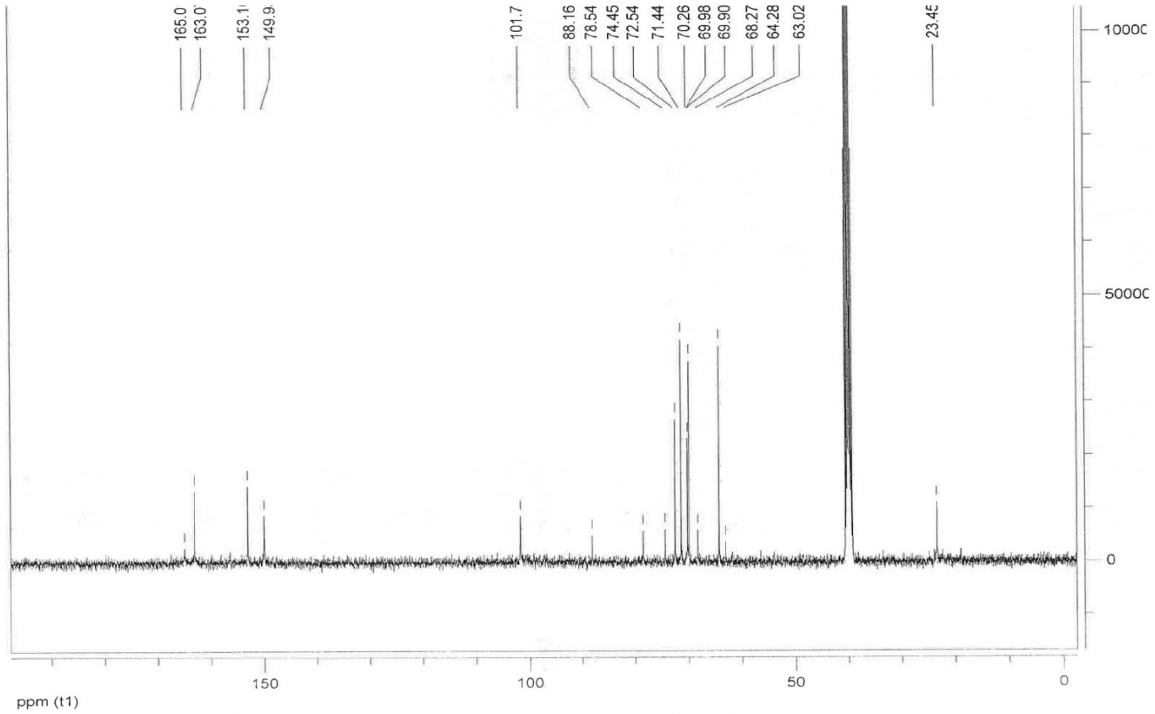

Figure S13. ^{13}C-NMR Spectrum of compound **4** (DMSO-d_6, 100 MHz).

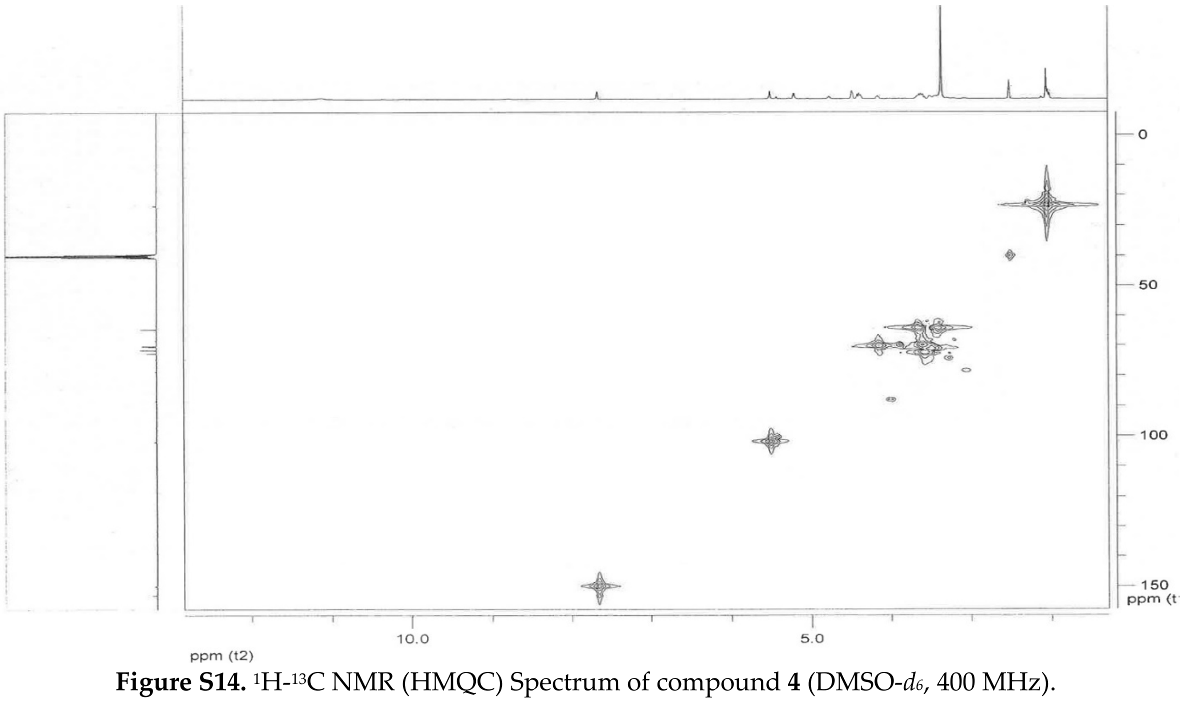

Figure S14. ^{1}H-^{13}C NMR (HMQC) Spectrum of compound **4** (DMSO-d_6, 400 MHz).

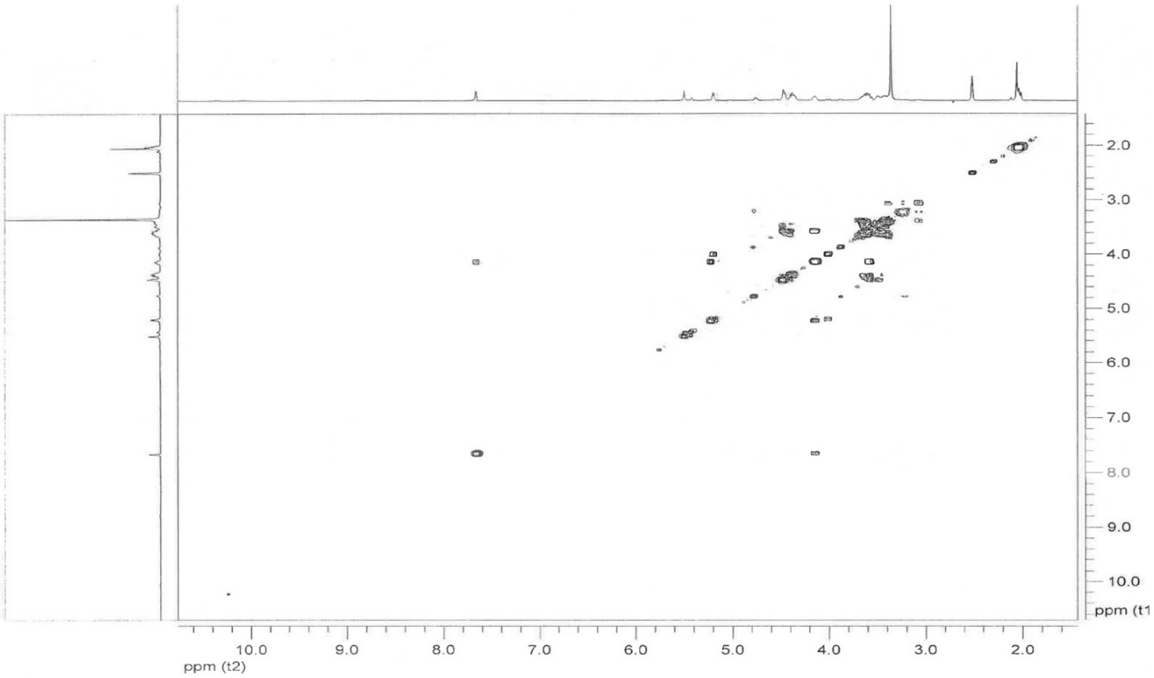

Figure S15. ^{1}H-^{1}H NMR (DQF COSY) Spectrum of compound **4** (DMSO-d_6, 400 MHz).

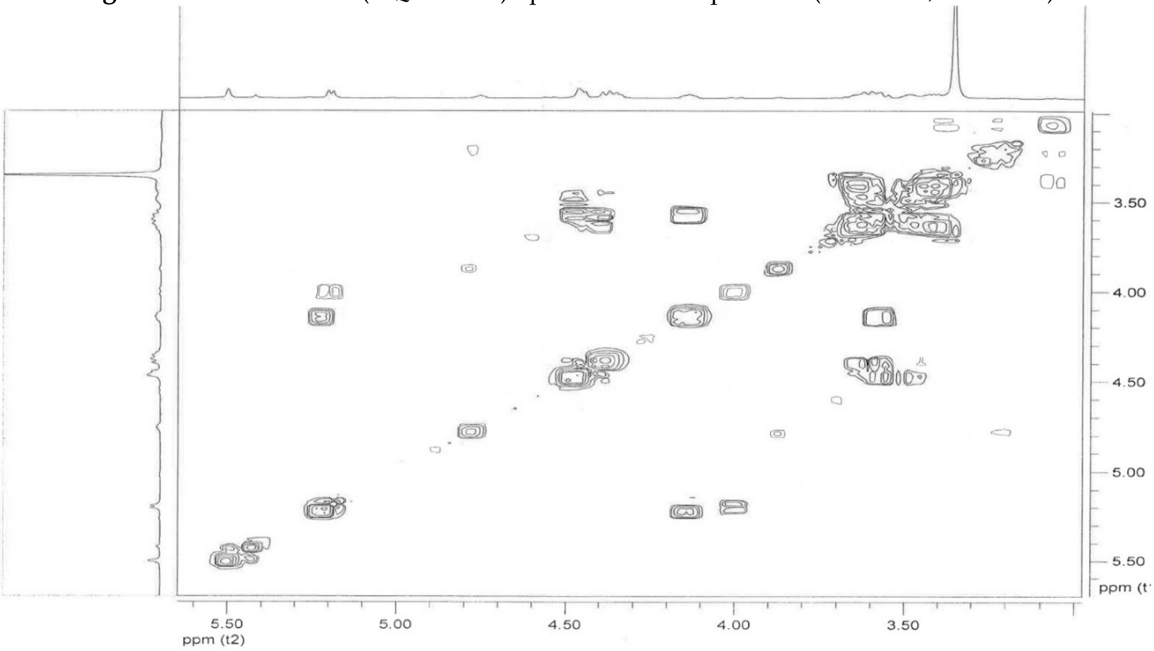

Figure S16. ^{1}H-^{1}H NMR (DQF COSY) Spectrum of compound **4**, an expansion to the sugar moiety (DMSO-d_6, 400 MHz).

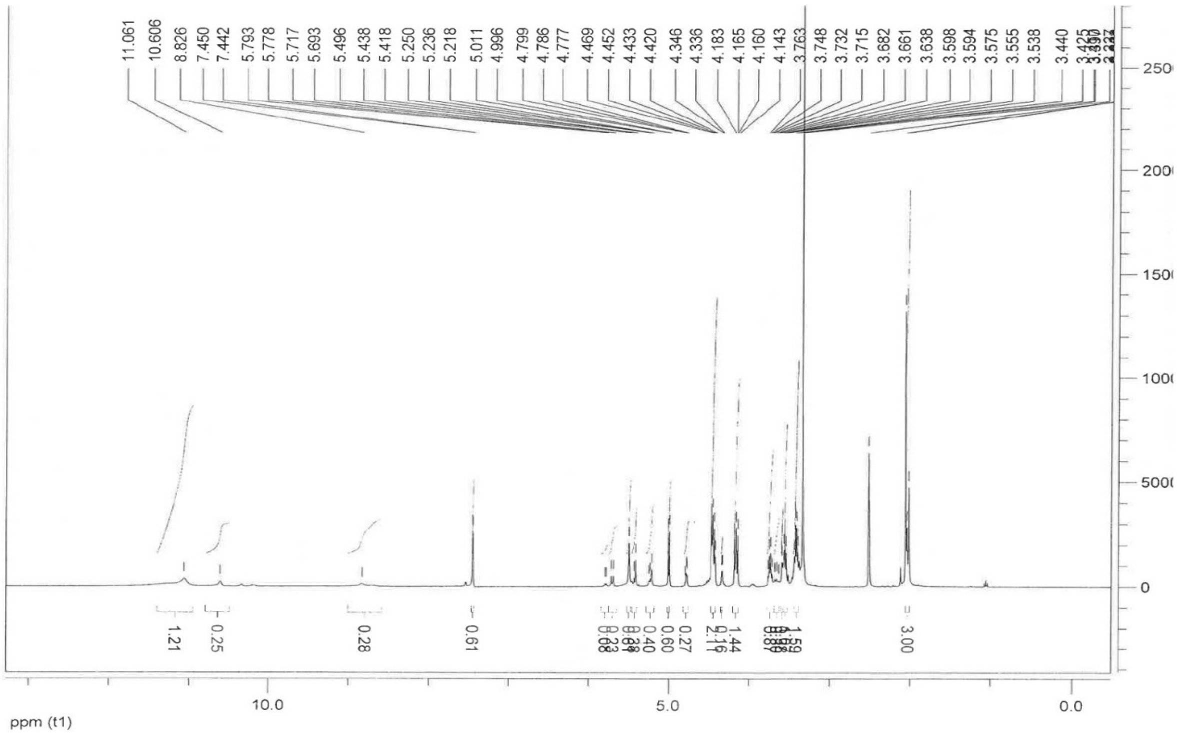

Figure S17. ^{1}H-NMR Spectrum of compound **5** (DMSO-*d*$_6$, 400 MHz).

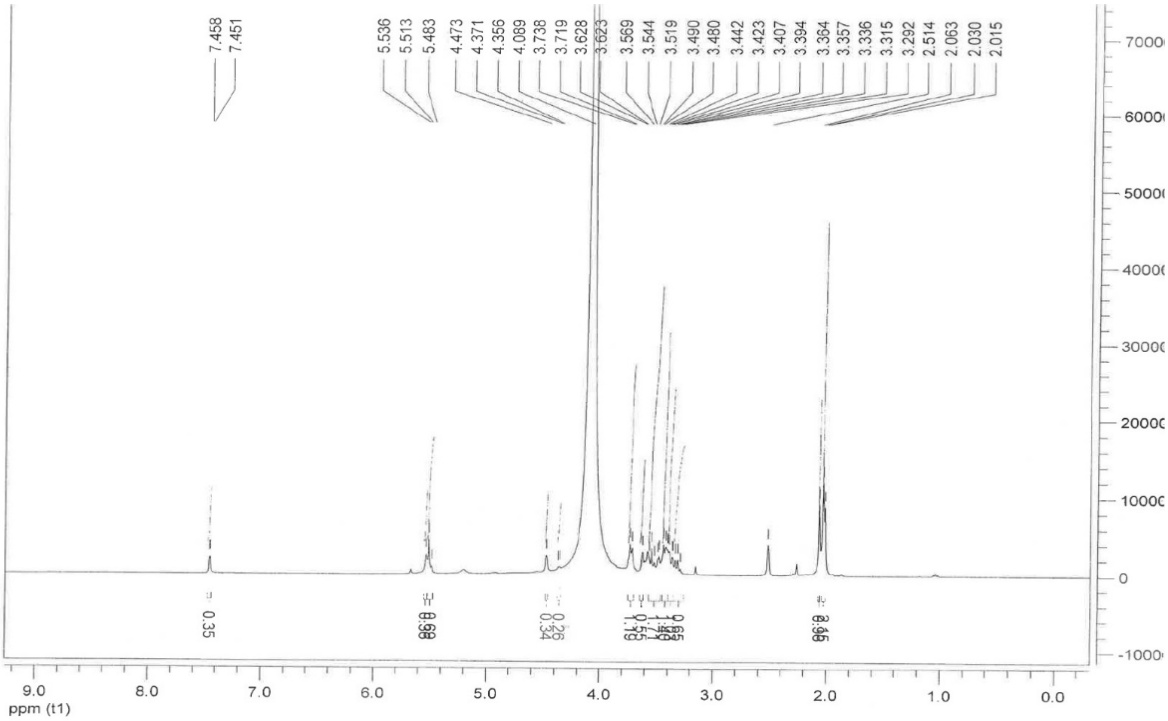

Figure S18. ^{1}H-NMR Spectrum of compound **5** (DMSO-*d*$_6$ + D_2O, 400 MHz).

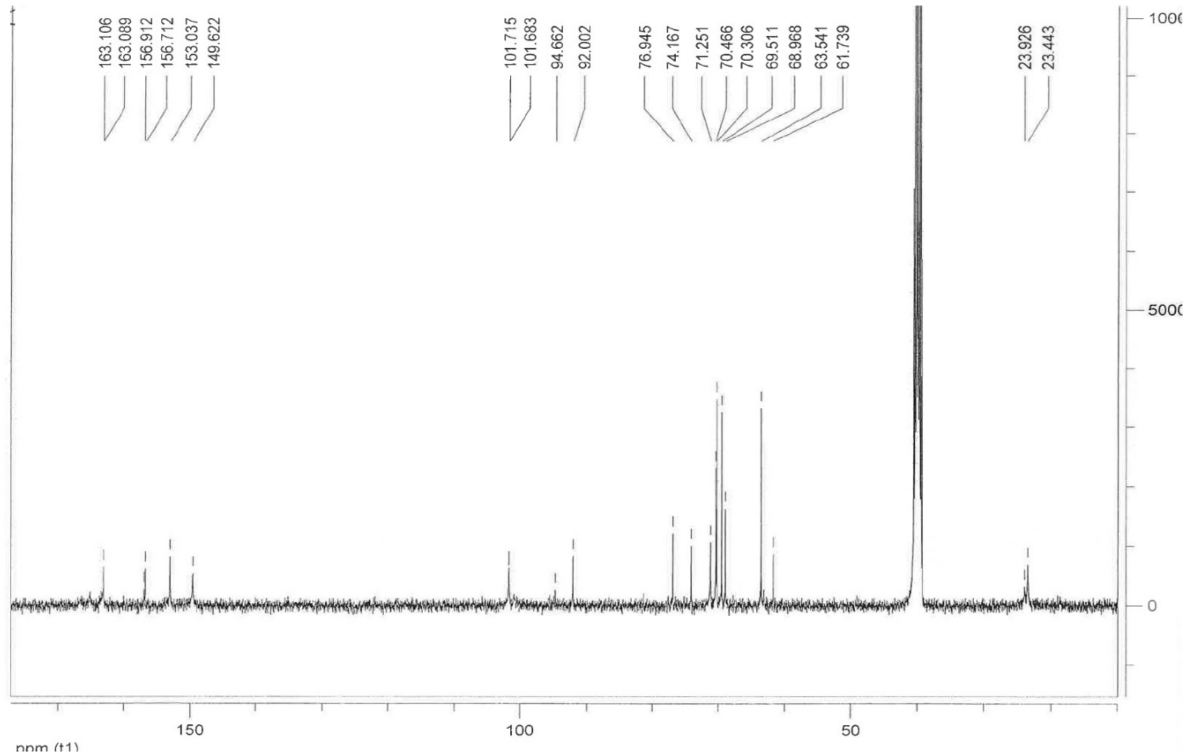

Figure S19. ^{13}C-NMR Spectrum of compound **5** (DMSO-d_6, 100 MHz).

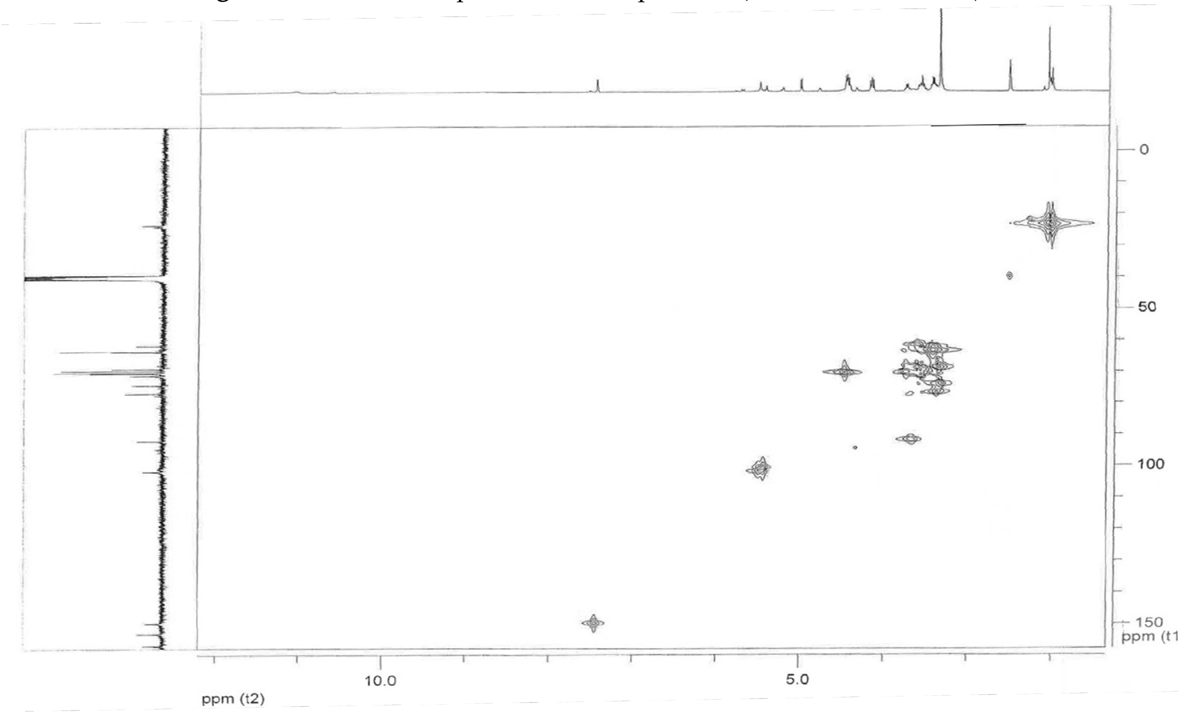

Figure S20. ^{1}H-^{13}C NMR (HMQC) Spectrum of compound **5** (DMSO-d_6, 400 MHz).

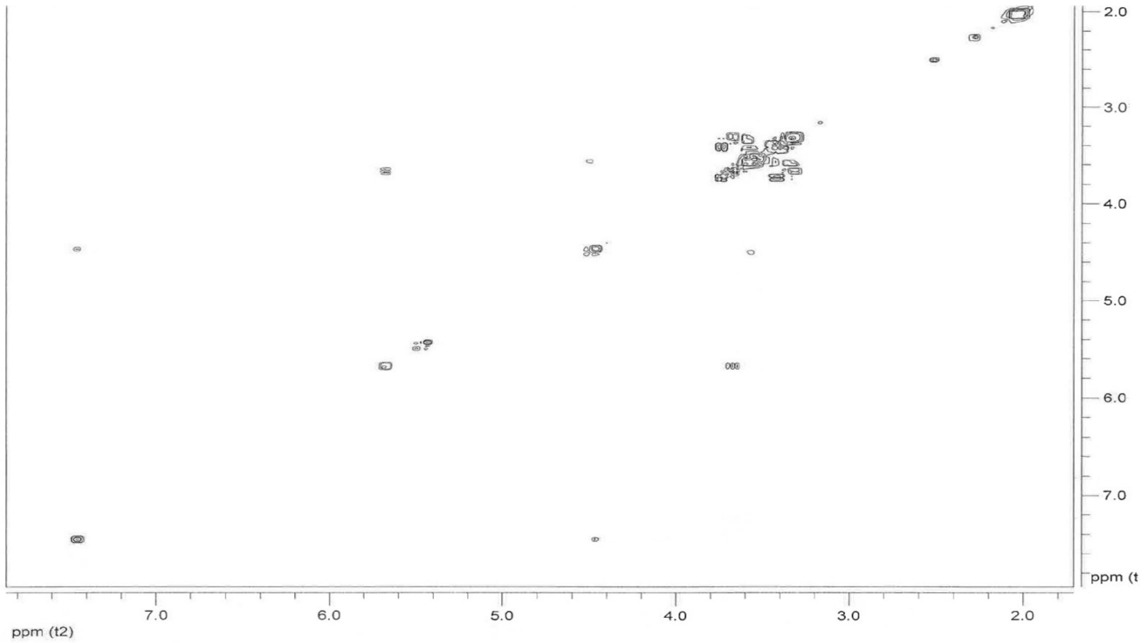

Figure S21. ^1H-^1H NMR (DQF COSY) Spectrum of compound **5** (DMSO-d_6, 400 MHz).

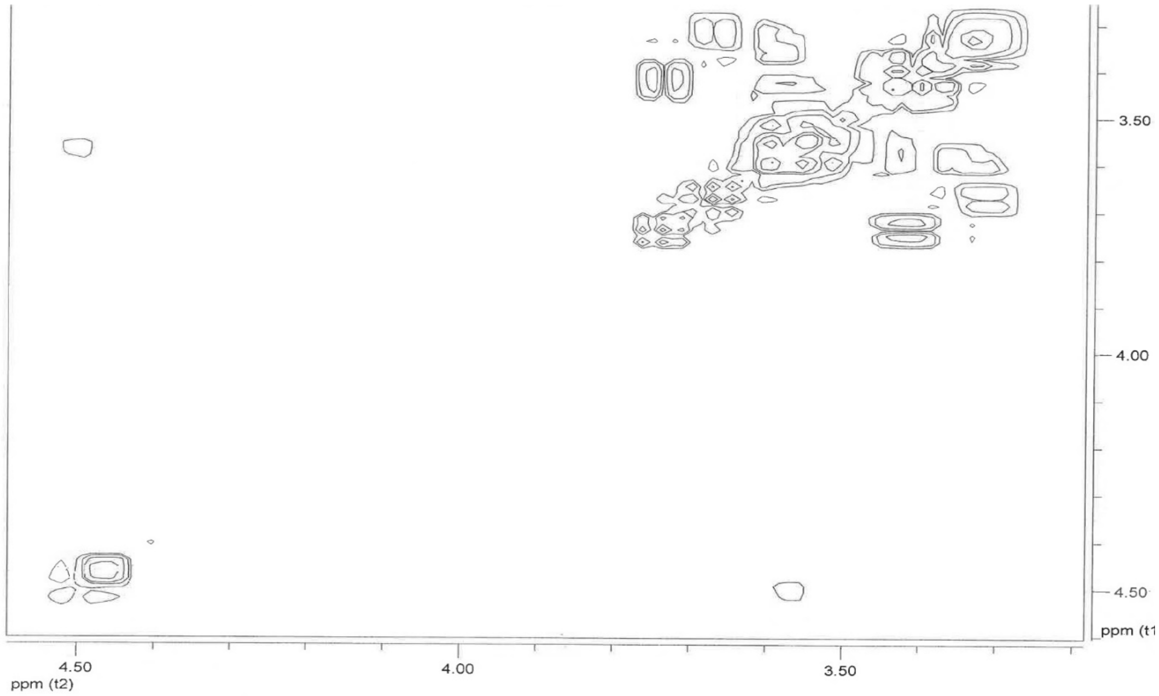

Figure S22. ^1H-^1H NMR (DQF COSY) Spectrum of compound **5**, an expansion to the sugar moiety (DMSO-d_6, 400 MHz).

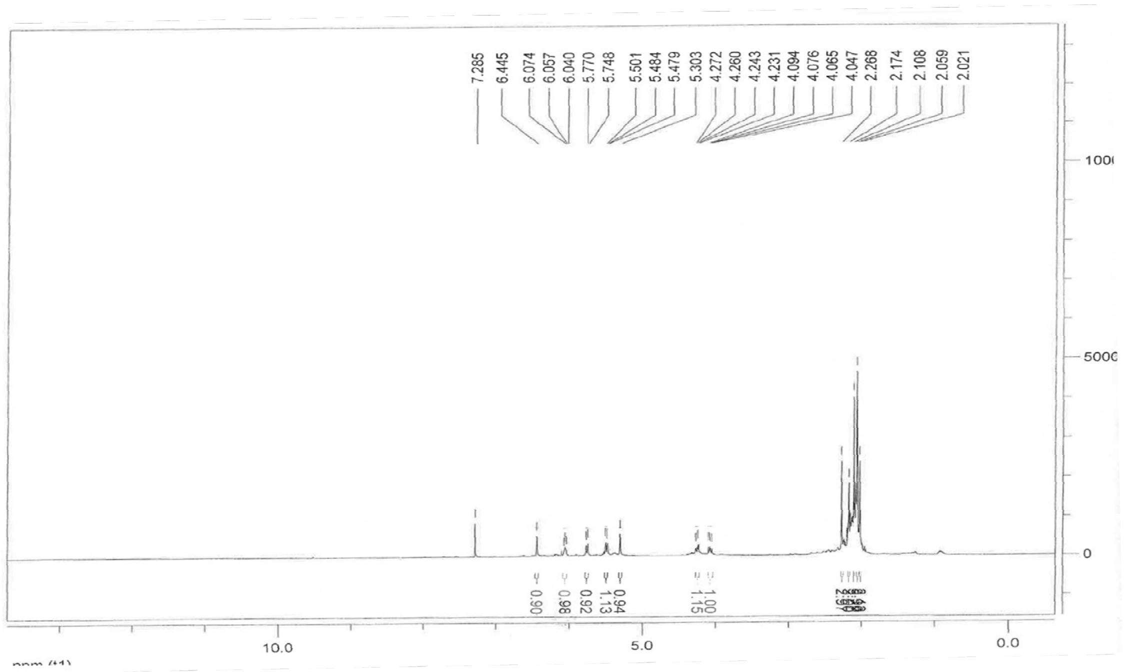

Figure S23. ^{1}H-NMR Spectrum of compound **6** ($CDCl_3$, 400 MHz).

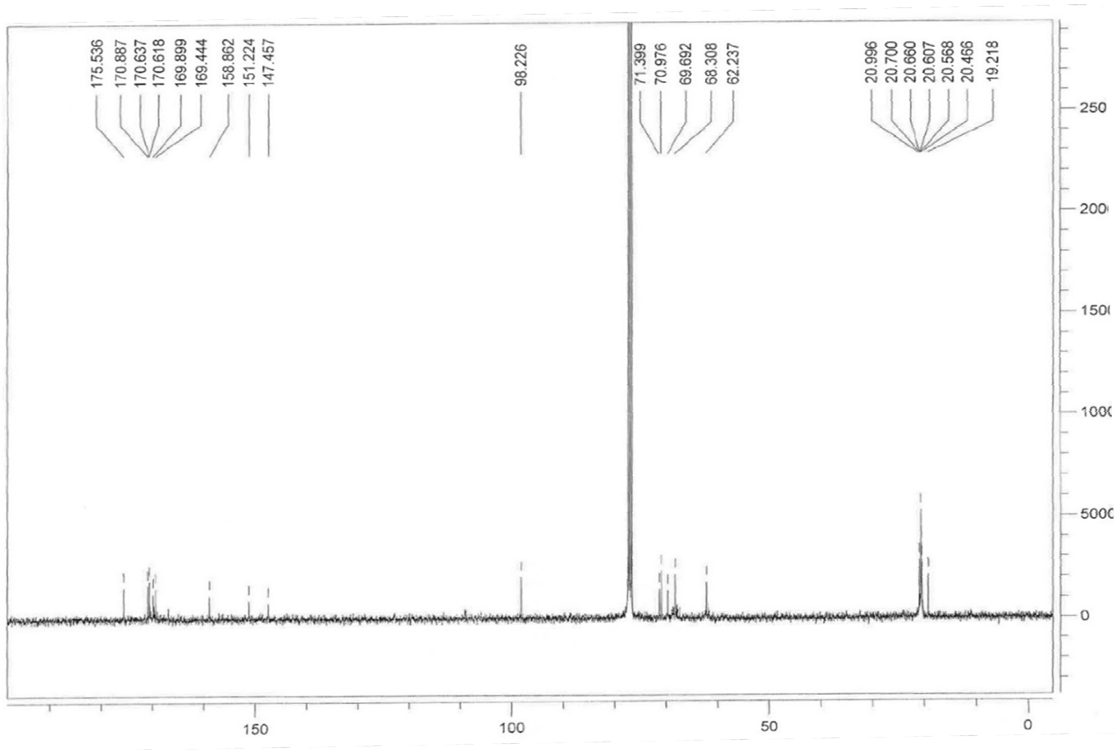

Figure S24. ^{13}C-NMR Spectrum of compound **6** ($CDCl_3$, 100 MHz).

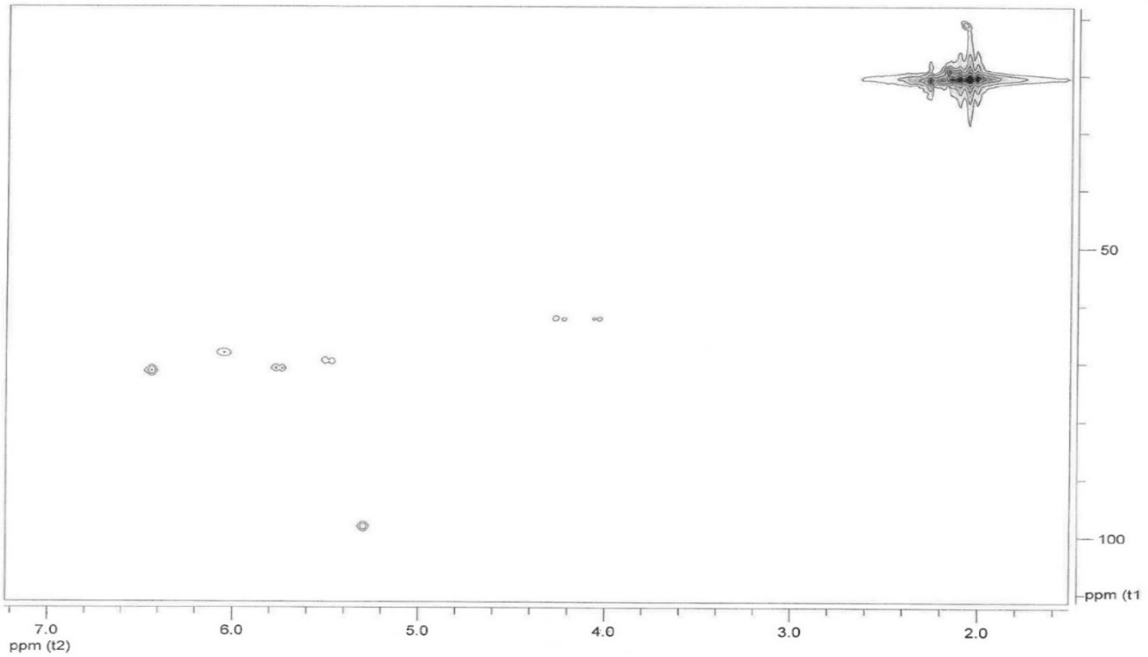

Figure S25. ^1H-^{13}C NMR (HMQC) Spectrum of compound **6** ($CDCl_3$, 400 MHz).

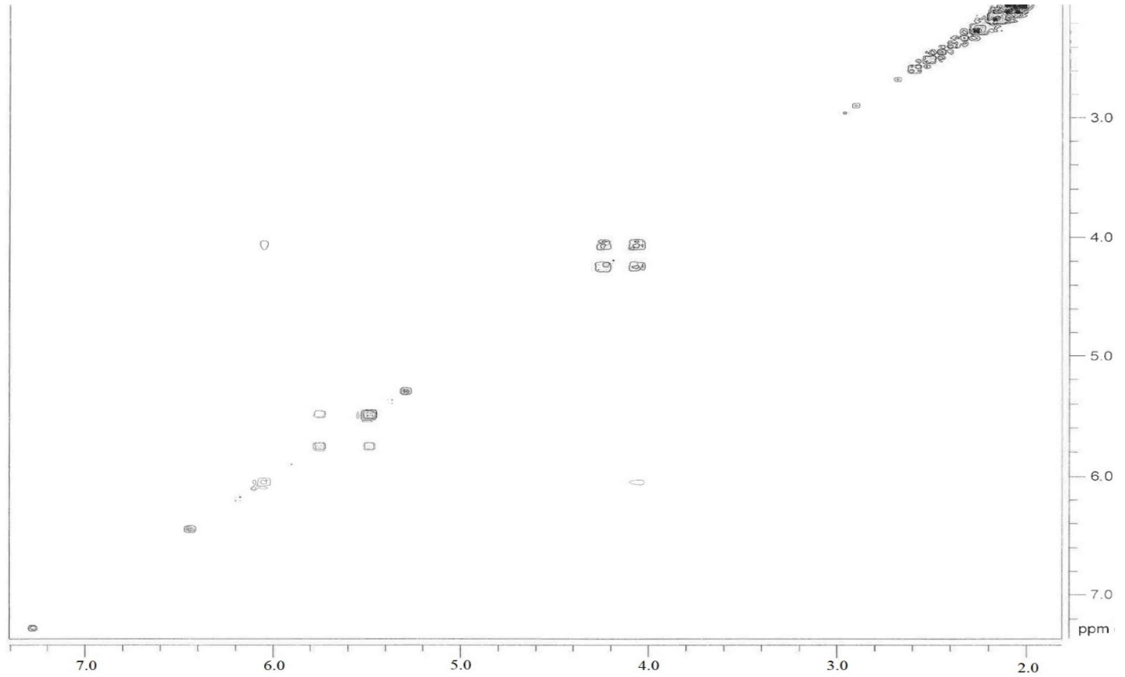

Figure S26. ^1H-^1H NMR (DQF COSY) Spectrum of compound **6** ($CDCl_3$, 400 MHz).

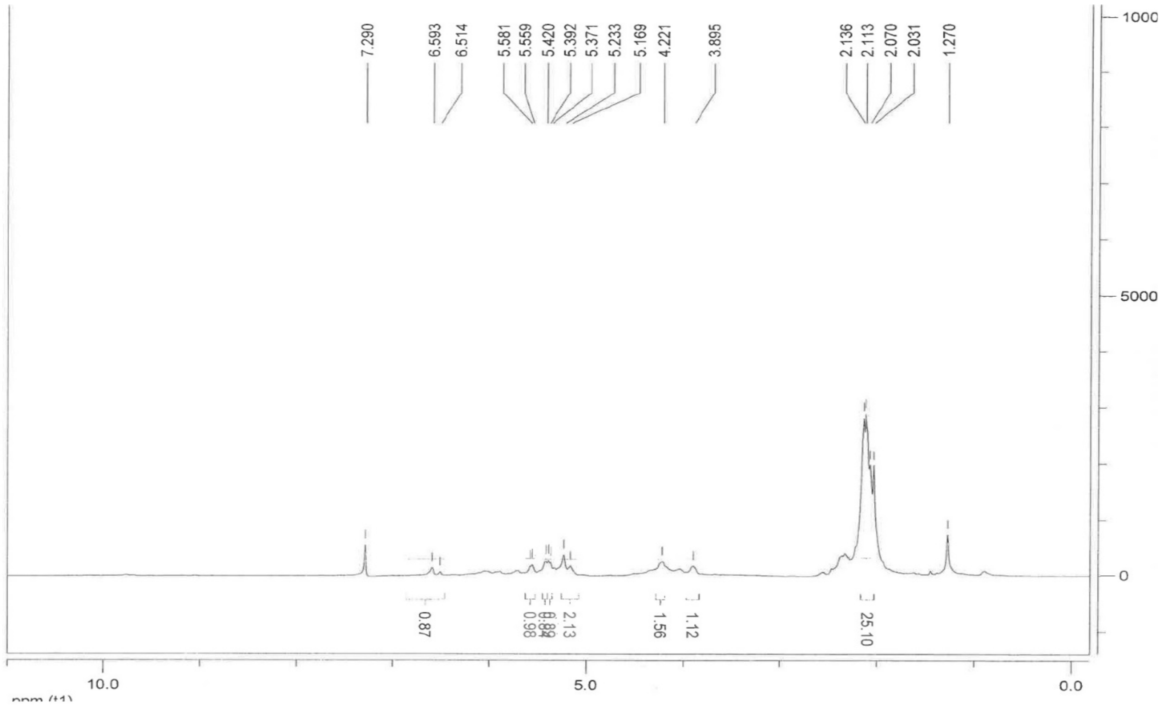

Figure S27. ^{1}H-NMR Spectrum of compound **7** ($CDCl_3$, 400 MHz).

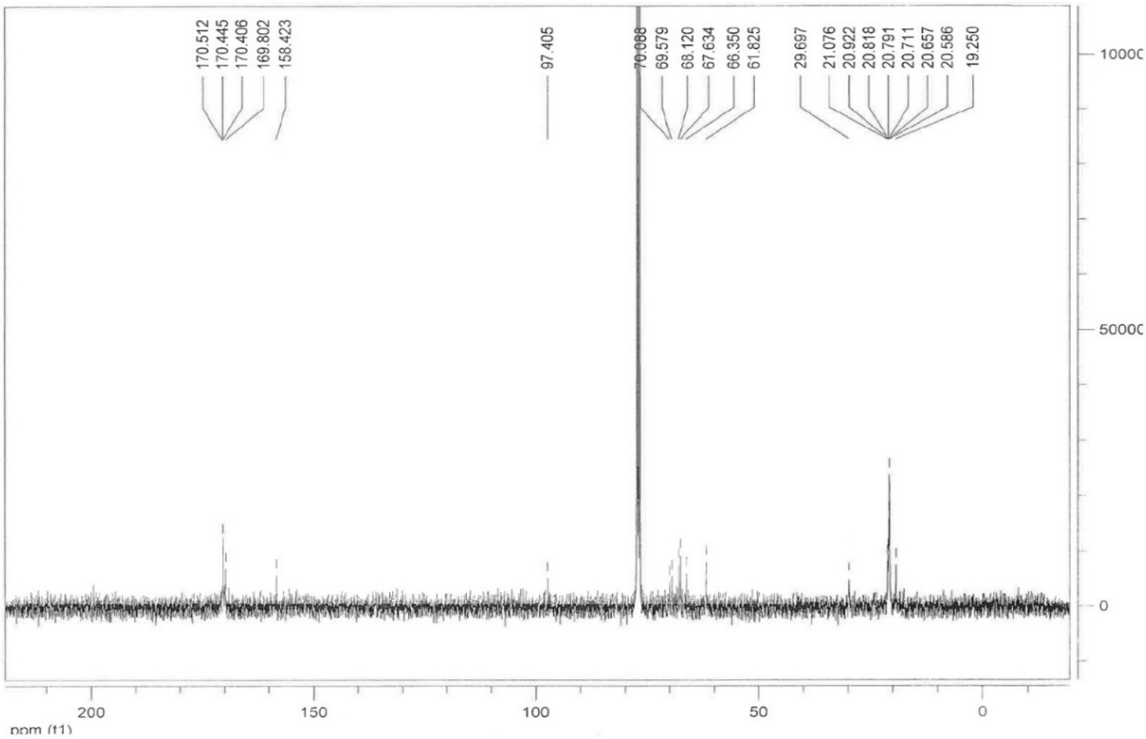

Figure S28. ^{13}C-NMR Spectrum of compound **7** ($CDCl_3$, 100 MHz).

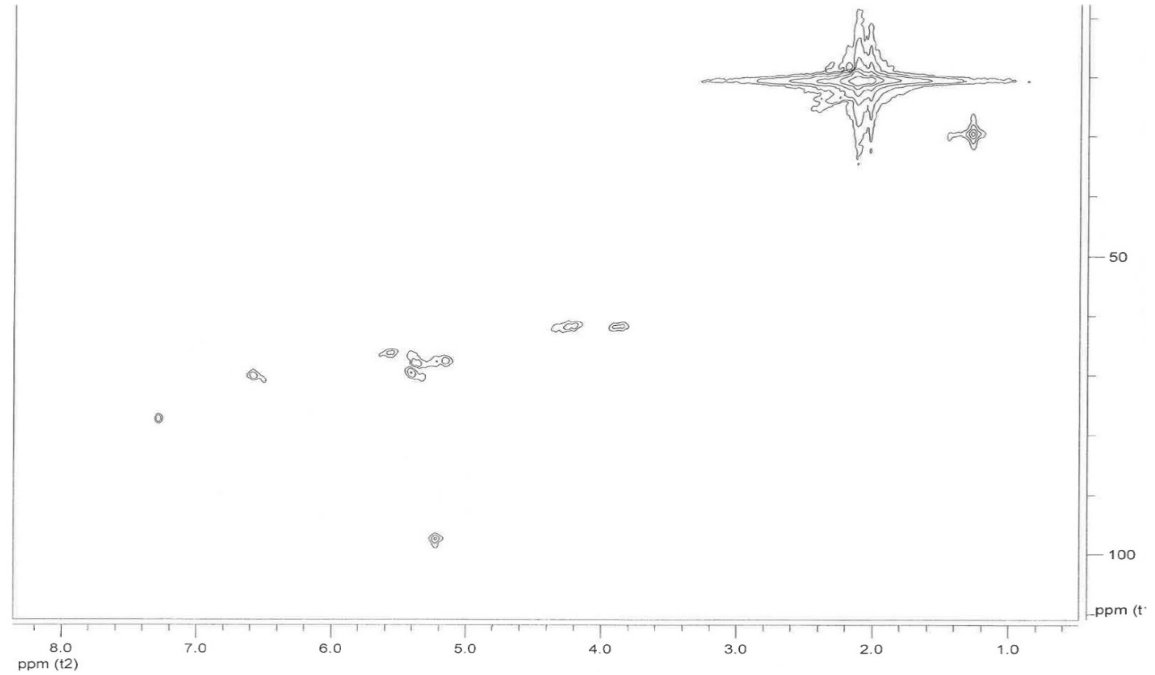

Figure S29. ^{1}H-^{13}C NMR (HMQC) Spectrum of compound **7** ($CDCl_3$, 400 MHz).

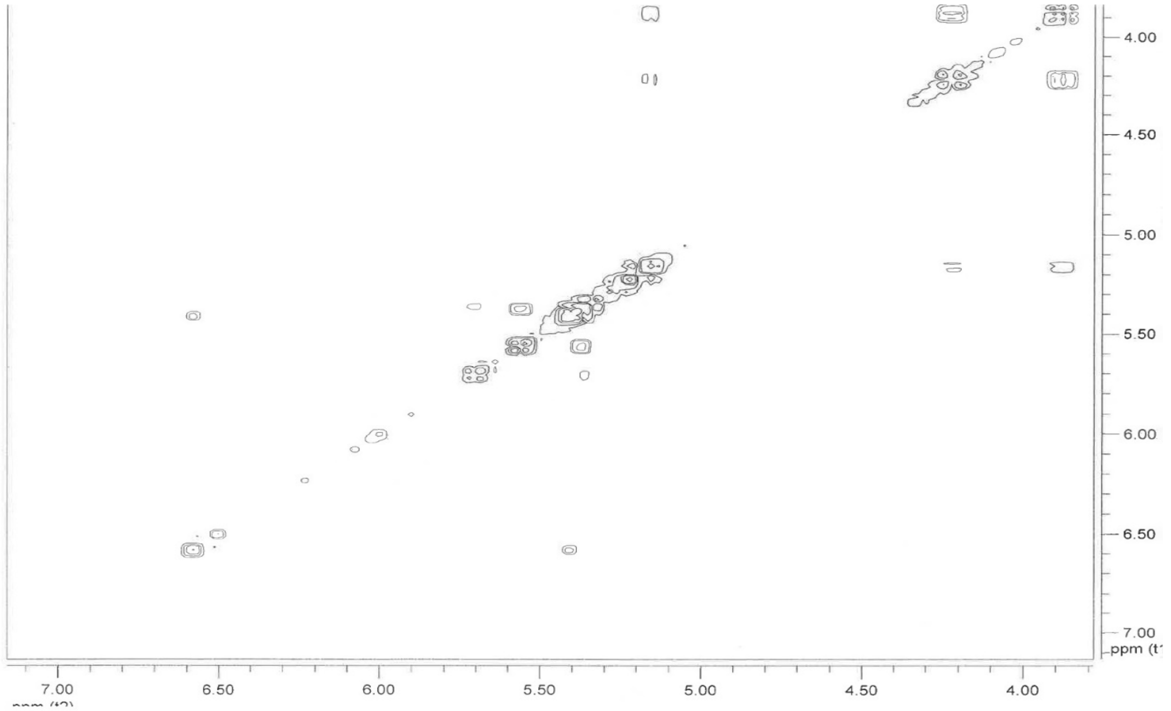

Figure S30. ^{1}H-^{1}H-NMR (DQF COSY) Spectrum of compound **7** ($CDCl_3$, 400 MHz).

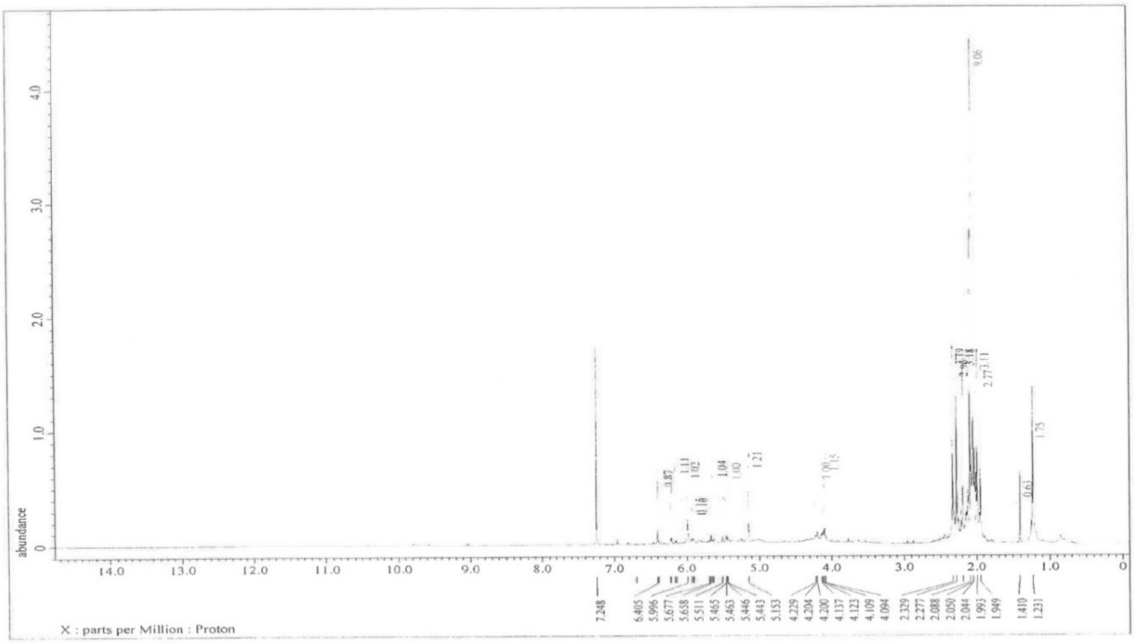

Figure S31. ^{1}H-NMR Spectrum of compound **8A/B** ($CDCl_3$, 500 MHz).

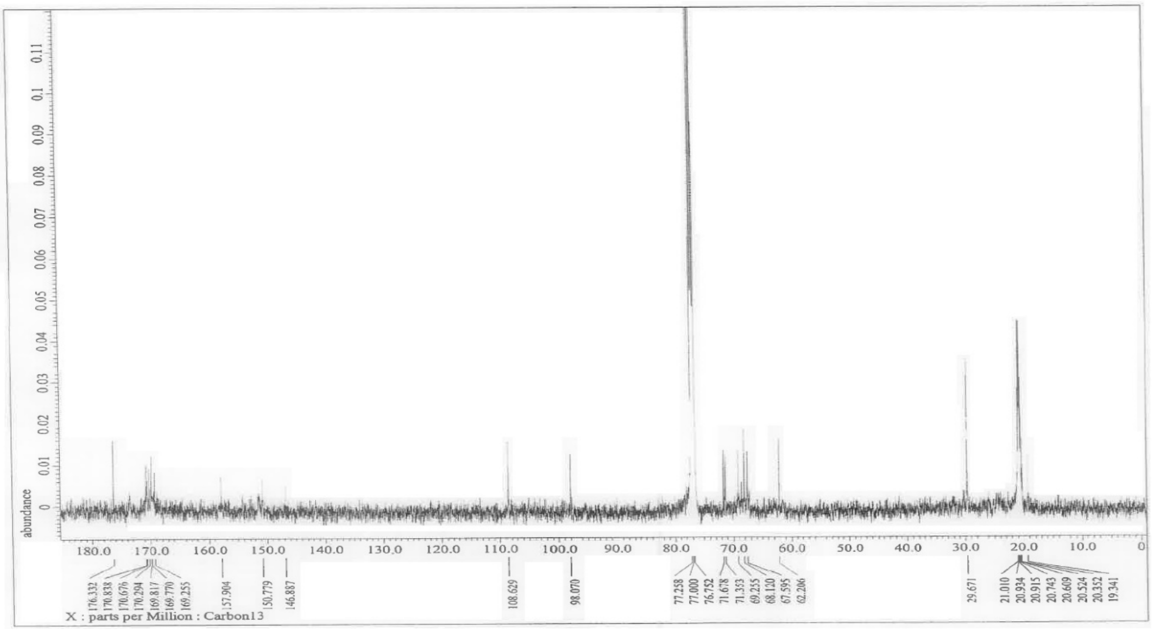

Figure S32. ^{13}C-NMR Spectrum of compound **8A/B** ($CDCl_3$, 125 MHz).

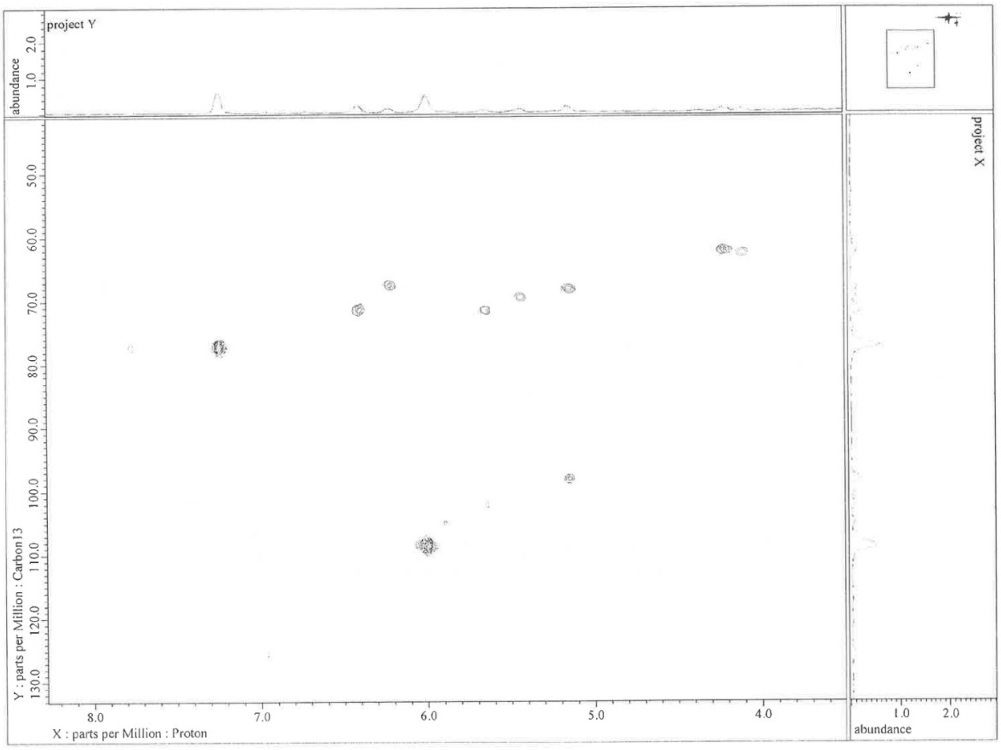

Figure S33. ^{1}H-^{13}C NMR Spectrum of compound **8A/B**, an expansion to the sugar moiety ($CDCl_3$, 500 MHz).

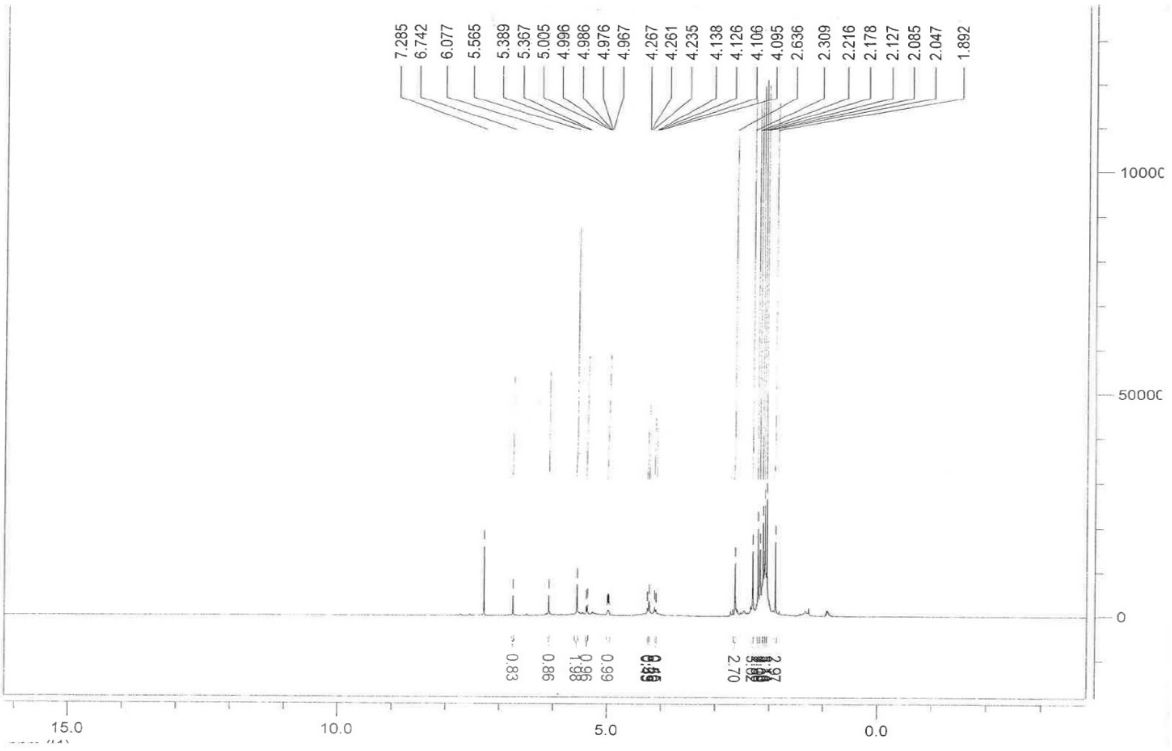

Figure S34. ^{1}H-NMR Spectrum of compound **9A** ($CDCl_3$, 400 MHz).

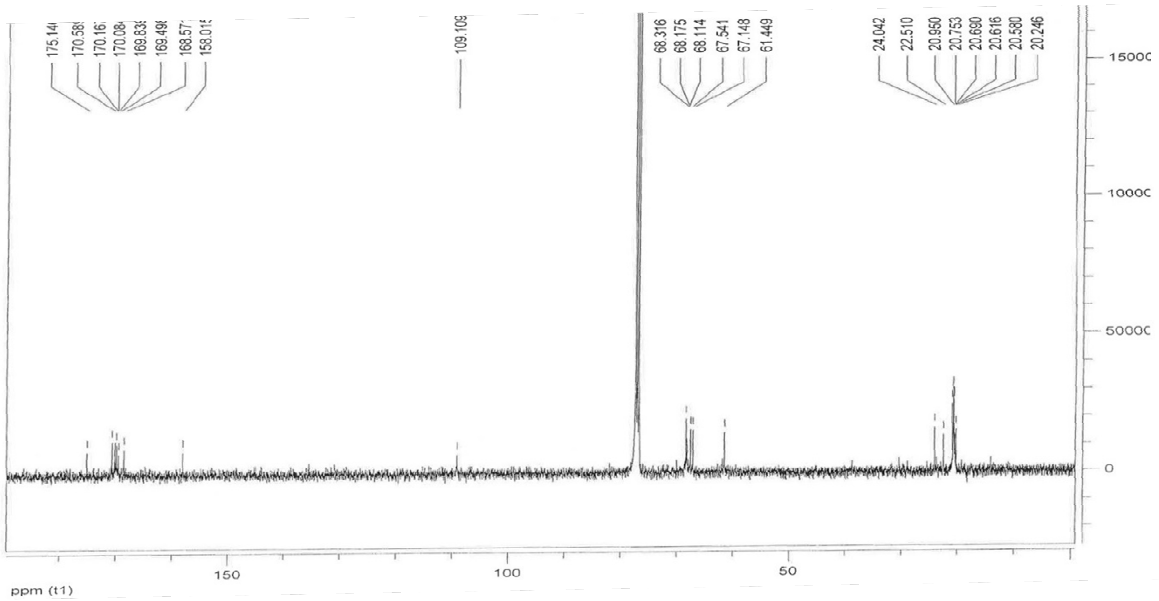

Figure S35. ^{13}C-NMR Spectrum of compound **9A** ($CDCl_3$, 100 MHz).

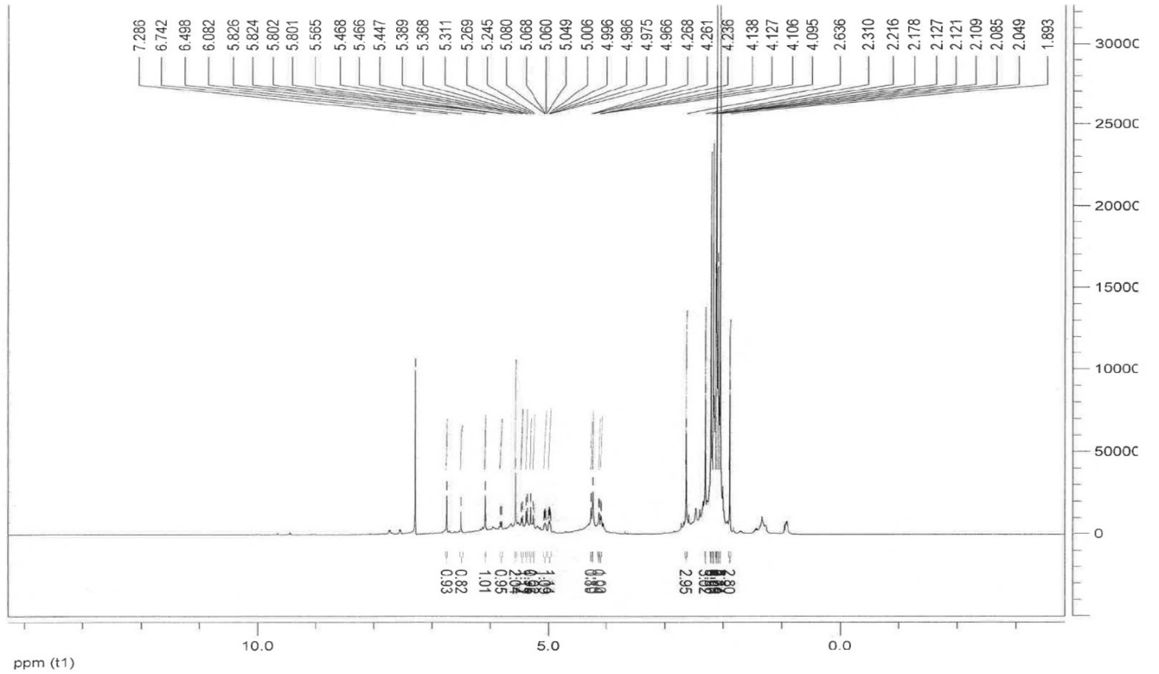

Figure S36. ^{1}H-NMR Spectrum of compound **9A** after exposure to light ($CDCl_3$, 400 MHz).

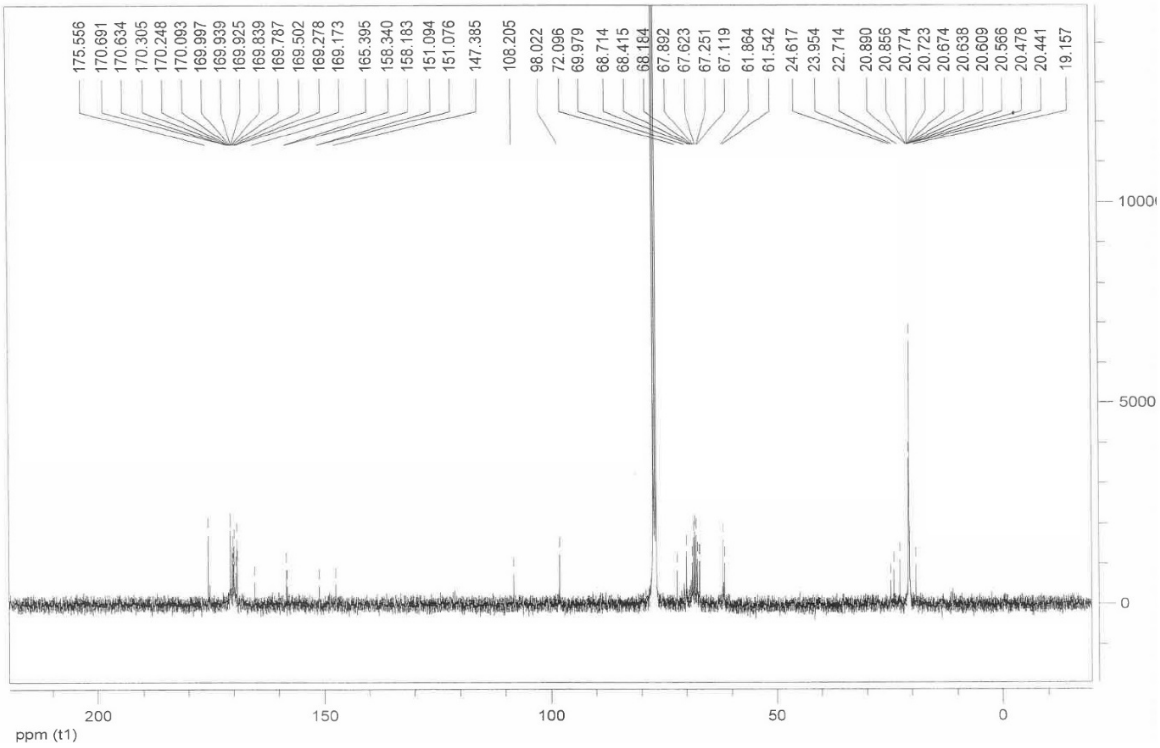

Figure S37. ^{13}C-NMR Spectrum of compound **9A** after exposure to light ($CDCl_3$, 100 MHz).

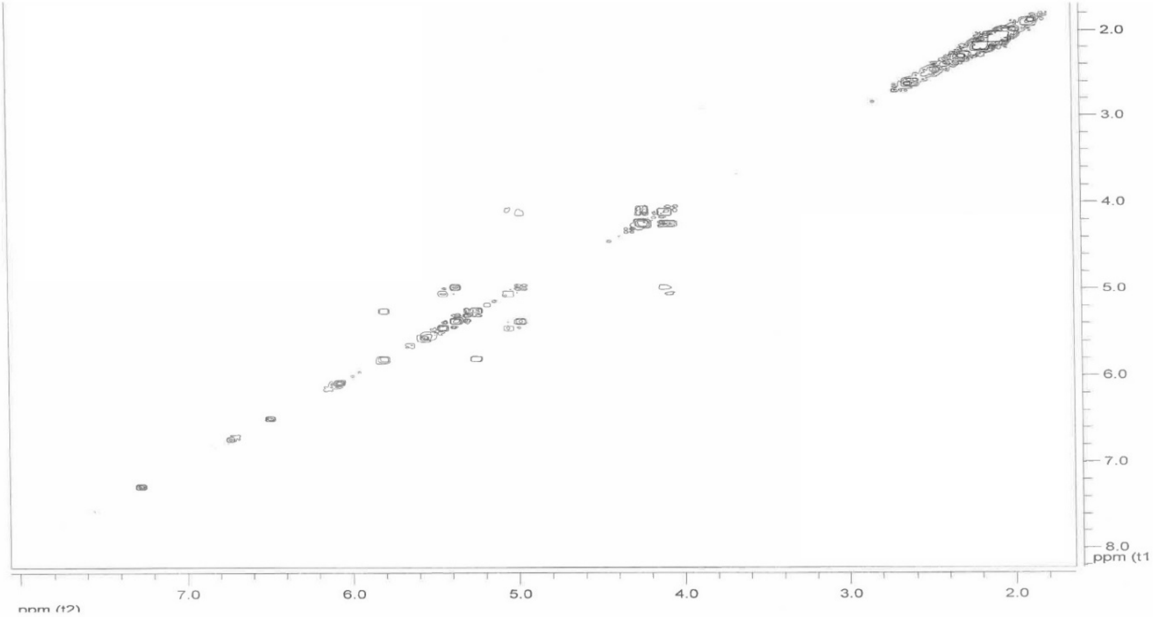

Figure S38. ^1H-^1H NMR (DQF COSY) Spectrum of compound **9A** after exposure to light ($CDCl_3$, 400 MHz).

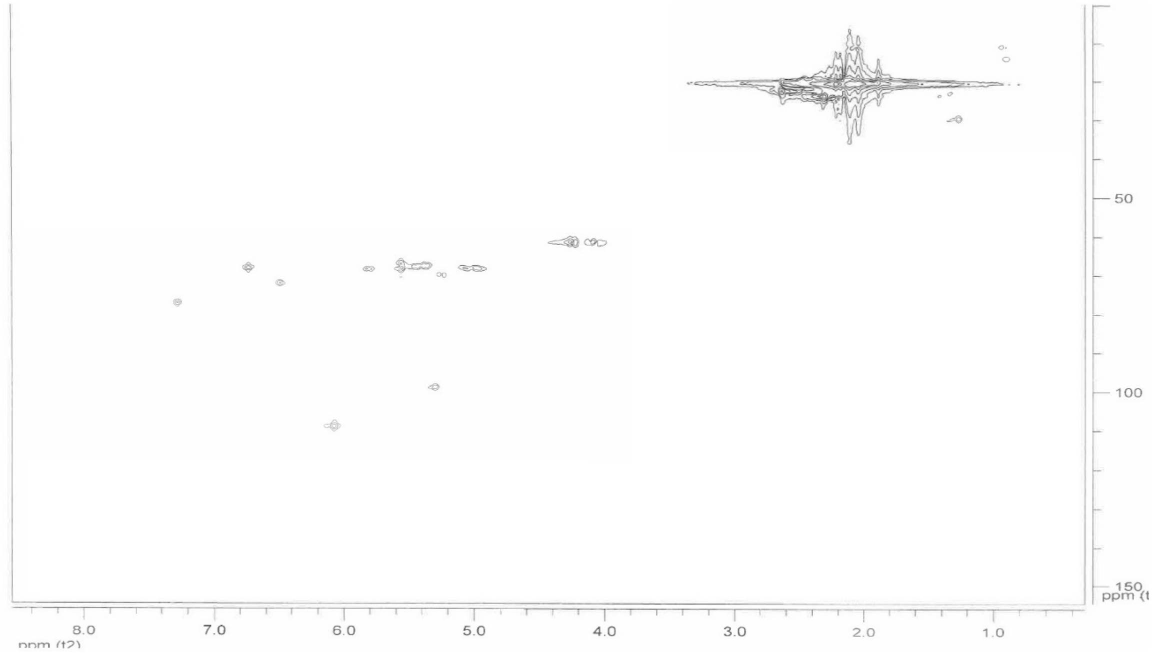

Figure S39. ^1H-^{13}C NMR (HMQC) Spectrum of compound **9A** after exposure to light, ($CDCl_3$, 400 MHz).

2. Biological evaluation

2.1. Cytotoxicity screening on normal human lung fibroblasts (Wi-38)

The normal human lung fibroblast Wi-38 cell line was used to detect cytotoxicity of the studied compounds. Wi-38 cell line was cultured in DMEM medium-contained 10 % fetal bovine serum (FBS), seeded as $5x10^3$ cells per well in 96-well cell culture plate, and incubated at 37 ºC in 5 % CO_2 incubator. After 24 h for cell attachment, serial concentrations (25-800 nM) of the synthetic compounds and currently used chemotherapy (doxorubicin) were incubated with Wi-38 cells for 72 h. Cell viability was assayed by the MTT method [1,2]. Twenty microliters of 5 mg/ml MTT (Sigma, USA) were added to each well and the plate was incubated at 37 ºC for 3 h. Then MTT solution was removed, 100 µl DMSO was added and the absorbance of each well was measured with a microplate reader (BMG LabTech, Germany) at 570 nm. Plot of Wi-38 cell viability (%) and growth inhibition (%) of the treated cells with serial concentrations (µM) of the most active compounds (8 and 9) and the reference chemotherapy (Dox) is illustrated in figure 39-A. The effective concentrations at which 100% and 50% cell viability (EC_{100} and IC_{50}, respectively) of the tested compounds was estimated by the Graphpad Instat software. Additionally, morphology of Wi-38 before and after exposure with the most effective anticancer compounds were investigated using phase-contrast inverted microscope with a digital camera (Olympus, Japan).

2.2. Determination of the anticancer activity

The anticancer effect of the above-mentioned compounds, in comparison with doxorubicin (Dox), was assayed using three human cancer cell lines. Colon cancer cell line (Caco-2) was cultured in DMEM (Lonza, USA) contained with 10 % FBS while liver cancer cell line (HepG-2) and triple-negative breast cancer cell line (MDA-MB 231) were cultured in RPMI-1640 (Lonza, USA) supplemented with 10 % FBS. All cancer cells ($4x10^3$ cells/well) were seeded in sterile 96-well plates. After 24h, serial concentrations (0.2-125 nM) of the tested compounds were incubated with three cancer cell lines for 72 h at 37 ºC in a 5 % CO_2 incubator. MTT method was done as described above [1,2]. Plots of cancer cells viability (%) and growth inhibition (%) of the studied cancer cell lines with serial concentrations (µM) of the most active compounds (8 and 9) and Dox are illustrated in figure 39.

The half-maximal inhibitory concentration (IC_{50}) values were calculated using the Graphpad Instat software. Furthermore, cellular morphological changes before and after treatment with the most effective and safest anticancer compounds were investigated using phase-contrast inverted microscope with a digital camera (Olympus, Japan). Moreover, selectivity index (SI) of the most active compounds was estimated as ratio of IC_{50} value of normal cells to IC_{50} value of each studied cancer cell lines.

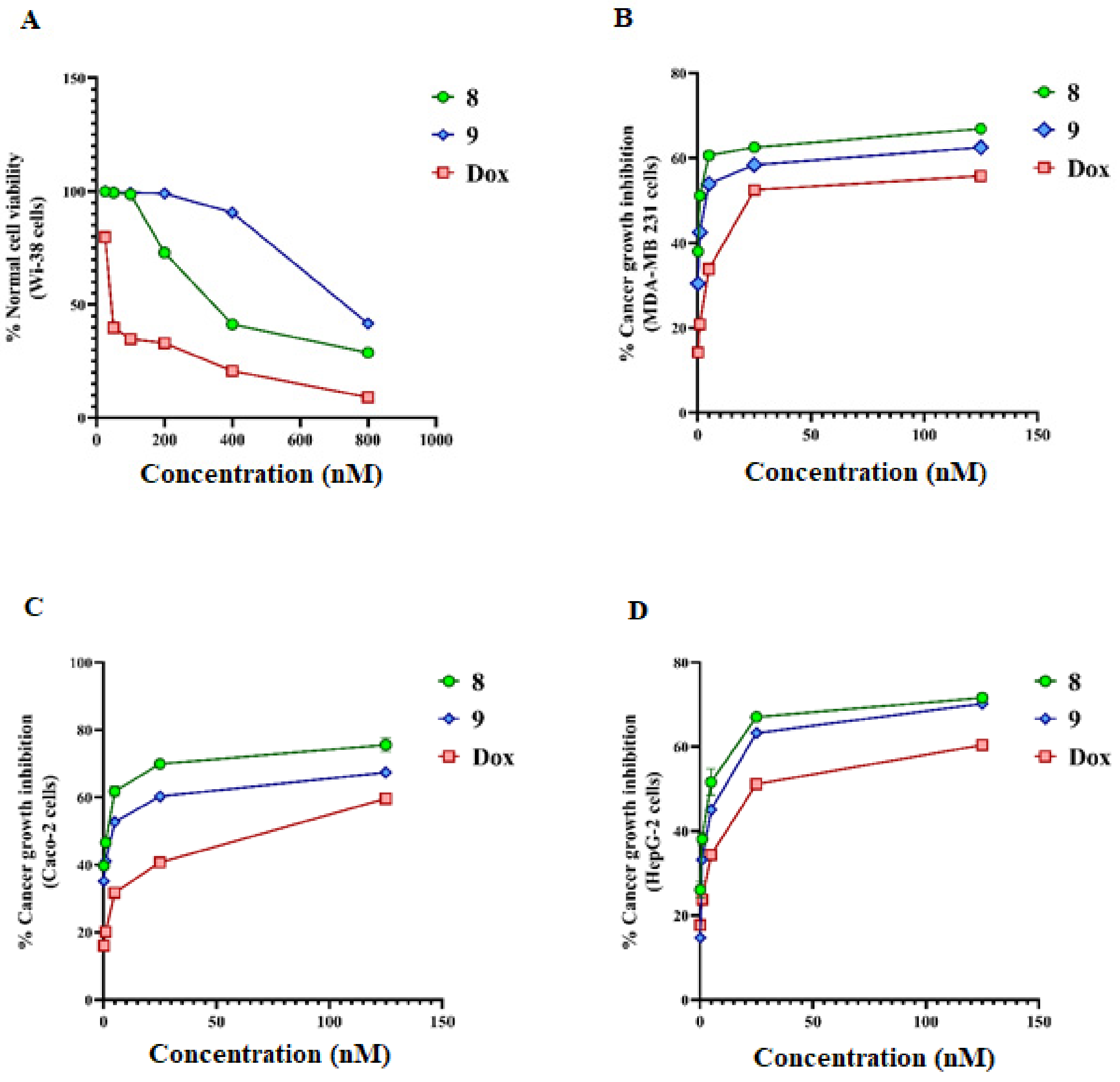

Figure S40. Plot of (A) Wi-38 cell viability (%) and (B-D) growth inhibition (%) of cancer cell lines with serial concentrations (nM) of the most active compounds (**8** and **9**) and the reference chemotherapy (Dox).

2.3. VEGFR-2 kinase inhibitory activity assay

The most active compounds were evaluated for in vitro VEGFR-2 kinase inhibitory activity by VEGFR2 (KDR) Kinase Assay Kit-BPS Bioscience Corporation catalog # 40325, using Kinase-Glo® MAX as a detection reagent following the manufacturer's instructions [3] as following:

- Thaw 5x Kinase Buffer 1, ATP and PTK substrate Poly (Glu:Tyr 4:1) (10 mg/ml).
- Prepare the master mixture (25 µl per well): N wells x (6 µl 5x Kinase Buffer 1 + 1 µl ATP (500 µM) + 1 µl PTK substrate Poly (Glu:Tyr 4:1) (10 mg/ml)+ 17 µl water). Add 25 µl to every well.
- Add 5 µl of Inhibitor solution of each well labeled as "Test Inhibitor". For the "Positive Control" and "Blank", add 5 µl of the same solution without inhibitor (Inhibitor buffer).
- Prepare 3 ml of 1x Kinase Buffer 1 by mixing 600 µl of 5x Kinase Buffer 1 with 2400 µl water. 3 ml of 1x Kinase Buffer 1 is sufficient for 100 reactions.
- To the wells designated as "Blank", add 20 µl of 1x Kinase Buffer 1.
- Thaw VEGFR2 enzyme on ice. Upon first thaw, briefly spin tube containing enzyme to recover full content of the tube. Calculate the amount of VEGFR2 required for the assay and dilute enzyme to 1 ng/µl with 1x Kinase Buffer 1.
- Initiate reaction by adding 20 µl of diluted VEGFR2 enzyme to the wells designated "Positive Control" and "Test Inhibitor Control". Incubate at 30°C for 45 minutes.
- Thaw Kinase-Glo Max reagent.
- After the 45 minutes, add 50 µl of Kinase-Glo Max reagent to each well. Cover plate with aluminum foil and incubate the plate at room temperature for 15 minutes.
- Measure luminescence using the microplate reader.
- Inhibitory activity was expressed as IC_{50} values (the concentration at which 50 % of the enzyme activity inhibited), which were calculated from dose-response curve obtained using eight tested concentrations of the inhibitor and carried out in duplicate.

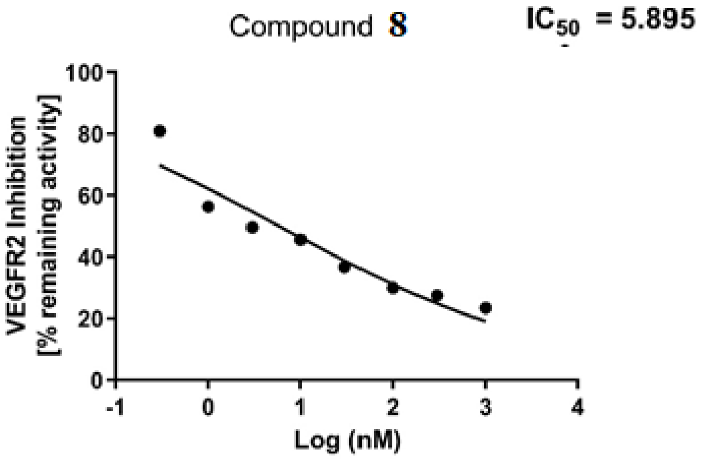

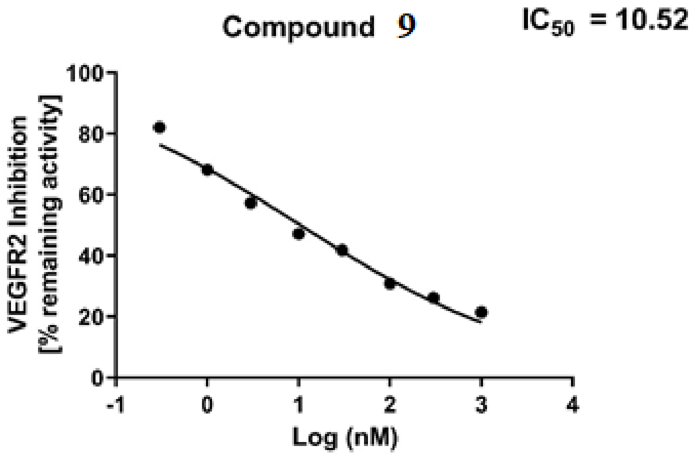

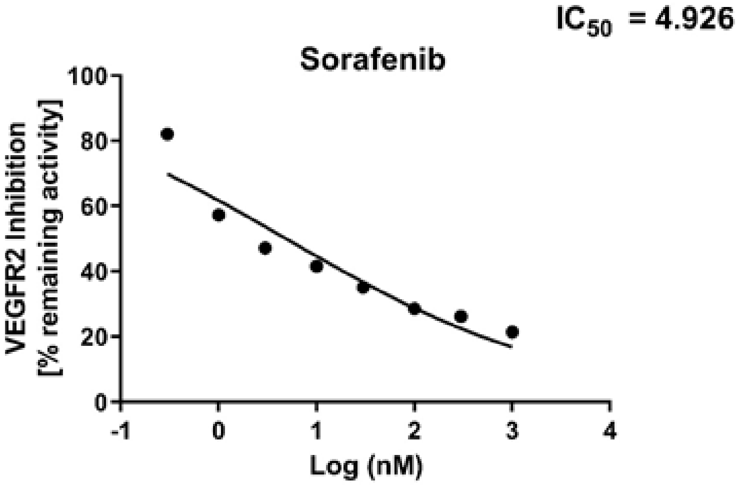

Figure S41. Dose-VEGFR2 inhibition curves of the hit compounds (8 and 9), and Sorafenib

2.4. MMP-2 inhibitory activity assay

The most active compounds were evaluated for in vitro MMP-2 inhibitory activities utilizing MMP-2 inhibitor screening kit (Colorimetric) catalog # ab139446 following the manufacturer's instructions [4]. The assay uses a thiopeptide as a chromogenic substrate. The MMP cleavage site peptide bond is replaced by a thioester bond in the thiopeptide. Hydrolysis of this bond by MMP-2 produces a sulfhydryl group, which reacts with Ellman's reagent to form 2-nitro-5-thiobenzoic acid that can be detected by its absorbance at 412 nm. The procedure of MMP-2 inhibition assay is as following

- Add test compounds and diluted enzyme solution to the microplate wells.
- For one well of 96-well plate, the suggested volume of enzyme solution is 40 μL and 10 μL of test compound.
- Simultaneously establish the following control wells.
- Vehicle control contains MMP enzyme and vehicle used in delivering test compound (DMSO, concentration not to exceed 1%).
- bring the total volume of all controls to 50 μL. Pre-incubate the plate for 10 min. at assay temperature to 37°C.
- Run the enzymatic reaction. Add 50 μL of MMP substrate solution into each well. Mix the reagents completely by shaking the plate gently for 30 sec.

- Incubate the reaction for 60 min. Add 50 µL of stop solution to each well. Mix the reagents and measure absorbance at 412 nm.
- Inhibitory activity was expressed as IC_{50} values (the concentration at which 50 % of the enzyme activity inhibited), which were calculated from dose-response curve obtained using eight tested concentrations of the inhibitor and carried out in duplicate.

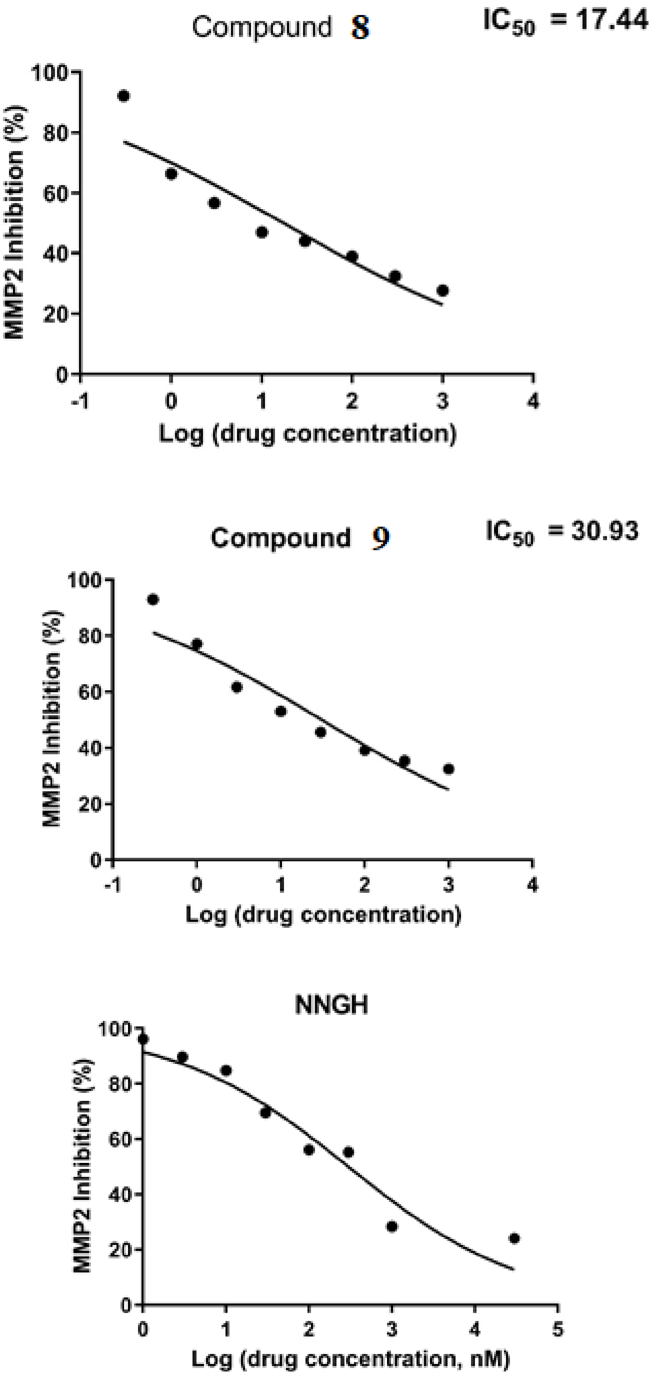

Figure S42. Dose-MMP2 inhibition curves of the hit compounds (**8** and **9**), and NNGH

2.5. CA II inhibition assay

CA II inhibition assay for the test compounds was performed based on measurement of the reduction in esterase activity on the substrate p-nitrophenyl acetate (4-NPA) that was used to perform CA II inhibition assay with a slight modification [5]. The procedure of CA II inhibition assay is as following:

- Total mixture volume was 200 µl in a well-contained 140 µl (20 mM HEPES), Tris buffer (pH 7.4), 20 µl of the enzyme (from bovine, Sigma–Aldrich, C2624), 20 µl (0.5 mg/ml in DMSO) of the test compounds, which were mixed and incubated at 25 °C for 15 min.
- After incubation, 20 µl of the substrate (4-nitrophenyl acetate, Sigma–Aldrich, N-8130; 0.7 mM in MeOH) was added.
- The reaction was run under the same conditions for 60 min. and the final read was taken at 405 nm.
- Quercetin was used as the reference (positive control).
- The hydrolysis of the substrate was then evaluated at 405 nm using a Microplate Elisa Reader.
- Results obtained after the reactions done in triplicate were measured by the equation given below.
  The percent inhibition for each sample was calculated as:
  Inhibition (%) = {(C - T)/C}100 .... (1), where C (i.e., control) = total enzyme activity without inhibitor and T (i.e., test sample) = activity in the presence of test compound.
- Inhibitory activity was expressed as IC_{50} values (the concentration at which 50 % of the enzyme activity inhibited), which were calculated from dose-response curve obtained using ten tested concentrations of the inhibitor and carried out in triplicate.

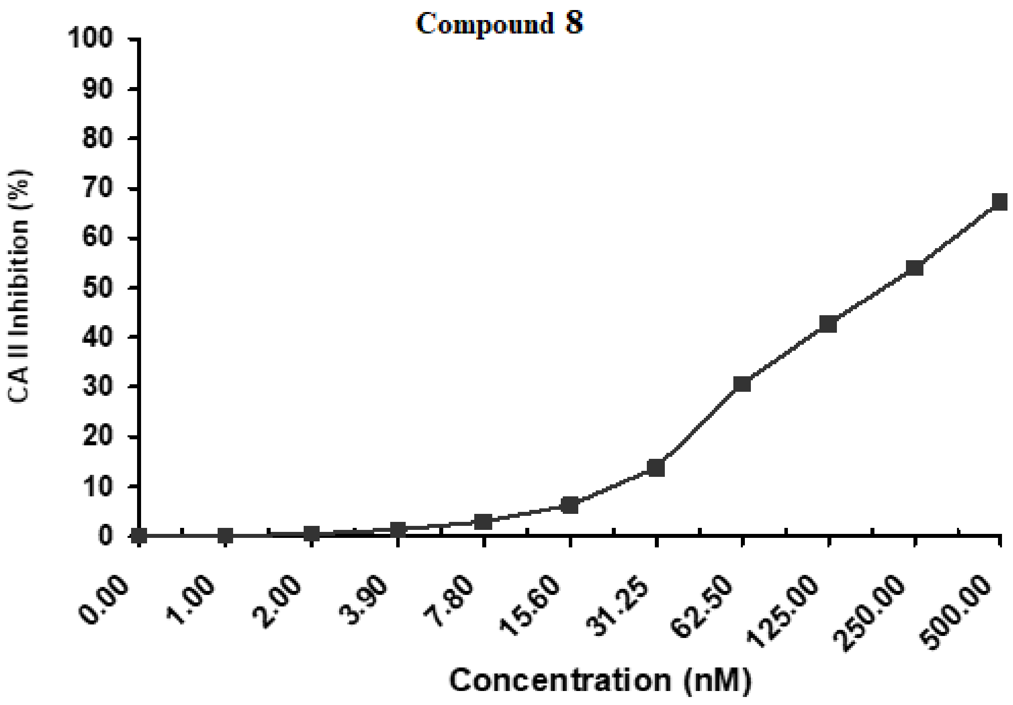

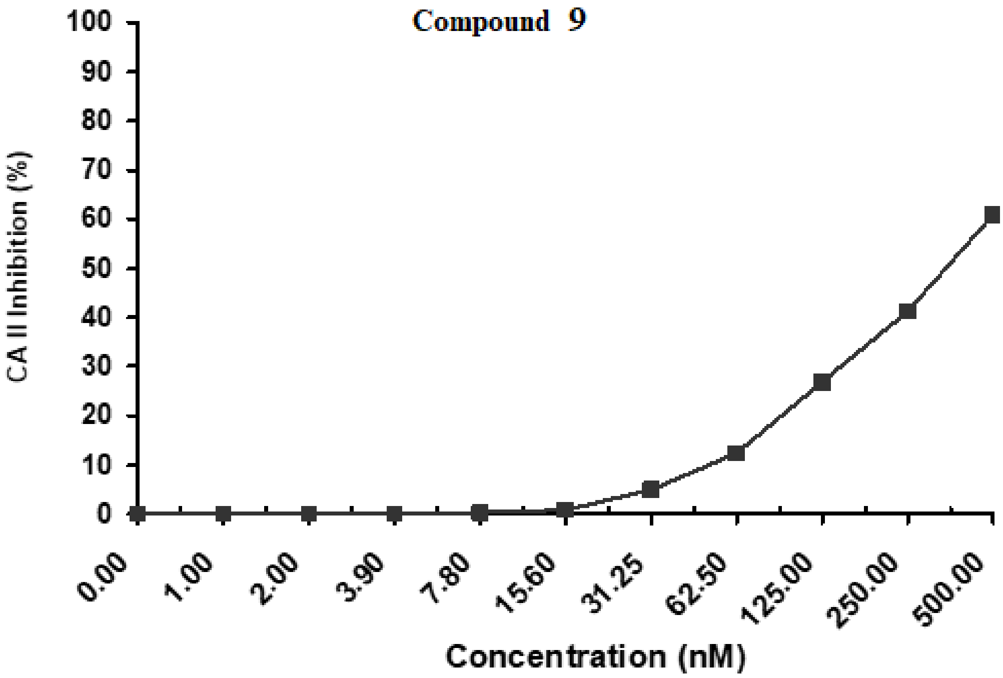

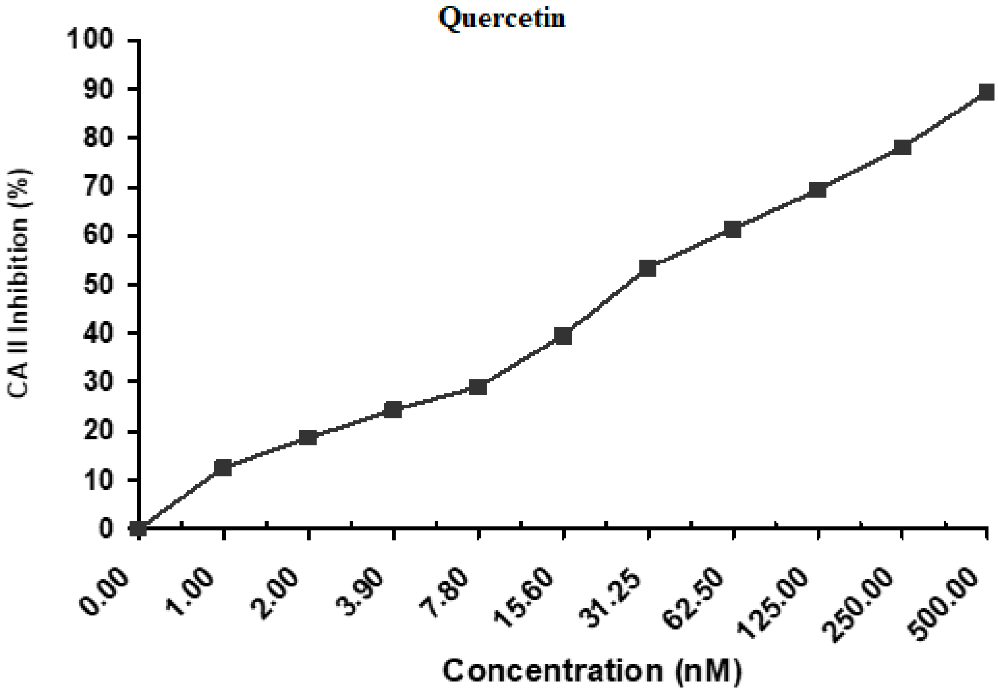

Figure S43. Dose-CAII inhibition curves of the hit compounds (**8** and **9**), and quercetin

3. Docking simulation

The structures of the compounds were energy minimized using MMFF94x forcefield and gradient: 0.05. During energy minimization, hydrogens were added, and partial charges were applied. Molecular

docking was performed by MOE using Triangle Matcher placement method, Rescoring1: London dG, Refinement: Forcefield, and Rescoring2: Affinity dG. As a default parameter, 20 docked conformations were selected to be saved for each compound after docking. After docking, each conformation of all the docked compounds were visualized and based on the protein–ligand interactions, docking score, and best conformation was selected. The images in 2D and 3D were captured through MOE ligand binding interaction.

References

1. O.H. Rizk, M. Teleb, M.M. Abu-Serie and O.G. Shaaban, *Bioorganic chemistry*, 2019, ***92***, 103189.
2. M.S. Ayoup, Y. Wahby, H. Abdel-Hamid, M. Teleb, M.M. Abu-Serie and A. Noby, *European Journal of medicinal chemistry*, 2019, ***168***, 340.
3. http://bpsbioscience.com/vegfr2-kdr-kinase-assay-kit-40325.
4. https://www.abcam.com/mmp2-inhibitor-screening-assay-kit-colorimetric-ab139446.html
5. H. Huang, X. Pan, C. Ji, G. Zeng, L. Jiang, X. Fu, J. Liu, X. Hao, Y. Zhang and N. Tan, *Science in China Series B: Chemistry*, 2009, ***52***(3), 332.
